# YAP Signaling Regulates the Cellular Uptake and Therapeutic Effect of Nanoparticles

**DOI:** 10.1002/advs.202302965

**Published:** 2023-11-09

**Authors:** Marco Cassani, Soraia Fernandes, Jorge Oliver‐De La Cruz, Helena Durikova, Jan Vrbsky, Marek Patočka, Veronika Hegrova, Simon Klimovic, Jan Pribyl, Doriana Debellis, Petr Skladal, Francesca Cavalieri, Frank Caruso, Giancarlo Forte

**Affiliations:** ^1^ International Clinical Research Center St. Anne's University Hospital Brno 60200 Czech Republic; ^2^ Department of Chemical Engineering The University of Melbourne Parkville Victoria 3010 Australia; ^3^ Institute for Bioengineering of Catalonia (IBEC) The Barcelona Institute for Science and Technology (BIST) Barcelona Spain; ^4^ NenoVision Purkynova 649/127 Brno 61200 Czech Republic; ^5^ Faculty of Mechanical Engineering Brno University of Technology Technicka 2896/2 Brno 61669 Czech Republic; ^6^ Department of Bioanalytical Instrumentation CEITEC Masaryk University Brno 60200 Czech Republic; ^7^ Electron Microscopy Facility Fondazione Istituto Italiano Di Tecnologia Via Morego 30 Genoa 16163 Italy; ^8^ School of Science RMIT University Melbourne 3000 Victoria Australia; ^9^ Dipartimento di Scienze e Tecnologie Chimiche Università di Roma “Tor Vergata” Via Della Ricerca Scientifica Rome 00133 Italy; ^10^ School of Cardiovascular and Metabolic Medicine & Sciences King's College London London WC2R 2LS UK

**Keywords:** bio‐nano interactions, cancer treatment, mechanobiology, nanoparticles, YAP‐signaling

## Abstract

Interactions between living cells and nanoparticles are extensively studied to enhance the delivery of therapeutics. Nanoparticles size, shape, stiffness, and surface charge are regarded as the main features able to control the fate of cell‐nanoparticle interactions. However, the clinical translation of nanotherapies has so far been limited, and there is a need to better understand the biology of cell‐nanoparticle interactions. This study investigates the role of cellular mechanosensitive components in cell‐nanoparticle interactions. It is demonstrated that the genetic and pharmacologic inhibition of yes‐associated protein (YAP), a key component of cancer cell mechanosensing apparatus and Hippo pathway effector, improves nanoparticle internalization in triple‐negative breast cancer cells regardless of nanoparticle properties or substrate characteristics. This process occurs through YAP‐dependent regulation of endocytic pathways, cell mechanics, and membrane organization. Hence, the study proposes targeting YAP may sensitize triple‐negative breast cancer cells to chemotherapy and increase the selectivity of nanotherapy.

## Graphical abstract

The inhibition of Yeas‐associated protein (YAP) in TNBC cells affects the organization of plasma membrane, reduces the extracellular matrix (ECM) deposition, impacts their adhesion ability, and increases the endocytosis rate. Thus, targeting cell mechanobiology may be leveraged to optimize cell‐nanoparticle interactions. Ultimately, these changes contribute cooperatively to promote the delivery of nanomedicines to cancer cells and improve the therapeutic efficiency.

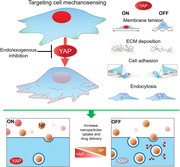

## 1 Introduction

Yes‐Associated Protein (YAP) is a mechanically activated downstream effector of the Hippo pathway,^[^ ^1^ ^]^ which plays a critical role in embryogenesis by controlling the size and shape of organs through the proliferation of embryonic parenchymal cells, such as cardiomyocytes and hepatocytes.^[^ ^2^, ^3^ ^]^ Similar to its paralog protein TAZ, which is encoded by the WWTR1 gene, YAP acts in a pleiotropic fashion by interacting with tissue‐ and stage‐specific transcription factors,^[^ ^4^, ^5^ ^]^ primarily those of the TEAD family.^[^ ^6^, ^7^, ^8^, ^9^ ^]^ YAP is commonly overexpressed in many solid tumors including breast, lung, colorectal, pancreatic, and liver carcinomas, as well as melanoma and glioma, during their growth, progression and metastasis.^[^ ^10^, ^11^, ^12^, ^13^ ^]^ YAP has been shown to promote tumor survival by driving tumor immune evasion through the activation of PD‐L1 transcription and by rewiring macrophage response to a pro‐tumor phenotype.^[^ ^14^ ^]^ Additionally, it appears to inhibit autophagy‐related cell death,^[^ ^15^ ^]^ and drive tumor resistance to targeted therapy and chemotherapy, supposedly through the stimulation of pro‐survival and anti‐apoptotic genes.^[^ ^14^ ^]^ YAP overexpression has been linked to poor prognosis and survival in patients with breast cancer, as well as in other tumor types.^[^ ^16^, ^17^ ^]^

Our group has recently demonstrated that YAP‐mediated activation of cell adhesion genes drives the stiffening of CAL51 triple‐negative breast cancer (TNBC) cells.^[^ ^18^ ^]^ TNBC defines a subtype of breast cancer characterized by aggressive behavior, frequent relapses, and resistance to chemotherapy.^[^ ^19^, ^20^ ^]^ We have subsequently shown that targeting YAP via mechanical, pharmacological, or genetic strategies prevents breast cancer cells from undergoing epithelial‐to‐mesenchymal transition (EMT) and migration, favoring instead the acquisition of a terminally differentiated phenotype of adipocytes.^[^ ^21^ ^]^

Increased extracellular matrix (ECM) stiffness is a common feature of solid tumors,^[^ ^22^, ^23^ ^]^ and the expression of EMT transition markers is often used to gauge the aggressiveness of breast cancer.^[^ ^24^ ^]^ Interestingly, YAP hyperactivation has been recently discovered to play a key role in enabling cancer‐associated fibroblasts (CAFs) to induce tumor stroma stiffening and promote malignant cell invasion and metastatization.^[^ ^25^ ^]^ Given its dual role in CAFs and tumor cells as both a mechanosensitive protein and a proto‐oncogene, YAP is now viewed as a critical component in the positive feedback loop that leads to stroma stiffening and cancer dissemination. Recently, manipulating the mechanical properties of cells or substrates has been proposed as a plausible strategy to control nanoparticle binding and internalization, hence paving the way to using nanoparticles for the mechanotargeting of primary or metastatic cancer cells.^[^ ^26^, ^27^ ^]^ Considering this, understanding the interactions between biological systems and nanomaterials has become a major goal of nanomedicine, with the aim of designing nanomaterials that can effectively interact with living cells.^[^ ^28^, ^29^ ^]^

The design and effectiveness of nanomedicines for cancer therapy depend on various physicochemical properties of the nanocarrier, including its size, shape, stiffness, and surface chemistry.^[^ ^30^, ^31^ ^]^ Much research has focused on optimizing these properties to enhance cell‐nanoparticle interaction and improve anti‐cancer drug delivery.^[^ ^32^, ^33^ ^]^ Notwithstanding significant advances in understanding bio‐nano interactions over the last 20 years, much remains unknown about the exact mechanisms underlying cell‐nanoparticle interactions.^[^ ^34^ ^]^ Nevertheless, unveiling the processes responsible for these interactions at the molecular level may lead to the development of new strategies for enhancing nanoparticle delivery to specific cells of interest.^[^ ^35^ ^]^

Despite YAP is being proposed as a target for novel treatments,^[^ ^36^, ^37^ ^]^ its response to nanoparticles internalization and its potential role in their trafficking has never been investigated. In the present study, we used TNBC cells to unveil the role of YAP in cell‐nanoparticle interactions and show its potential in regulating nanoparticle internalization via the control of membrane organization and cell mechanics. We demonstrate that YAP is responsible for the transcription of genes regulating cell‐matrix interactions, ECM deposition, and endocytic pathways in breast cancer cells, and show that its inhibition boosts the internalization of nanoparticles. Finally, we delineate how the intracellular delivery of doxorubicin‐loaded liposomes can be enhanced by pharmacologically tampering with YAP activity. In conclusion, by identifying Hippo effector as a determinant of cell‐nanoparticle interaction, we propose its inhibition as a viable therapeutic strategy for improving nanodrug delivery to triple‐negative breast cancer cells.

## 2 Results and Discussion

### 2.1 YAP Depletion Affects the Mechanical, Physical Properties, and the Membrane Organization of CAL51 TNBC Cells

**Figure 1:**
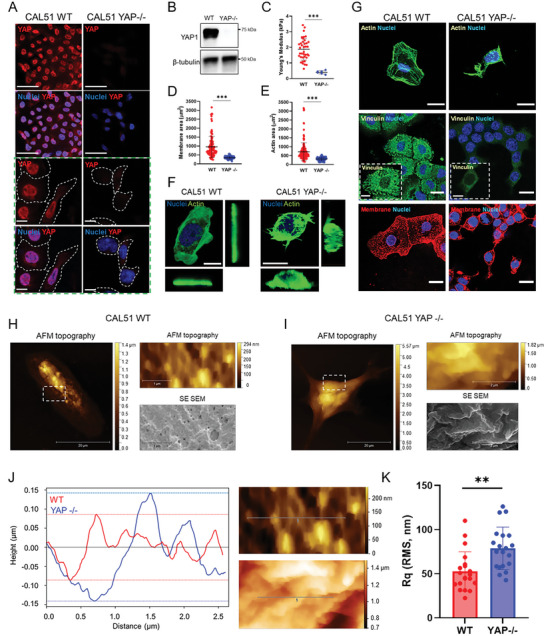
YAP depletion affects CAL51 adhesion, mechanics, morphology, and membrane properties. A) Representative confocal images depicting YAP expression in WT or YAP ‐/‐ CAL51 cells. Cells were stained for YAP (AF555, red), and nuclei were counterstained with DAPI (blue). Scale bar: 50 µm. The green dashed line box shows higher magnification pictures. Scale bar: 10 µm. B) Western blot analysis showing the levels of YAP protein in WT or YAP ‐/‐ CAL51 cells. β‐tubulin was used for protein loading normalization. C) Dot plot representation of the Young's modulus analysis of WT or YAP ‐/‐ CAL51 cells as measured by atomic force microscopy (AFM). WT CAL51: *n* = 80; YAP ‐/‐ CAL51: *n* = 10. Statistical analysis was performed by unpaired t‐test with Welch's correction; ^***^ *p* < 0.001. D) Dot plot analysis of WT or YAP ‐/‐ CAL51 total membrane area. Cells were stained with Alexa Fluor 488‐labeled wheat germ agglutinin (WGA‐488, green). *n* > 100 cells. Statistical analysis was performed by unpaired *t*‐test with Welch's correction; ^***^ *p* < 0.001. E) Dot plot analysis of WT or YAP ‐/‐ CAL51 cell surface area calculated based on the total actin coverage of the cells. Cells were stained with Alexa Fluor 488‐labeled Phalloidin (Pha‐488, green). *n* > 100. Statistical analysis was performed by unpaired *t*‐test with Welch's correction; ^***^ *p* < 0.001. F) CAL51 WT (left) and YAP ‐/‐ cells (right) 3D reconstruction. Cells were stained with DAPI and WGA‐488 (green). Scale bar: 20 µm. G) Representative confocal images of WT or YAP ‐/‐ CAL51 cells stained for nuclei (DAPI, blue) and actin (Pha‐488, green, top), vinculin (AF488, green, middle), and membrane (WGA‐647, red, bottom), respectively. Scale bar: 20 µm. The insets display high‐magnification images. Scale bar: 10 µm. Correlative Probe and Electron Microscopy (CPEM) imaging of CAL51 WT (H) and CAL51 YAP ‐/‐ (I) cells. AFM and SEM images are shown. White dashed line boxes indicate the detail of the magnifications shown as AFM and SEM images on the right of each main micrograph. J) Plot displaying the profile of the membrane roughness as determined for WT (red) and YAP ‐/‐ (blue) CAL51 cells in the region highlighted in the SEM images on the right (red dashed line, top for CAL51 WT; blue dashed line, bottom for YAP ‐/‐ CAL51). The roughness profile was calculated on the deconvolved images. Scale bar: 0.5 µm. K) Mean square roughness of the height irregularity (*R*_q_) measured on WT (red) and YAP ‐/‐ (blue) CAL51cells. *n* = 20. Statistical analysis was performed by unpaired t‐test with Welch's correction; ^**^ *p* < 0.01.

We first investigated the effect of YAP depletion on the morphology of CAL51 TNBC cells, which are characterized by constitutively high levels of YAP expression and activity.^[^ ^21^ ^]^ Using CRISPR/CAS9 technology, we generated a stable YAP‐deficient mutant CAL51 cell line (described in Ref. [18]), and confirmed YAP depletion through confocal microscopy (**Figure** **1**A) and western blot analyses (Figure 1B). Next, we used atomic force microscopy (AFM) to determine the effect of YAP depletion on the mechanical properties of CAL51 cells and found a significant reduction in Young's modulus in YAP ‐/‐ cells (Figure 1C). Moreover, compared to CAL51 WT cells, YAP ‐/‐ cells displayed reduced surface area, both as determined by actin coverage and membrane extension (Figure 1D,E), as well as perturbed cell morphology, resulting from their inability to spread over the adhesion surface (Figure 1F; see Figures S1 and S2, Supporting Information). These changes were due to the failure of the mutant cells to assemble focal adhesions and form proper cytoskeleton (Figure 1G).^[^ ^18^, ^38^ ^]^ Importantly, YAP depletion did not affect the proliferation and viability of CAL51 TNBC cells (see Figure S3, Supporting Information).

Given the striking change in morphology and mechanics exhibited by YAP ‐/‐ CAL51 cells compared to the wild‐type control, we analyzed their membrane structure using Correlative Probe and Electron Microscopy (CPEM) by LiteScope. This technique combines AFM and scanning electron microscope (SEM) to characterize 3D surface in situ, estimate surface roughness, and perform height/depth profiling with precise AFM tip navigation.^[^ ^39^ ^]^ The analysis demonstrated that YAP depletion and the following decrease in membrane tension led to the remodeling of the plasma membrane in CAL51 TNBC cells and the emergence of dynamic features connected with extensive cellular reorganization (Figure 1H,I). Previous literature has reported the appearance of various membrane structures including blebs and vacuole‐like dilations upon the reduction of cell strain.^[^ ^40^ ^]^ In YAP ‐/‐ cells, we observed the emergence of ripple‐like deformations (Figure 1J), which increased the roughness of the cell membrane, as quantified through height irregularity (*R*_q_, Figure 1K).

### 2.2 YAP Transcriptional Activity Controls the Expression of Genes Involved in Membrane Organization and Endocytosis in CAL51 TNBC Cells

Given the substantial effects that YAP depletion played on the structure of cell membrane, we hypothesized that the changes in the membrane of YAP ‐/‐ cells might be reflected in their transcriptional landscape. We adopted RNA sequencing (RNA‐seq) to investigate the regulation of genes encoding for proteins involved in plasma membrane organization (see Figure S4, Supporting Information). Overall, RNA‐seq revealed a total of 4219 differentially expressed genes in YAP ‐/‐ cells compared to WT CAL51 cells, with 1925 of them being downregulated and 2294 upregulated following YAP depletion (see Figure S4a–c, Supporting Information). The most represented gene ontology (GO) annotations were connected to ECM organization, cell migration, and cell adhesion in WT cells (see Figure S4d, Supporting Information), while genes related to integral components of the plasma membrane were found among the cellular components in YAP ‐/‐ counterpart (see Figure S4e, Supporting Information).

**Figure 2:**
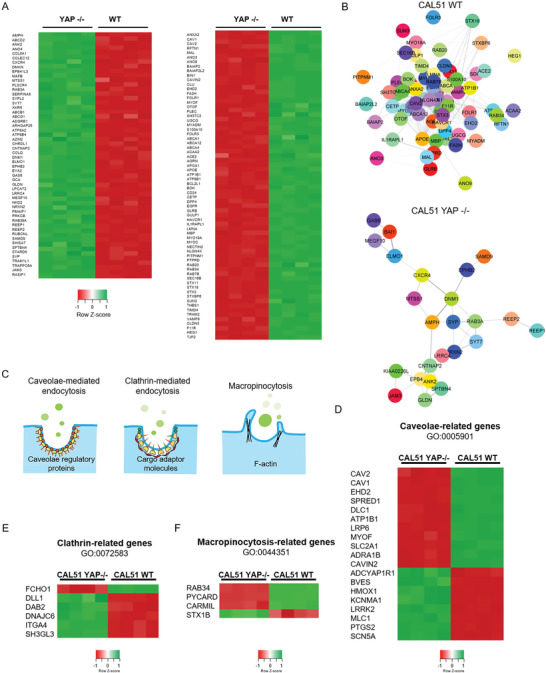
YAP depletion alters the expression of genes related to membrane organization and endocytosis pathways. A) Heatmap of the relative expression of significantly regulated genes associated with the membrane organization network in YAP ‐/‐ and WT CAL51 cells. *n* = 4 (P adj < 0.05, log2Fc > ǀ2ǀ). B) STRING PPI network of differently expressed proteins involved in membrane organization in WT (top) and YAP ‐/‐ CAL51 (bottom) cells obtained from Cytoscape (P adj < 0.05, log2Fc > ǀ2ǀ, confidence cutoff 0.4). C) Graphical representation of the main mechanisms of endocytosis, i.e., caveolae‐related endocytosis, clathrin‐related endocytosis, and macropinocytosis, investigated in the present study. D) Heatmap of genes involved in endocytosis pathways for caveolae‐related genes. E) heatmap of genes involved in clathrin‐mediated endocytosis pathways. F). Heatmap of genes involved in macropinocytosis (P adj < 0.05, log2Fc > ǀ1ǀ).

In line with our findings regarding cell membrane organization (Figures 1H–M), we detected the presence of amphiphysin I (*AMPH1*) among the genes being affected the most by YAP depletion. *AMPH1* encodes for a protein that senses and generates membrane curvatures, is implicated in clathrin‐mediated endocytosis,^[^ ^41^ ^]^ and was significantly upregulated in CAL51 YAP ‐/‐ cells (log2Fc 7.69, *P* < 0.05, **Figure** **2**A). Together with *AMPH1*, several genes contributing to plasma membrane assembly that were overexpressed in WT CAL51 may explain the differences in cell membrane organization (Figure 2A). These include *RFTN1* (log2Fc 2.79), *EHD2* (log2Fc 2.4), *MYOC* (log2Fc 5.83) and *PITPNM1* (log2Fc 2.86).^[^ ^42^, ^43^, ^44^, ^45^ ^]^

GO enrichment analysis identified several molecular functions being differentially regulated that were connected to plasma membrane network components, with high scores for lipid raft organization, localization, and assembly (see Figure S5a, Supporting Information). Interestingly, lipid rafts, responsible for membrane heterogeneity, strongly impacts the stability and functionality of the plasma membrane at the nanoscale, thus directly supporting high sub‐compartmentalization and intrinsic organization.^[^ ^46^ ^]^ In addition, STRING PPI analysis yielded a highly clustered network containing 68 nodes and 384 edges for WT CAL51, while 54 nodes and 39 edges were identified for YAP‐depleted cells (Figure 2B). WT CAL51 cells showed a significantly higher number of interactors and interconnections between the key elements of the membrane organization network compared to YAP ‐/‐ cells, possibly indicating a higher degree of membrane complexity. The cluster coefficients for both conditions were quite similar (0.394 for WT and 0.5 for YAP ‐/‐). More importantly, RNA‐seq analysis revealed the differential expression of genes encoding proteins involved in endocytosis (Figure 2C; see Figure S5b, Supporting Information), a process that appears to be dysregulated in tumors.^[^ ^47^ ^]^ Therefore, the RNA‐seq analysis was extended to examine the expression of transcripts involved in the trafficking of intracellular organelles and endocytic pathways in YAP ‐/‐ cells compared to CAL51 WT cells. This comparison led to the identification of differentially expressed genes involved in caveolae‐mediated endocytosis (Figure 2D), clathrin‐mediated endocytosis (Figure 2E), and macropinocytosis (Figure 2F). While the number of differentially regulated genes in the caveolae‐mediated pathway was similar between WT and YAP ‐/‐ cells (11 in WT and 8 in YAP ‐/‐, although YAP ‐/‐ cells displayed overall higher upregulation; see Figure S5b, Supporting Information), clathrin‐related and macropinocytosis‐related genes showed the most striking difference, with more genes of the clathrin‐mediated pathway upregulated in YAP ‐/‐ cells (5 genes in YAP ‐/‐ vs 1 gene in WT) and more genes of the macropinocytosis‐mediated pathway upregulated in WT cells (1 gene in YAP ‐/‐ vs 3 genes in WT).

To further corroborate these findings, we performed a GO enrichment analysis on the genes involved in endocytosis that were differentially expressed in WT and YAP ‐/‐ cells. The analysis revealed significant differences between WT and YAP ‐/‐ cells in annotations for the negative and positive regulators of endocytosis (see Figures S6 and S7, Supporting Information). For the negative regulators (GO:0045806), WT cells showed high scores for the regulation of endocytic vesicles (*P* = 0.001) and early endosomes (*P* = 0.002) compared to YAP ‐/‐ cells (*P* = 0.13 for endocytic vesicles, *P* = 0.03 for early endosomes), whereas YAP ‐/‐ cells yielded high scores for endosomal recycling genes (*P* = 0.014). These findings indicate that YAP depletion in triple‐negative breast cancer cells lead to the downregulation of transcripts that encode proteins responsible for inhibiting the endocytic pathways (see Figure S6, Supporting Information). The positive regulators (GO:0045807), on the other hand, were more abundant in the absence of YAP: relative to WT CAL51, YAP ‐/‐ cells showed significant scores for the terms positive regulators of the endocytic vesicle (*P* = 3.11×10^−5^ in YAP ‐/‐ vs *P* = 0.006 in WT) and cytoplasmic vesicle membrane (*P* = 0.000476 in YAP ‐/‐ vs *P* = 0.02 in WT), while CAL51 WT had high scores for collagen‐containing extracellular matrix (*P* = 7.56×10^−5^) and secretory granule lumen (*P* = 0.00035; see Figure S7, Supporting Information). Taken together, these results indicate that YAP depletion in CAL51 TNBC cells determines an alteration in the genes involved in endocytosis.

### 2.3 YAP Regulates the Entry of Nanoparticles in CAL51 TNBC Cells

**Figure 3:**
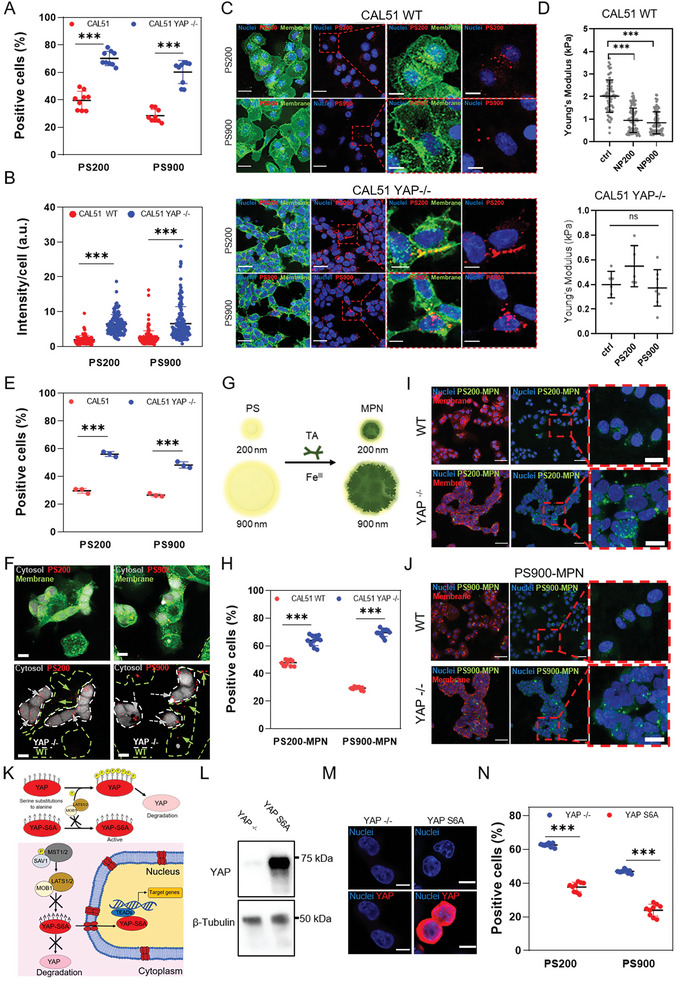
YAP knockout promotes nanoparticle binding and internalization in CAL51 TNBC cells. A) 4‐hour cellular uptake of PS200 and PS900 in WT (red) and YAP ‐/‐ CAL51 (blue). Statistical analysis was performed using the two‐way ANOVA followed by Sidak's multiple comparisons test. *n* = 3; ^***^ *p* < 0.001. B) Nanoparticle intensity per cell after 4 h of incubation of CAL51 WT and YAP ‐/‐ cells with PS200 and PS900. Statistical analysis was performed using the two‐way ANOVA followed by Sidak's multiple comparisons test. *n* = 3; ^***^ indicates *p* < 0.001. *n* > 100 cells. C) Confocal images of WT (top) and YAP ‐/‐ (bottom) CAL51 cells after 4 h of incubation with PS200 and PS900. Cells were stained with WGA‐488 (green) and/or DAPI (blue). Magnified images are displayed inside the red dashed line boxes for each cell and particle type. Scale bar: 25 and 10 µm. D) Young's modulus analysis of WT (top) and YAP ‐/‐ (bottom) cells after 4 h of incubation with PS200 and PS900, as measured by atomic force microscopy (AFM). Statistical analysis was performed using the Kruskal–Wallis one‐way ANOVA followed by Dunn's multiple comparisons test. WT CAL51: *n* = 80; YAP ‐/‐ CAL51: *n* = 10; ^***^ *p* < 0.001; ns, non‐significant. E) 4‐hour cellular uptake of PS200 and PS900 in a co‐culture of WT (red) and YAP ‐/‐ (blue) CAL51. Statistical analysis was performed using the two‐way ANOVA followed by Sidak's multiple comparisons test. *n* = 3; ^***^ *p* < 0.001. F) Confocal images of WT and YAP ‐/‐ cells co‐culture in the presence of PS200 and PS900 for 4 h. White dashed line indicates CAL51 YAP ‐/‐ cells. Grey arrows indicate particles co‐localized with YAP ‐/‐ cells (encircled by white dashed lines), while green arrows indicate particles in contact with WT cells (encircled by green dashed lines). CAL51 YAP ‐/‐ cells are stained with 7‐amino‐4‐chloromethylcoumarin (grey) and whole cell population with WGA‐488 (green). Scale bar: 10 µm. G) The surface properties of PS200 and PS900 were modified by MPN coating, using tannic acid and FeCl_3_. H) 4‐hour cellular uptake of PS200‐MPN and PS900‐MPN for WT (red) and YAP ‐/‐ (blue) CAL51. Statistical analysis was performed using the two‐way ANOVA followed by Sidak's multiple comparisons test. *n* = 3; ^***^ *p* < 0.001. I,J) Confocal images of WT (top) and YAP ‐/‐ (bottom) CAL51 cells after 4 h of incubation with PS200 (I) and PS900 (J). Cells are stained with WGA‐647 (red) and DAPI (blue). The particles are displayed in green. Magnified images are displayed inside red dashed line boxes for each cell and particle type. Scale bar: 50 and 10 µm. K) Schematic representation of the phosphorylation‐mediated repression of YAP translocation to the nucleus by kinases involved in different pathways, mainly Hippo pathway (LATS1/2 kinases and scaffolding protein MOB1). Due to substitutions of serine residues with alanine residues in six different positions (S61A, S109A, S127A, S128A, S131A, S136A, S164A, and S381A), YAP‐S6A cannot be phosphorylated by upstream kinases, thus is constitutively active in the cell nucleus. L) Western blot analysis of the levels of YAP protein in CAL51 YAP ‐/‐ and in cells transfected with a plasmid carrying a copy of the YAPS6A gene. β‐tubulin was used for protein loading normalization. M) Confocal images of YAP ‐/‐ (CTRL) and YAPS6A CAL51. Cells were stained with DAPI (blue) and YAP (AF555, red). Scale bar: 10 µm. N) 4‐hour cellular uptake of PS200 and PS900 in YAP ‐/‐ (blue) and YAPS6A (red) CAL51. Statistical analysis was performed using the two‐way ANOVA followed by Sidak's multiple comparisons test. *n* = 3; ^***^ *p* < 0.001.

Encouraged by these findings, we hypothesized that YAP might play a role in the internalization of nanoparticles and nanodrugs. We figured this might explain how Hippo pathway effector promotes TNBC resistance to chemotherapy.^[^ ^48^ ^]^ To test this hypothesis, we treated WT and CAL51 YAP ‐/‐ cells with inert polystyrene (PS) nanoparticles (NPs), which have been widely used in bio‐nano interaction studies due to their tunable size and ease of functionalization.^[^ ^49^, ^50^ ^]^ Carboxylated PS nanoparticles with 200 and 900 nm diameter (PS200 and PS900) were first labeled with carboxytetramethylrhodamine using EDC chemistry and characterized via transmission electron microscopy (TEM), spectrofluorometer, and dynamic light scattering (DLS) (see Figure S8a–e, Supporting Information). Next, WT and YAP ‐/‐ CAL51 cells were incubated with PS200 and PS900, and nanoparticle binding to the cells evaluated using flow cytometry and confocal microscopy. Our flow cytometry results showed that already after 4 h incubation, PS nanoparticles could be found preferentially bound to YAP ‐/‐ CAL51 cells as compared to their WT counterparts (**Figure** **3**A; see Figures S9 and S10a, Supporting Information). This effect was independent of the nanoparticle size and could be further confirmed by confocal analysis. Through this analysis, we – in fact – showed that a higher number of nanoparticles co‐localized with the membrane of YAP ‐/‐ compared to WT cells (Figure 3B,C; see Figure S10b, Supporting Information). Interestingly, CPEM analysis allowed us to visualize the detailed morphology of the nanoparticles in contact with CAL51 cells, further corroborating these findings. In WT cells, NPs were bound to the external face of the membrane but not yet internalized after 4 h (see Figure S11a, Supporting Information). They in fact appeared bright on SEM and AFM imaging in CAL51 WT and showed limited co‐localization with the cell membrane (see the height profile of the area at the nanoparticle‐membrane binding site in Figure S11b, Supporting Information). On the contrary, at the same time‐point NPs were already surrounded by an organic coating attributable to plasma membrane in cells in which YAP had been depleted (see Figure S11c, Supporting Information). This result indicated that the nanoparticles were embedded in the cell membrane, as revealed by the reduction of the height profile at the nanoparticle‐membrane binding site (see Figure S11d, Supporting Information).

We next focused on investigating whether the striking difference in particle interaction with cell membrane could be due to the changes in membrane curvature, actin dynamics, and cell mechanosensing as induced by YAP activity.^[^ ^18^, ^51^ ^]^ Hence, we reduced the nanoparticle dosage and increased the incubation time, and found enhanced nanoparticle internalization over time in YAP ‐/‐ CAL51, regardless of dose and time (see Figure S12a, Supporting Information). No significant change was observed in YAP localization in WT cells after nanoparticle binding, suggesting no direct impact on intracellular protein shuttling (see Figure S12b, Supporting Information). However, a marked decrease in membrane stiffness was noted in WT CAL51 after 4 h of incubation with nanoparticles, but no such change was seen in YAP‐depleted cells (Figure 3D). Although the effect of nanoparticles on the cell membrane properties is debated, with responses possibly dependent not only on the nanoparticle size but also their composition,^[^ ^52^ ^]^ our data suggest that the reduction in membrane rigidity in WT CAL51 did not enhance nanoparticle internalization over time, as YAP ‐/‐ cells exhibited higher binding and internalization in all conditions tested. This was likely due to a stronger impact of YAP depletion on cell membrane stiffness than the physical effects of nanomaterial interactions alone. Furthermore, z‐stack confocal images and TEM micrographs confirmed the internalization of particles in both WT and YAP ‐/‐ cells (see Figure S13a,b, Supporting Information), although a higher number of particles was found to bind and co‐localize with the cell membrane in the absence of YAP.

Next, we treated WT and CAL51 YAP ‐/‐ cells with inhibitors selective for each type of endocytosis to study the impact of YAP on the route of nanoparticle internalization in CAL51 TNBC cells. In particular, the cells were pre‐treated for 2 h with cytochalasin D, chlorpromazine, and nystatin to inhibit macropinocytosis, caveolin‐mediated, and clathrin‐mediated endocytosis, respectively.^[^ ^33^ ^]^ The cells were then incubated with PS200 and PS900 for 4 h. The live/dead assay was used to confirm no significant effect on cell viability under the chosen treatment conditions (see Figure S14, Supporting Information). The obtained results showed that PS200 primarily entered the cells through clathrin‐mediated endocytosis, while PS900 primarily did so through macropinocytosis (see Figure S15a,b, Supporting Information), which was in line with previous findings for similar‐sized nanoparticles.^[^ ^53^ ^]^ After internalization, the particles followed classical endocytic pathways and accumulated in lysosomes within 8 h of incubation (see Figure S15c, Supporting Information). These endocytic processes were not affected by the absence of YAP, suggesting that the protein per se does not impact endocytosis pathways but rather affects the dynamics of cell‐nanoparticle association and internalization.

To evaluate the potential of manipulating tumor cell mechanosensing to enhance nanoparticle delivery in a heterogeneous and complex milieu, we co‐cultured WT CAL51 cells with YAP ‐/‐ cells in a 1:1 ratio. The latter had been previously labeled with 7‐amino‐4‐chloromethylcoumarin (CellTracker). The co‐culture was incubated with either PS200 or PS900 for 4 h. In line with our cell morphology data (Figure 1), YAP ‐/‐ cells had a lower total surface area compared to the parental line due to their tendency to spread less on the substrate (see Figure S16a,b, Supporting Information). Interestingly, despite the lower area exposed, YAP‐depleted cells bound and internalized a significantly higher number of nanoparticles, as revealed by flow cytometry and confocal analysis (Figure 3E,F; see Figure S16c, Supporting Information). Indeed, z‐projection confocal images showed consistent preferential co‐localization of nanoparticles within the membrane of YAP ‐/‐ CAL51 cells (see Figure S17a,b, Supporting Information). This result contradicts previous studies linking a higher cell surface area with a higher internalization rate,^[^ ^52^, ^54^ ^]^ and suggests that YAP mechanosensing and cell mechanics outplay cell surface area in nanoparticle binding.

**Figure 4:**
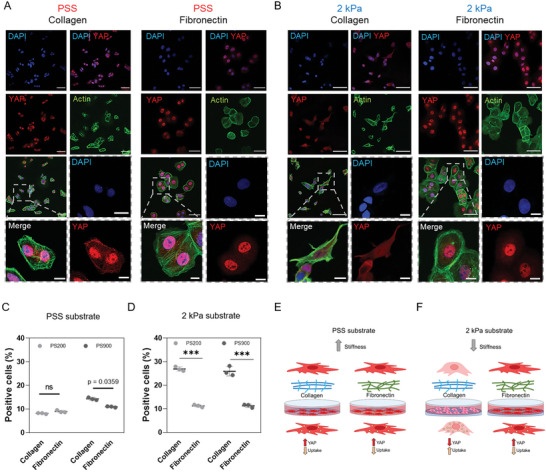
Substrate mechanics impairs nanoparticle uptake through YAP. A) Confocal images of CAL51 WT cells grown on a stiff polystyrene substrate coated with collagen (left) or fibronectin (right). Cells were stained with DAPI (blue), Pha‐488 (green), and YAP (AF555, red). Magnified images are displayed inside gray dashed line boxes. Scale bar: 50 and 10 µm. B) Confocal images of WT CAL51 cells grown on a 2 kPa soft substrate coated with collagen (left) or fibronectin (right). Cells were stained with DAPI (blue), Pha‐488 (green), and YAP (AF555, red). Magnified images are displayed inside gray dashed line boxes. Scale bar: 50 and 10 µm. C) 4‐hour cellular uptake of PS200 (light gray) and PS900 (dark gray) in WT cells grown on a stiff polystyrene substrate coated with collagen or fibronectin. Statistical analysis was performed using the two‐way ANOVA followed by Sidak's multiple comparisons test. *n* = 3; ns, non‐significant. D) 4‐hour cellular uptake of PS200 (light gray) and PS900 (dark gray) in WT CAL51 grown on a 2 kPa soft substrate coated with collagen or fibronectin. Statistical analysis was performed using the two‐way ANOVA followed by Sidak's multiple comparisons test. *n* = 3; ^***^ *p* < 0.001. E) Schematic representation of the mechanism proposed for the differences found in nanoparticle uptake in WT CAL51 cells grown on polystyrene substrates. The cells are well‐spread and attached to the surface, with high YAP nuclear localization. F) Schematic representation of the mechanism proposed for the differences found in nanoparticle uptake in WT CAL51 cells grown on soft substrates. When cells are grown on a soft substrate with a molecular‐mechanical inert coating such as collagen, YAP shuttles out of the nucleus in an inactive state, and cells appear round and poorly spread. This decrease in YAP activity leads to a significant increase in nanoparticle uptake. Conversely, fibronectin coating outplays soft stiffness substrates, activates YAP and restores the mechanical properties. Nanoparticle uptake is reduced similar to what happens on stiff polystyrene.

Next, we tested whether the internalization of nanoparticles was affected by their surface coating and charge. These parameters are crucial in bio‐nano interaction studies and have been extensively studied to optimize nanoparticle design for efficient targeting or escape from specific cell types for improved therapy delivery.^[^ ^55^ ^]^ To determine the impact of nanoparticle surface properties on the differential internalization rate seen in CAL51 cells with or without YAP, PS nanoparticles were coated with the metal phenolic network (MPN) to alter their physicochemical properties, such as surface charge and free energy.^[^ ^56^ ^]^ Briefly, the MPN coating was applied to the surface of 5‐((5‐Aminopentyl)thioureidyl)fluorescein‐labeled PS200 and PS900 nanoparticles by the assembly of tannic acid (TA) and FeCl_3_ according to a previously described protocol.^[^ ^50^ ^]^ The application of the MPN coating formed PS200‐MPN and PS900‐MPN (Figure 3G; see Figure S18a, Supporting Information), and the success of the coating was confirmed using a spectrophotometer and dynamic light scattering (DLS) analysis (see Figure S18b–e, Supporting Information). To examine cell‐nanoparticle interactions, WT and YAP ‐/‐ CAL51 cells were incubated with PS200‐MPN and PS900‐MPN for 4 h. Flow cytometry and confocal analysis revealed higher binding and internalization in YAP ‐/‐ cells compared to WT cells, supporting the previous results observed using non‐coated PS200 and PS900 nanoparticles (**Figure** **4**H–J; see Figure S19a,b, Supporting Information). Our findings indicate that despite coating the particles with MPN, the binding and internalization trends remain consistent with those observed for the uncoated particles and that the influence of cell mechanobiology, specifically the protein YAP, supersedes the properties of the materials in cell–nanoparticle interactions process.

Analog internalization pattern between the two cell lines was observed by testing fluid‐phase endocytosis and macropinocytosis with fluorescent dextran (Dx‐FITC) and pHrodo‐zymosan respectively (see Figure S20a,b, Supporting Information), and in free‐serum conditions where protein corona effect is ruled out (see Figure S21a,b, Supporting Information).

Compellingly, similar results were obtained in tumorigenic HEK293 WT or YAP‐depleted cells (see Figures S22 and S23, Supporting Information), while no differences in nanoparticles binding and internalization was observed in a non‐cancer cell line model of iPSC‐induced fibroblasts in presence or absence of YAP, indicating the selectivity of YAP role for regulating this process in carcinogenic cells (see Figure S24, Supporting Information).

The transcriptional activity of YAP requires its shuttling to the cell nucleus, where it acts as co‐activator by interacting with context‐specific transcription factors.^[^ ^57^ ^]^ To determine the role of YAP in repressing nanoparticle uptake in TNBC cells, YAP ‐/‐ cells were transfected with a YAP hyperactive mutant (YAP‐S6A). The mutant form of YAP carries a set of mutations in its sequence that converts the residues S61, S109, S127, S128, S131, S136, S164, and S381 into alanine residues. These mutations render the protein non‐phosphorylatable and, consequently, resistant to inactivation and/or degradation. The shuttling of the YAP‐S6A mutant protein to the nucleus is not inhibited via phosphorylation by the upstream Hippo pathway effectors LATS1/2 and MOB1 (Figure 3K). A mock vector was used as control. The transfection was first verified by quantifying the expression of YAP protein using western blot and confocal imaging (Figure 3L,M). The YAP‐S6A‐ or mock‐transduced CAL51 cells were then incubated with PS200 and PS900 nanoparticles. In line with the hypothesis that YAP presence in the nucleus represses nanoparticle uptake, YAP‐S6A cells exhibited reduced nanoparticle uptake after 4 h of incubation compared to YAP ‐/‐ cells transfected with a mock vector (Figure 3N). Interestingly, transfecting YAP‐S6A in WT cells (YAP +/+ CAL51; see Figure S25a, Supporting Information) did not induce any significant change in nanoparticle uptake (see Figure S25b–f, Supporting Information). This result could be explained by the fact that parental CAL51 cells already expressed a very high basal level of YAP, and the expression of a hyperactive mutant YAP‐S6A did not induce any noticeable change in cell morphology (see Figure S25d, Supporting Information), adhesion (see Figure S25e, Supporting Information), or transcription. RT‐qPCR further corroborated this hypothesis, showing no change in mRNA levels of CYR61 and CTGF, the two main transcriptional targets of YAP, in YAP +/+ CAL51 compared to WT cells (see Figure S25f, Supporting Information).

These findings indicate that YAP activity affects cell‐nanoparticle interactions and point at YAP as potential regulator of nanoparticles internalization.

### 2.4 Substrate Stiffness Hinders Nanoparticle Uptake through YAP

The stiffness of the tumor stroma has been reported to impact YAP intracellular localization and transcriptional activity,^[^ ^25^ ^]^ which correlates with the ability of cancer cells to metastasize, leading to treatment resistance and poor prognosis.^[^ ^58^ ^]^ YAP intracellular shuttling and transcriptional activity are controlled by the mechanical properties of the surrounding microenvironment, with cells grown on soft substrates (*E*<5 kPa) exhibiting cytosolic YAP localization while the protein moves to the nucleus on stiffer substrates (*E*>10 kPa).^[^ ^59^ ^]^ Additionally, the sensitivity of YAP to ECM components such as collagen and fibronectin has been previously documented,^[^ ^9^ ^]^ with fibronectin accumulation during ECM remodeling triggering YAP nuclear shuttling, independently of substrate stiffness.^[^ ^18^, ^60^ ^]^ To investigate the influence of substrate stiffness and ECM composition on nanoparticle internalization in TNBC CAL51 cells through YAP, WT and YAP ‐/‐ cells were cultured on tissue culture polystyrene (TCPS, Young's modulus in GPa range) or soft surfaces (PDMS) with a defined Young's modulus of 2 kPa coated with either collagen or fibronectin. Confocal microscopy was used to quantify YAP subcellular localization in TNBC cells in response to substrate stiffness or ECM composition. First, we found that in the presence of collagen, the soft substrate hindered YAP nuclear localization, while the presence of fibronectin reversed this effect and restored YAP shuttling to the nucleus of cells grown on a soft substrate (**Figure** **4**A,B). This indicated that the biochemical cues from fibronectin were able to overcome the effects of the mechanical properties of the substrate under the given experimental conditions. We then investigated the relationship among substrate stiffness, YAP activity and nanoparticle uptake in TNBC cells by incubating cells cultured on soft or stiff substrates with PS200 and PS900 nanoparticles for 4 h. Our results showed that CAL51 WT cells grown on 2 kPa soft substrate coated with collagen (low YAP activity) exhibited increased nanoparticle uptake, whereas this phenomenon was not observed on stiff PSS or 2 kPa substrates coated with fibronectin (high YAP activity, Figure 4C–F). Conversely, YAP ‐/‐ CAL51 cells showed no change in nanoparticle internalization on either PSS or 2 kPa substrates coated with collagen or fibronectin (see Figure S26, Supporting Information). These results suggest that YAP mechanical displacement from the nucleus could be an effective way to augment nanoparticle uptake in TNBC cells.

### 2.5 YAP Promotes ECM Network Deposition and Affects Cell‐Nanoparticle Interactions in a 3D In Vitro Model of TNBC

**Figure 5:**
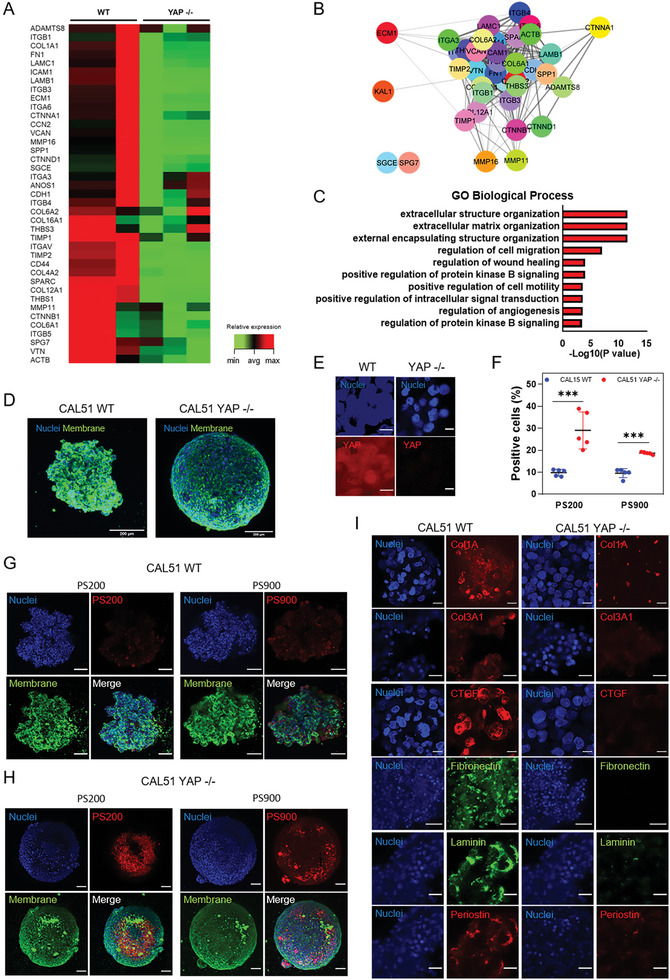
YAP knockdown affects nanoparticle binding and ECM deposition in 3D CAL51 spheroids. A) Heatmap representing the changes in expression for ECM and cell adhesion molecules in YAP ‐/‐ compared to WT CAL51 cells, as obtained from RT^2^‐profiler PCR array analysis (P adj < 0.05, fold change 2). B) STRING PPI network of differently expressed ECM proteins in CAL51 WT obtained from Cytoscape (P adj < 0.05, log2Fc > ǀ2ǀ, confidence cutoff 0.4). C) Bar plot representation of common enriched biological processes and pathways related to ECM network from the ENRICHR database, showing the most significantly upregulated genes in WT compared to YAP ‐/‐ CAL51 cells (P adj < 0.05, log2Fc > ǀ2ǀ). D) Z‐projection images of WT (left) and YAP ‐/‐ CAL51 (right) spheroids after 5 days of culture. Cells are stained with WGA‐488 (green) and DAPI (blue). Scale bar: 200 µm. E) Confocal images of the spheroids derived from WT and YAP ‐/‐ cells. Cells were stained with DAPI (blue) and YAP (AF555, red). Scale bar: 10 µm. F) 4‐hour cellular uptake of PS200 and PS900 in WT (red) and YAP ‐/‐ (blue) CAL51 spheroids. Statistical analysis was performed using the two‐way ANOVA followed by Sidak's multiple comparisons test. *n* = 5; ^***^ *p* < 0.001. G) Confocal images of the spheroids derived from WT CAL51 cells incubated with PS200 (left) and PS900 (right) for 4 h. H) Representative confocal images of the spheroids derived from YAP‐/‐ CAL51 cells incubated with PS200 (left) and PS900 (right) for 4 h. Cells were stained with WGA‐488 (green) and DAPI (blue). Nanoparticles are shown in red. Scale bar: 100 µm. I) Representative confocal images of the indicated ECM components for the spheroids derived from WT (left) and YAP ‐/‐ (right) CAL51 cells. Collagen type 1 alpha (Col1A), collagen type III alpha 1 (Col3A1), connective tissue growth factor (CTGF), and periostin are stained with 2nd antibody labeled with AF‐555 (red); fibronectin and laminin are stained with II‐antibody labeled with AF‐488 (green). Scale bar: 25 µm.

The efficiency of endocytosis is tightly linked to the composition of the ECM that surrounds cells. We hypothesized that the increase in nanoparticle uptake measured in YAP ‐/‐ cells could be explained by a less structured ECM in these cells. We first performed an RT^2^‐profiler PCR array and found that many genes related to ECM‐cell adhesion were significantly upregulated in WT cells compared to YAP ‐/‐ cells (**Figure** **5**A; see Figure S27a,b, Supporting Information). Some of these genes are known to be directly regulated by YAP transcriptional activity, such as CTGF (CCN2). To get a deeper insight into how YAP regulates the ECM landscape, we explored network connectivity and ontological interconnections between ECM components identified in both WT and YAP ‐/‐ cells using the STRING protein‐protein interaction (PPI) database with a score threshold of 0.4.^[^ ^61^ ^]^ The STRING PPI analysis yielded a highly clustered network containing 39 nodes and 336 edges and with a clustering coefficient of 0.74 for WT CAL51, indicating a significant high number of interactions (Figure 5B). On the opposite, the analysis of YAP ‐/‐ CAL51 cells showed a network with fewer connections, containing 38 nodes and 165 edges, and with a lower clustering coefficient of 0.49 (see Figure S27c, Supporting Information). As expected, the GO enrichment analysis revealed significant differences in the molecular function of the ECM network between WT and YAP ‐/‐ cells. While the former cells had high scores for annotations related to ECM and extracellular structure organization (*P* = 2.46×10^−12^) (Figure 5C), the latter displayed low scores for annotations related to ECM (*P* = 0.0043) and extracellular structure organization (*P* = 0.0078) (see Figure S27d, Supporting Information). Interestingly, the analysis also revealed that YAP depletion led to the dysregulation of several classes of ECM transcripts associated with cancer or known to promote tumor growth (see Figure S28a,b, Supporting Information).^[^ ^62^ ^]^

Next, we aimed to validate the hypothesis that reduced ECM deposition in YAP ‐/‐ cells was responsible for increased nanoparticle uptake. We established a 3D cell culture system, which resembles the complex and heterogeneous tumor microenvironment more closely than a 2D monolayer.^[^ ^63^, ^64^ ^]^ We generated 3D spheroids of both WT and YAP ‐/‐ CAL51cells and investigated nanoparticle uptake using this experimental model. Briefly, the cells were seeded onto round‐bottom ultra‐low attachment plates and spun to promote their aggregation. As expected, after 5 days of culture, the spheroids obtained from WT or YAP ‐/‐ cells showed distinct morphologies similar to what previously described (Figure 5D; see Figure S29a, Supporting Information).^[^ ^18^ ^]^

Then, we investigated YAP expression in WT spheroids and found that YAP was evenly distributed in both the cytoplasm and nucleus of the cells (Figure 5E). The live/dead assay confirmed that the cells were viable, with no detectable sign of cell death in either WT or YAP ‐/‐ spheroids (see Figure S29b, Supporting Information). We then incubated WT and YAP ‐/‐ spheroids with PS200 and PS900 nanoparticles and quantified nanoparticle binding using flow cytometry and confocal imaging. After 4 h of incubation, we found that YAP ‐/‐ CAL51 spheroids exhibited significantly higher nanoparticle binding than WT spheroids (Figure 5F–H). This result was also confirmed using different nanoparticle concentrations and incubation times (see Figure S29c,d, Supporting Information). Next, we stained WT and YAP ‐/‐ CAL51 spheroids with antibodies directed against relevant ECM proteins. The confocal microscopy analysis demonstrated that CAL51 WT cells produced a rich and multicomponent ECM composed of various proteins such as collagen, CTGF, fibronectin, laminin, and periostin, all contributing to the spheroid assembly. In contrast, YAP ‐/‐ depletion determined a stark reduction in the expression of the same ECM proteins (Figure 5I; see Figure S30, Supporting Information). Considering these results, targeting YAP may serve as a promising strategy for improving nanoparticle uptake into solid tumors by tuning cell membrane properties and decreasing ECM deposition.

### 2.6 YAP Targeting Improves Nanomedicine Delivery to TNBC Cells

**Figure 6:**
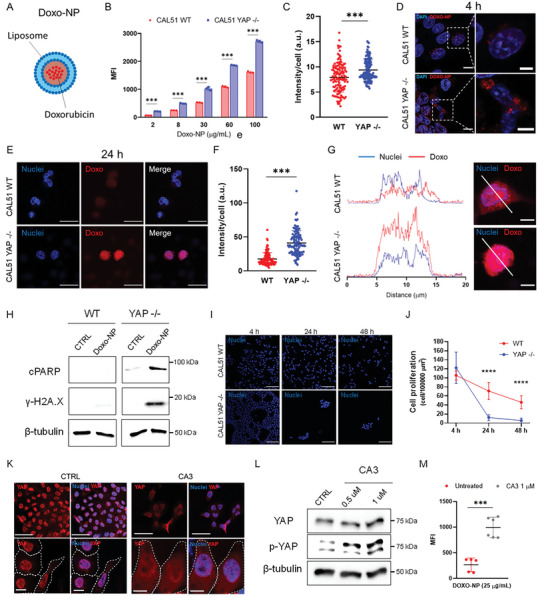
Pharmacological and genetic targeting of YAP increases the internalization of doxorubicin‐loaded liposomes and improves drug delivery in TNBC CAL51 cells. A) Graphical representation of doxorubicin‐loaded liposome (Doxo‐NP) formulation used for drug delivery. B) Median fluorescence intensity (MFI) of Doxo‐NP uptake in WT (red) and YAP ‐/‐ (blue) CAL51 as a function of nanoparticle concentration after 4‐hour incubation. Statistical analysis was performed using the two‐way ANOVA followed by Sidak's multiple comparisons test. *n* = 3; ^***^ *p* < 0.001. C) Nanoparticle intensity per cell after a 4‐hour incubation of WT and YAP ‐/‐ CAL51 cells with Doxo‐NP. Statistical analysis was performed using the unpaired *t*‐test with Welch's correction. *n* > 120; ^***^ *p* < 0.001. D) Confocal images of WT (top) and YAP ‐/‐ (bottom) CAL51 cells after 4‐hour incubation with Doxo‐NP. Cells were stained with DAPI (blue). Nanoparticles are displayed in red. Magnified images are shown inside white dashed line boxes. Scale bar: 10 and 5 µm. E) Representative confocal images of WT (top) and YAP ‐/‐ (bottom) CAL51 cells at 24 h after 4‐hour incubation with Doxo‐NP. Cells were stained with DAPI (blue). Doxorubicin is displayed in red. Scale bar: 20 µm. F) Dot plot representation of doxorubicin intensity per cell at 24 h after a 4‐hour incubation of WT and YAP ‐/‐ cells with Doxo‐NP. Statistical analysis was performed using the unpaired *t*‐test with Welch's correction. *n* > 100; ^***^ *p* < 0.001. G) A plot profile of doxorubicin intensity (red) co‐localized with the nucleus (blue, DAPI) of WT (top) and YAP ‐/‐ (bottom) CAL51 cells. On the right, confocal images show a detailed view of the region chosen for the intensity plots (white line) in WT (top) and YAP ‐/‐ (bottom) cells. Scale bar: 10 µm. H) Western blot showing the levels of cleaved PARP (cPARP) and histone H2AX (γ‐H2A.X) in WT (left) and YAP ‐/‐ (right) CAL51 cells untreated (CTRL) or treated with Doxo‐NP for 4 h and collected for the analysis 48 h post‐treatment. β‐tubulin was used for protein loading normalization. I) Representative confocal images of WT (top) and YAP ‐/‐ (bottom) CAL51cells after 4 h of incubation with Doxo‐NP and 24 and 48 h after treatment with the nanoparticles. Nuclei were stained with DAPI. Scale bar: 100 µm. J) Cell proliferation plot expressed as number of cells per surface area for WT (red line and circle) and YAP ‐/‐ (blue line and squares) CAL51cells at 0, 24, and 48 h after 4‐hour Doxo‐NP treatment. Statistical analysis was performed using the two‐way ANOVA followed by Sidak's multiple comparisons test. *n* = 3; ^***^ *p* < 0.001. K) Representative confocal images of untreated (CTRL, left) or CA3‐treated WT cells (1 µm CA3, left) for 12 h. Cells were stained with YAP (AF555, red) and the nuclei were counterstained with DAPI (blue) and. The cell perimeter and cell nuclei are highlighted with a dashed white line and a dashed blue line respectively, in magnified images (bottom). Scale bar: 50 and 10 µm. L) Western blot showing the levels of YAP and phospho‐YAP (p‐YAP) in WT CAL51 untreated (CTRL) or treated with 0.5 and 1 µm CA3 inhibitor for 12 h. β‐tubulin was used for protein loading normalization. M) MFI after a 4‐hour incubation of CAL51 WT cells with Doxo‐NP without treatment (red) or after treatment with 1 µm CA3 inhibitor for 12 h (grey). Statistical analysis was performed using the unpaired t‐test with Welch's correction. *n* = 5; ^***^ *p* < 0.001.

After demonstrating that YAP depletion can be leveraged to increase nanoparticle uptake in TNBC CAL51 cells, we next aimed to demonstrate the therapeutic benefits of combining nanoparticle treatment with YAP targeting. To assess the efficiency of drug delivery, we chose liposomes (**Figure** **6**A; see Figure S31a,b, Supporting Information), as they have a long history of success since Doxil,^[^ ^46^ ^]^ the first nano drug that reached the market, and have been recently used in nanoformulations to treat cancer and other diseases.^[^ ^65^, ^66^, ^67^ ^]^ We used a doxorubicin‐loaded liposomal formulation (Doxo‐NP) and evaluated its drug delivery efficiency in both WT and YAP ‐/‐ cells. Flow cytometry analysis showed significantly higher fluorescence intensity in YAP ‐/‐ compared to WT CAL51cells after 4‐hour incubation with different concentrations of Doxo‐NP (Figure 6B; see Figure S31c, Supporting Information). Confocal imaging confirmed a higher association of nanoparticles with YAP ‐/‐ cells (Figure 6C,D; see Figure S31d, Supporting Information). Results from the WT and YAP ‐/‐ CAL51 co‐culture and 3D spheroid experiments were also consistent (see Figures S31e and S32a,b, Supporting Information).

Next, we extended the treatment to 24 h and used confocal imaging to show that doxorubicin accumulated more in the nuclei of YAP ‐/‐ cells as compared to WT cells (Figure 6E–G). Additionally, we performed western blot analysis 48 h post‐treatment with antibodies directed against cPARP and γ‐H2AX and detected increased expression of both proteins in YAP ‐/‐ CAL51 cells following Doxo‐NP treatment. This effect was blunted in WT cells treated with the same NPs (Figure 6H). High toxicity was observed in YAP ‐/‐ cells at 24‐ and 48‐hours post‐treatment, with a considerably lower number of live cells per well compared to WT CAL51 (Figure 6I,J).

Finally, we investigated if the pharmacological inhibition of YAP could be exploited to enhance Doxo‐NP uptake in TNBC CAL51 cells. To this purpose, small molecule CA3 (1 µm) was used to inhibit YAP activity in breast tumor cells for 12 h and showed no significant toxicity (see Figure S33a, Supporting Information).^[^ ^68^ ^]^ Confocal images showed that CA3 treatment caused YAP to shuttle from the nucleus to the cytoplasm (Figure 6K; see Figure S34, Supporting Information), which was confirmed by the western blot analysis, revealing CA3‐induced phosphorylation of YAP (Figure 6L; see Figure S33b, Supporting Information). Importantly, treatment with CA3 followed by a 4‐hour incubation with Doxo‐NP significantly increased nanoparticle binding and internalization (Figure 6M; see Figure S33c, Supporting Information). It also increased the toxicity of the treatment with the nanoformulation with respect to the nanodrug alone, due to increased nanoparticle internalization and drug release (see Figure S33d, Supporting Information). Interestingly, the same effect of YAP inhibition by CA3, in terms of nanoparticle‐cell association, was observed on another TNBC cell line, MDA‐MB‐231 (see Figure S35a,b, Supporting Information).

In light of these results, the inhibition of YAP using suitable drugs may be a promising strategy to improve cancer cell toxicity when combined with nanomedicine for the treatment of TNBC.

## 3 Conclusion

Due to its pro‐tumorigenic role as an oncogene, YAP has been proposed as a target to halt cancer progression.^[^ ^69^ ^]^ In this study, we showed that YAP depletion in the TNBC cell line CAL51 significantly alters the mechanical properties, surface area and adhesion of the cells. Moreover, we demonstrated that YAP controls the genetic landscape of CAL51 cells by impacting the transcription of genes involved in membrane organization and endocytosis. RNA‐seq analysis revealed differential expression of several genes involved in cell‐substrate adhesion, actin‐membrane linkage, ECM production, contraction, membrane tension and organization in YAP ‐/‐ relative to CAL51 WT cells. Notably, we observed that YAP depletion determines the upregulation of several genes that positively regulate endocytosis and that may contribute to the internalization of nanoparticles. Recent studies have shown a connection between YAP and the endocytic machinery in the cytoplasm.^[^ ^70^ ^]^ These interactions have long been linked to protein turnover via a degradation pathway alternative to the Hippo pathway, LATS1/2 phosphorylation, and proteasome. However, as our understanding of the feedback between the plasma membrane domain and mechanosensing effectors advances, the cytoplasmic pool of YAP directly interacting with proteins of the membrane, vesicles, and organelles may uncover novel and unexpected functions related to the control of organelles trafficking. Our findings suggest that YAP may play a leading role in the regulation of endocytic processes; however, its role in the homeostasis of cytoplasmic vesicles and organelles remains unclear and warrants further investigation of the molecular pathways underlying these interactions.

Throughout the study, we found that YAP knockout led to changes in cell physical and biological properties, resulting in increased nanoparticle uptake that was exclusively linked to YAP activity, not the size or surface coating of the nanoparticles. Additionally, in a co‐culture system, where WT and YAP ‐/‐ CAL51 cells were seeded together, cells in which the Hippo effector had been genetically depleted showed a higher association rate with nanoparticles compared to WT cells, suggesting a possible mechanotargeting effect where cell mechanics plays a key role in bio‐nano interactions. While the substrate and cell mechanics are often intertwined in nanoparticle uptake processes,^[^ ^71^ ^]^ our study shows that YAP activity overrides substrate mechanics in controlling nanoparticle internalization. Indeed, irrespective of the Young's modulus of the surface where the cells were grown, YAP activity was the key determinant in nanoparticle uptake. Specifically, an increase in YAP activity through cell‐ECM interactions such as integrin‐fibronectin did not enhance nanoparticle uptake even on low‐stiffness substrates. Furthermore, in 3D spheroid cultures, the mechanical state of cell‐cell interaction greatly depended on ECM production and deposition, which could, in turn, impact nanoparticle association and penetration within the spheroids. As a result, nanoparticles tend to associate more with spheroids derived from YAP ‐/‐ than WT cells. These data indicate that the intracellular activity of YAP and related mechanosensing proteins, not substrate mechanics, play an active role in nanoparticle internalization.

Our findings show that cell mechanobiology contributes to cell‐nanoparticle interactions, and the functions of its principal pathways and effectors may improve nanoparticle delivery to cancer cells. The mechanobiology pathways identified here may be leveraged to ameliorate the design of nanoparticles, focusing on the development and characterization of nanomaterials that are able to interact with the cell membrane in more efficient ways. Although the role of YAP‐paralog protein TAZ (WWTR1) would be worth investigating in the context of breast cancer cell–nanoparticle interaction, in the present work, we have decided to focus exclusively on YAP due to our previous results that have shown that the latter exerts a stronger effect on cell adhesion to the extracellular matrix.^[^ ^18^ ^]^

However, considering that breast cancer, and cancer in general, is a complex disease, the role of YAP in TNBC and other cancers should be evaluated on a case‐by‐case basis. In our study, we propose that in cases where disease stratification is feasible, and YAP is found to be overexpressed, combining its inhibition with nanomedicine could offer a novel strategy to enhance therapeutic effectiveness. It is likely that other mechanosensing proteins play a significant role in regulating cell–nanoparticle interactions in different cell types or cancer cells, and further studies are necessary to elucidate these aspects.

From a clinical perspective, several mechanotherapeutic drugs are currently under evaluation in clinical trials and are expected to be used in combination with other therapies, such as chemotherapy, targeted therapies, and immunotherapies.^[^ ^72^ ^]^ The inhibition of YAP through appropriate drugs may represent a promising strategy when combined with nanomedicine administration to enhance the toxicity against cancer cells, similar to the approaches using Onivyde, a liposomal formulation of irinotecan used in combination with free leucovorin and 5‐fluorouracil for metastatic pancreatic cancer,^[^ ^73^ ^]^ and Apealea, a micellar formulation of paclitaxel administered in combination with carboplatin for the treatment of ovarian cancer.^[^ ^74^ ^]^ Noteworthy, recently, the co‐delivery of lipid nanoparticles (LNP) carrying FAK siRNA and CRISPR‐PD‐L1 has been demonstrated to be effective in reducing the extracellular matrix deposition and stiffness of cancer cells upon continuous administrations.^[^ ^75^ ^]^ This approach increases the delivery of LNPs, together with the transfection efficiency, and effectively induces the knock‐down of immune checkpoints.^[^ ^75^ ^]^

To summarize, in this study we have demonstrated that YAP co‐transcriptional activity hinders nanoparticle binding and internalization. As we explored along the paper, several reasons may account for this association and can be ascribed to the role of YAP in: i) directing the transcription of genes involved in cell adhesion and mechanosensing; ii) affecting the genetic landscape of endocytic pathways by transcriptionally suppressing proteins involved in endocytosis; iii) perturbing cell membrane tension and organization, thus promoting its deformation and facilitating the formation of endocytic vesicles; iv) producing an abundant and dense ECM network that may ultimately hamper nanoparticle diffusion.

In conclusion, by genetically, mechanically, or pharmacologically targeting YAP, we show that it is possible to increase nanoparticle association and internalization in TNBC cells, highlighting the role of mechanobiology in shaping the fate of bio‐nano interactions in cancer cells. We demonstrate that blocking YAP activity may be used to increase the delivery of nano drugs, paving the way for novel combinatorial therapies suited to tackle cancer tumorigenicity while simultaneously enhancing the delivery of anti‐cancer nanotherapeutics. This work opens up new avenues for selectively tuning cell‐nanoparticle interactions by targeting molecular processes that differentiate between cancer cells and their healthy counterpart, thus improving both the delivery and specificity of nanotherapy. To assess the pre‐clinical and clinical value of such nanotherapies, we propose an alternative fundamental mechanism for nanoparticle entry into the cells. A deeper understanding of the cell mechanobiology pathways in bio‐nano interactions and the search for new targets and drugs to modulate their functions could accelerate the development of advanced next‐generation nanotherapies that would address the challenges posed by nanomedicine concerning targeted and selective drug delivery.

## 4 Experimental Section

Detailed experimental methods can be found in *Supporting information*.

### Statistical Analysis

Results are based on at least three replicates, and the data are presented as the mean ± s.d. The calculations were performed using GraphPad Prism v. 6.0 (San Diego, USA). For single‐cell analysis, a minimum of 100 cells per sample were considered. Along the manuscript, the following statistical tests have been used: Unpaired *t*‐test with Welch's correction; Kruskal–Wallis one‐way ANOVA followed by post‐hoc Dunn's multiple comparisons test; two‐way ANOVA followed by Sidak's or Tukey's multiple comparisons test. The appropriate statistical test was applied to the data as indicated in the figure captions for each experiment. All data are presented as mean ± standard deviation (S.D.). A P < 0.05 was considered statistically significant as denoted with asterisks [(*) *p* ≤ 0.05, (**) *p* ≤ 0.01, (***) *p* ≤ 0.001].

## Conflict of Interest

The authors declare no conflict of interest.

## Author Contributions

M.C. and G.F. conceptualized the idea for the study. M.C., S.F., J.O.D.L.C., J.V., V.H., M.P., S.K., J.P., and D.D. designed the methodology. M.C. and S.F. performed investigation. M.C. visualized the idea for the study. M.C. and G.F. performed supervision and wrote the original draft. M.C., S.F., F.C., and G.F. wrote, reviewed, and edited the final manuscript.

## Supporting information

Supporting Information

Supporting Information

## Data Availability

The data that support the findings of this study are available from the corresponding author upon reasonable request.
