# Supplementary material for: YAP Signaling Regulates the Cellular Uptake and Therapeutic Effect of Nanoparticles
Source: Adv Sci (Weinh). 2023 Nov 9;11(2):2302965. doi: 10.1002/advs.202302965 (PMC10787066; doi:10.1002/advs.202302965)
Supplement: Supplementary file 1 — Supporting Information [file ADVS-11-2302965-s002.pdf]

ADVANCED SCIENCE
Open Access

Supporting Information

for *Adv. Sci.*, DOI 10.1002/advs.202302965

YAP Signaling Regulates the Cellular Uptake and Therapeutic Effect of Nanoparticles

Marco Cassani, Soraia Fernandes, Jorge Oliver-De La Cruz, Helena Durikova, Jan Vrbsky, Marek Patočka, Veronika Hegrova, Simon Klimovic, Jan Pribyl, Doriana Debellis, Petr Skladal, Francesca Cavalieri, Frank Caruso and Giancarlo Forte**

Supporting information

YAP signaling regulates the cellular uptake and therapeutic effect of nanoparticles

Marco Cassani[1,8], Soraia Fernandes[1], Jorge Oliver-De La Cruz[1,†], Helena Durikova[1], Jan Vrbsky[1], Marek Patočka[2,3], Veronika Hegrova[2], Simon Klimovic[4], Jan Pribyl[4], Doriana Debellis[5], Petr Skladal[4], Francesca Cavalieri[6,7,8], Frank Caruso[8], Giancarlo Forte[1, 9*]*

Affiliations

[1]International Clinical Research Center, St. Anne's University Hospital, Brno, Czech Republic. [2]NenoVision, Purkynova 649/127, Brno, 61200, Czech Republic. [3]Faculty of Mechanical Engineering, Brno University of Technology, Technicka 2896/2, Brno, 61669, Czech Republic. [4]Department of Bioanalytical Instrumentation, CEITEC Masaryk University, Brno, Czech Republic. [5]Electron Microscopy Facility, Fondazione Istituto Italiano Di Tecnologia, Via Morego 30, 16163, Genoa, Italy. [6]School of Science, RMIT University, Melbourne, Victoria, Australia. [7]Dipartimento di Scienze e Tecnologie Chimiche, Università di Roma "Tor Vergata", Via Della Ricerca Scientifica, Rome, Italy. [8]Department of Chemical Engineering, The University of Melbourne, Parkville, Victoria, Australia. [9]School of Cardiovascular and Metabolic Medicine & Sciences, King's College London, London WC2R 2LS, UK.

†Present address: Institute for Bioengineering of Catalonia (IBEC), The Barcelona Institute for Science and Technology (BIST), Barcelona, Spain.

*Corresponding author. Email: Email: giancarlo.forte@kcl.ac.uk (GF); marco.cassani@unimelb.edu.au (MC)

This PDF file includes:

- Supplementary Text
- Experimental Section
- Figs. S1 to S35
- Tables S1 to S4
- MIRIBEL Checklist (Tables S3 to S4)
- References (1 to 10)

Experimental Section

Materials. Polystyrene carboxylated nanoparticles 0.9 and 0.204 µm (W090CA and 83000520100290, respectively, Thermo fisher); µ-Dish 35 mm, 1.5 ESS (81291, Ibidi); CytoSoft Imaging, 24-well Plate, Elastic Modulus 2 kPa (5185-1EA, CellSystem); bovine plasma fibronectin (F1141, Merck); Collagen I, rat tail (A1048301, Thermo Fisher); Dulbecco's Modified Eagle Medium (DMEM, Merck); TrypLE™ Express (12604013, Thermo Fisher Scientific); Matrigel Growth Factor Reduced (356230, Corning); Essential 8™ Medium (A1517001, Thermo Fisher Scientific); Essential 6™ (A1516401, Thermo Fisher Scientific) Opti-Mem (31985062, Thermo Fisher); 6-well plate (30006, SPL Life Sciences); penicillin/streptomycin (97063-708, VWR); AggreWell™800 Microwell (34850, StemCell); Type B Gelatin (G6650, Merck); 12-well plate (30012, SPL Life Sciences), 24-well plate (30024, SPL Life Sciences); FibroGro Media (SCMF001, Merck); 96-well ultralow attachment plates (7007, Corning); µ-slide 8-well glass bottom dish (80807, Ibidi), Tissue culture dish 40 mm (93040, Ibiotech); Alexa Fluor™ 647 Phalloidin (A22287, Thermo Fisher); Alexa Fluor™ 488 Phalloidin (A12379, Thermo Fisher); Wheat Germ Agglutinin, Alexa Fluor™ 647 Conjugate (W32466, Thermo Fisher); Wheat Germ Agglutinin, Alexa Fluor™ 488 Conjugate (W11261, Thermo Fisher); Y-27626 (S1049, Selleck chemicals); pLX304 (Addgene plasmid # 25890; http://n2t.net/addgene:25890; RRID:Addgene_25890); YAP1 (S6A) - V5 in pLX304 (Addgene plasmid # 42562; http://n2t.net/ addgene: 42562; RRID:Addgene_42562); FuGENE HD (E2311, Promega); Lipofectamine 3000 (L3000001, Thermo Fisher); 10% Mini-Protean TGX Precast Protein gel (4561033, Bio-Rad); protease and phosphatase inhibitor cocktails (PPC1010, Merck); RIPA buffer (R0278, Merck); HRP-conjugated anti-rabbit and HRP-conjugated anti-mouse (RABHRP1 and RABHRP2, Merck); MOWIOL 4-88 Reagent (475904, Merck); LysoTracker Deep Red (L12492, Thermo Fisher); High Pure RNA Isolation Kit (11828665001, Roche); RT² Profiler™ PCR Array Human Extracellular Matrix & Adhesion Molecules (Ref. PAHS-013Z); LIVE/DEAD Viability/Cytotoxicity Kit (L3224, Thermo Fisher); PrestoBlue Cell Viability Reagent (A13262, Thermo Fisher); CellTrace CFSE Cell Proliferation Kit (C34554, Thermo Fisher); Dextran, Fluorescein, 10,000 MW (D1821, Thermo Fisher); pHrodo Green Zymosan Bioparticles (P35365, Thermo Fisher); Opti-Link Carboxylate-Modified Particles (83000520100290 and W090CA, Thermo Fisher); TAMRA cadaverine (92001, Biotium); Fluorescein Cadaverine (A10466, Thermo Fisher); Flot-A-Lyzer G2 dialysis device (300 kDa cutoff) (G235036, Merck); CellTracker Blue CMAC Dye (C2110, Thermo Fisher); N-Hydroxysuccinimide (804518, Merck); Chlorpromazine hydrochloride (sc-202537, Santa Cruz); Nystatin (sc-2122431, Santa Cruz); Cytochalasin D (C8273, Merck); CA3 (CIL56) (S8661, Selleckchem); EDC-hydrochloride (59002, VWR); sodium cacodylate trihydrate (20840, Merck); glutaraldehyde 25% (354400, Merck); 4′,6-Diamidine-2′-phenylindole dihydrochloride (10236276001, Merck); Dox-NP (300112, Avanti Polar Lipids); tannic acid (403040, Merck); Iron chloride tetrahydrate (380024, Merck); mouse anti-YAP (4912, Cell Signaling); mouse anti-Vinculin (V9131, Merck); anti-Col1A1 (91144S, Cell Signaling); anti-Col3A1 (PA5-27828, Thermo Fisher); anti-CTGF (Ab5097, Abcam), anti-Fibronectin (F3648, Merck); anti-Laminin (SAB4200719, Merck); anti-Periostin (49480, Santa Cruz); rabbit anti-YAP (14074, Cell Signaling); mouse anti-β-tubulin (T8328, Merck); mouse anti-vinculin (V9131, Merck); rabbit anti-pYAP S127 (D9W21, 13008, Cell Signaling); rabbit anti-pH2A.X (2577, Cell Signaling); rabbit anti-cPARP (5625S, Cell

Signaling). All antibodies used for western blotting were diluted at a ratio of 1:100. Antibodies for immunohistochemistry were used according to the specifications in Table 1 in the Supporting information.

Polystyrene nanoparticle fluorescent labeling. Polystyrene carboxylated nanoparticles with a size of 0.9 and 0.204 μm were labeled with TAMRA cadaverine or Fluorescein cadaverine. Briefly, the particles were resuspended in 5 mL MES buffer (50 mM, pH=6.04) at a final concentration of 20 mg/mL and incubated with 52 μM/mL EDC-hydrochloride and 5.2 μM/mL N-hydroxysuccinimide for 1 hour in an ice bath under magnetic stirring. Afterward, TAMRA cadaverine or fluorescein cadaverine was added, and the mixture was brought to a final concentration of 50 μM and left to react overnight (O.N.) at room temperature (RT). The day after, the particles were collected, centrifuged (5,000 rpm for 10 min for 0.9 μm particles, 12,000 rpm for 15 min for 0.204 μm particles), and washed five times with distilled water. Subsequently, the particles were dialyzed against distilled water for 72 hours using a Flot-A-Lyzer dialysis device (300 kDa cutoff).

Tannic Acid (TA)/Fe^{III} Coating on Nanoparticles. The MPN coating process was performed after fluorescein cadaverine functionalization of the nanoparticles; 250 μL of the particles were dispersed in an equal amount of distilled water, and 15 μL (PS 0.9 μm) or 5 μL (PS 0.204 μm) of FeCl3·6H2O (37 mM) added under sonication. After 1 min, 15 μL (PS 0.9 μm) or 5 μl (PS 0.204 μm) of tannic acid (TA) solution (24 mM) solution was added, and the mixture was left to react for 5 min under sonication. Afterward, the pH was raised by adding 500 μL of MOPS (20 mM, pH 7.4), and the suspension was vortexed for 5 min to ensure adequate MPN film formation and adherence. The particles were then centrifuged (5,000 rpm for 10 min for 0.9 μm particles, 12,000 rpm for 15 min for 0.204 μm particles) and washed five times with distilled water. Finally, the particles were resuspended in 250 μL of distilled water.

Generation of YAP mutant CAL51 lines. The YAP -/- CAL51 lines were generated using CRISPR/Cas9 technology as described previously.[1] Briefly, the process involved designing a guiding RNA to target exon 1 of the YAP1 gene, which is common in all nine YAP1 splicing variants. Two sets of complementary single-stranded DNA oligonucleotides (YAP1_R1: 50-CACCgtgcacgatctgatgcc-30, YAP1_R2: 50-AAACccgggcatcagatcgtgcac-30, YAP1_F1: 50-CACCGcatcagatcgtgcacgt-30, YAP1_F2 50-AAACcggacgtgcacgatctgatgC-30) were then cloned into pSpCas9(BB)-2A-GFP (PX458) and transfected into Cal51 cells using the FuGENE HD transfection reagent according to the manufacturer's protocol. The next day, green fluorescent protein (GFP)-positive cells were FACS-sorted (MoFlo Astrios, Beckman Coulter, California, USA) as single cells into a 96-well plate and clonally propagated. Genomic DNA was sequenced from both sides to map the size of the deletion (sequencing primers: 50-gattggacccatcgtttgcg-30, 50-gtcaagggagttggagggaaa-30, 50-gaagaaggagtcgggcagctt-30, 50-gagtggacgactccagttcc-30).

Cell Culture. Wild-type (WT) CAL51 (gift from Dr. L. Krejčí, Department of Biology, Masaryk University, Brno, Czech Republic), HEK293 (gift from Dr. V. Pekarik, Department of Physiology, Masaryk University, Brno, Czech Republic) and mutant cell line were cultured in Dulbecco's Modified Eagle Medium (DMEM) containing 10% Inactivated Fetal Bovine Serum (FBS), 1% Penicillin-Streptomycin (PS), and 1% Glutamine at 37 °C, 95% humidity, and 5% CO2. The cells were split every 2-3 days before reaching confluence, and only the CAL51 cells with a passage number < 20 and HEK293 cells with a passage number <10 were used for all the experiments.

Spheroids from CAL51 cells were generated by seeding 3,000 cells per well in a volume of 100 µL in 96-well ultralow attachment plates and aggregating them *via* centrifugation at 1000 rpm for 5 min. The spheroids were left to grow for 5 days with media replacement every 2 days. The cell proliferation assay was performed using the CellTrace CFSE Cell Proliferation Kit (C34554, Thermo Fisher). Cells were labeled with the dye according to the supplier's protocol and seeded onto a 24-well plate (30024, SPL Life Sciences) for 24 hours. Flow cytometry analysis was performed using unstained cells as a control. Cell viability was assessed with LIVE/DEAD Viability/Cytotoxicity Kit, for mammalian cells, or with PrestoBlue Cell Viability Reagent, according to manufacturer's instructions.

YAP knockout and isogenic H9 (WT) human embryonic stem cell lines (hESCs) were a kind gift of Miguel Ramalho-Santos and Han Qi.[2] The cells were maintained in an undifferentiated state by culturing them onto Matrigel Growth Factor Reduced in complete Essential 8 Medium with penicillin/streptomycin (1x). To differentiate them into fibroblasts, $1.2x10^6$ cells/well were seeded in AggreWell800 Microwell in Essential 6 supplemented with Y-27626. After 24h, ¾ parts of the media was changed to Essential 6 without Y-27626. After 48h, embryoid bodies were transferred to a 10 cm tissue culture dish coated with 0.2% Type B Gelatin in FibroGro Media containing 2% FBS and penicillin/streptomycin, and let to attach undisturbed. After 5 days, when the cells arising from the embryoid body have covered the surface of the dish, the cells were detached with TrypLE Express, passed through a 40 µm Cell Strainer to remove the remaining embryoid bodies and reseeded in 10 cm tissue in the same conditions. Cells were used after day 20 of differentiation.

Atomic force microscopy (AFM) measurements. Cells were seeded onto a 40 mm tissue culture (93040, Ibiotech) at a concentration of 100,000 cells per dish for 24 h. Force maps were measured using a bio-AFM Nanowizard 4XP (Bruker-JPK, Germany) placed on a Leica DMi 8 inverted microscope with a 10x objective (Leica, Germany). A 5.73 µm melamine sphere (microParticles, Berlin, Germany) was attached to a soft tipless cantilever SD-qp-CONT-TL (NanoWorld, Neuchâtel, Switzerland) using epoxy resin. A plastic Petri dish (TPP, Trasadingen, Switzerland) containing either distilled water for calibration or cell culture was placed on a motorized stage with a Petri dish heater pre-heated to 37 °C. Before each experiment, the laser reflection sum was maximized, and the laser detector was centered. Immediately after, probe sensitivity and stiffness were determined using the thermal noise method in Bruker-JPK software. AFM settings were kept constant for each sample, with a setpoint in the range of 0.2-0.8 nN relative to the baseline to always maintain indentation depth up to 2.8 µm, Z-length at 10 µm, speed of recording at 20 µm s-1, and sample rate at 5 kHz. Each force map consisted of 64x64 or 32x32 FDCs (Force-distance curves) covering an area of single or multiple cells, and Young´s modulus was calculated from the FDCs by fitting the DMT model [3] in AtomicJ software.[4]

Correlative Probe and Electron Microscopy measurements by LiteScope™. Correlative Probe and Electron Microscopy (CPEM) images were acquired using an AFM LiteScope (NenoVision, Czech Republic) integrated with a Versa 3D Dual Beam SEM (Thermo Fisher Scientific, Czech Republic). This setup allowed for the identification of particles on/in cells and navigation of the tip to areas of interest by electron imaging. SEM and AFM topography images were simultaneously acquired in NenoView software, showing differences in material and topological features. Topography was measured using a frequency-modulated tapping regime with an Akiyama probe

(Nanosensors), a self-sensing probe with a visible apex. At the same time, the electron beam was focused near the AFM tip to acquire secondary electron signals from the Ion Conversion and Electron (ICE) detector. All images were acquired with 512 points and a scanning speed between 15 µm/s and 2 µm/s, depending if large or small scanning fields were acquired, with a primary electron acceleration voltage of 10kV and current of 1.5pA. The offset between the SE image and AFM topography was then removed in NenoView software, using the same pixel size for correlation. Final post-processing was performed in Gwyddion software (Czech Republic).

Isolation of RNA and PCR analysis. Total RNA was isolated using High Pure RNA Isolation Kit (Roche, Switzerland), according to the manufacturer's protocol. Complementary DNA was synthesized using the RT^2 First Strand Kit (SABiosciences, Frederick, USA). The expression levels of genes involved in ECM and cell adhesion were analyzed using RT^2 Profiler PCR Arrays (Qiagen). Reverse transcription polymerase chain reaction (RT–PCR) was performed on the LightCycler 480 Real-Time PCR System (Roche, Basel, Switzerland), and the cycling parameters were 1 cycle at 95 °C for 10 min, followed by 45 cycles at 95 °C for 15 s and 60 °C for 1 min. The normalization of gene expression levels was performed using an internal panel of housekeeping genes provided by the manufacturer. The PCR-array data were analyzed online using the tools available on the manufacturer's website (http://www.sabiosciences. com/pcrarraydataanalysis. php). Genes with a high coefficient of variation among replicas or very low expression (35 < Ct > 40) were excluded from the analysis. The results are presented as heatmaps of quantification cycles (Ct) and graphs of mean ± standard deviation (s.d.) values of fold regulation, based on three samples per experimental condition.

Cell transfection. Cells were transfected using Lipofectamine 3000. The plasmids YAP1 (S6A)-V5 in pLX304 and pLX304 were obtained from Addgene as gifts from William Hahn (plasmid 42562) and David Root (plasmid 25890), respectively.[5] CAL51 cells were seeded onto a 6-well plate and transfected 24 h later with a preincubated mixture containing 250µL of Opti-MEM, 2.5 ng of DNA, 7.5 µl of Lipofectamine 3000, and 5 µL of P3000 reagent, added dropwise into each well. After 12 h, fresh medium was added, and cells were allowed to grow for another 8 h. The cells were then detached, seeded in a 24-well plate at a density of 200,000 cells/well, and cultured for an additional 12 h prior to incubation with nanoparticles.

Polystyrene nanoparticle internalization studies. The day before the experiments, 200,000 cells were seeded in 500 µL of medium onto a 24-well plate. For co-culture experiments, CAL51 YAP -/- cells were labeled with CellTracker Blue CMAC Dye according to the manufacturer's protocol. Then, 100,000 CAL51 WT and 100,000 pre-labeled CAL51 YAP -/- cells were seeded together onto a 24-well plate. For the experiments carried out using substrates with different stiffness, either a µ-Dish 35 mm, 1.5 ESS or a CytoSoft Imaging 24-well Plate with an Elastic Modulus of 2 kPa that were pre-coated with 0.1 mg/mL of collagen I rat tail or 30 µg/mL of bovine plasma fibronectin have been used. After 18 h, the cells were incubated with nanoparticles diluted at the desired concentration in the supplemented medium for 4 h (or according to the time indicated for each experiment), with 500 µL of nanoparticle dispersion added per well. The samples were then processed for downstream flow cytometry and confocal analysis according to the following protocols: for cytofluorimetric analysis, CAL51 WT or YAP -/- cells were cultured on 24-well plates and left for 12 h to adhere, then incubated with PS200 and PS900 for 4 h. The medium was removed, 200 µL of TrypLE Express Enzyme was added to each well for 5 min, and the cells were

detached and washed three times until no particle signal was detected in the supernatant. The samples were analyzed using the FACSAria II (Becton-Dickinson, USA), and plots were prepared with FlowJo software V10 (Tree Star, USA). For confocal laser scanning microscopy (CSLM) analysis, after the incubation with nanoparticles, the cells were detached, washed three times with PBS, and left to adhere to a µ-slide 8-well glass bottom dish. After 4 h, the cells were processed according to the *immunohistochemistry* protocol described below.

Doxorubicin-loaded liposome (Doxo-NP) internalization study. To evaluate the role of YAP in nanomedicine delivery studies, CA51 WT and YAP -/- cells were incubated with Doxo-NP for 4 h before examining cell-nanoparticle interactions. To evaluate the pharmacological inhibition of YAP, Doxo-NP internalization was examined in CAL51 WT cells pre-treated with 1 µM of CA3 inhibitor for 12 h. After replacing the media, the cells were incubated with Doxo-NP for another 4 h, and then analyzed by flow cytometry or CLSM, as previously described.

Immunohistochemistry and image analysis. For immunohistochemistry, 40,000 cells per well were seeded onto a µ-slide 8-well glass bottom dish for 24 h. After each relevant experiment, the medium was removed, and the cells were washed with PBS. Before staining, the cells were fixed with 200 µL 4% paraformaldehyde in PBS for 15 min at RT, permeabilized with 0.1% Triton X-100 for 5 min, and then blocked with BSA 2.5% in PBS for 30 min. The primary antibodies were added in 200 µL PBS-BSA 2.5% solution and incubated for 2 h at RT or O.N. at 4 °C. Afterward, the secondary Alexa fluorochrome-conjugated antibodies were added, and the cells were incubated in PBS. F-actin was stained with Alexa Fluor 488 or 647-conjugated phalloidin, the membrane was stained with wheat germ agglutinin (WGA) 488- or 647-conjugated, and the nuclei were counterstained with 4',6'-diamidino-2-phenylindole (DAPI) or Hoechst. The samples were embedded in Mowiol reagent (Merck) and visualized with a Zeiss LSM 780 or Leica TCS SP8 X confocal microscope. Z-stacks were acquired with an optimal interval suggested by the software, and a maximum intensity algorithm was applied. Images were analyzed using ImageJ (http://rsb.info.nih.gov/ij/). The primary antibodies used were mouse anti-YAP (4912, Cell Signaling), mouse anti-Vinculin (V9131, Merck), anti-Col1A1 (91144S, Cell Signaling), anti-Col3A1 (PA5-27828, Thermo Fisher), anti-CTGF (Ab5097, Abcam), anti-Fibronectin (F3648, Merck), anti-Laminin (SAB4200719, Merck), and anti-Periostin (49480, Santa Cruz), as referred in the Materials sections and in Supplementary Table 1.

Western blotting. Cells were lysed with RIPA buffer (Merck Millipore) containing 1% protease and phosphatase inhibitor cocktails on ice, and then centrifuged at 14.000 g for 15 min at 4 °C. The supernatants were stored at 80 °C. Protein concentrations were determined using the Bicinchoninic Acid (BCA) protein assay, and 20 µg of protein per sample were loaded onto 10% polyacrylamide gels and run at 100V. Proteins were transferred to a polyvinylidene difluoride membrane using the Trans-Blot Turbo transfer system (Bio-Rad). The membranes were blocked with 5% BSA in TBST and incubated with primary antibodies diluted in 5% BSA in TBST O.N. at 4 °C. They were then probed with proper HRP-linked secondary antibodies for 1 h at RT. Chemiluminescence was detected using the ChemiDoc imaging system (Bio-Rad), and band intensities were quantified using Bio-Rad Image Lab software. The primary antibodies used were rabbit anti-YAP, mouse anti-β-tubulin, mouse anti-vinculin, rabbit anti-pYAP S127, rabbit anti-pH2A.X, and rabbit anti-cPARP.

Scanning electron microscopy. WT and YAP -/- CAL51 cells were cultured on coverslips for 2 days, fixed with a solution of 3% Glutaraldehyde in 100mM Cacodylate buffer, and dehydrated in a series of increasing ethanol concentrations of 30%, 50%, 70%, 80%, 96%, and 100%. The samples were mounted on aluminum stubs, sputter-coated with Palladium (JEOL JFC-1300, Tokyo, Japan), and visualized using a Benchtop Scanning Electron Microscope JEOL JCM-6000 (Tokyo, Japan).

Transmission electron microscopy. WT or YAP -/- CAL51 cells were grown on 24-well plates and left for 12 h to allow for adhesion, then incubated with nanoparticles for 4 h in a growth medium. Afterward, the cells were washed, detached, and seeded for an additional 4 h. Fixation was done by 1.5% PFA + 1.5% glutaraldehyde diluted in DMEM high glucose culture media for 1 h, then washed with 0.1 M cacodylate buffer (pH 7.4) for 3 times 5 min each, and incubated overnight at 4 °C in cacodylate 0.1 M with 1.5% glutaraldehyde. The next day, samples were rewashed 3 times with cacodylate buffer, and incubated at 4 °C in cacodylate buffer until sending the samples, protected from light. The obtained cell pellet was post-fixed for 1.5 h with 1% osmium tetroxide in 0.1 M cacodylate buffer and washed for 3 times with 0.1 M cacodylate buffer, 10 min each, then stained with 1% uranyl acetate in milli-Q water overnight at 4 °C, followed by washing in milli-Q water. The samples were then dehydrated in an ascending EtOH series using solutions of 70%, 90%, 96% and 3 times 100% for 10 min each, incubated in propylene oxide (PO) 3 times for 20 min before incubation in a mixture of PO and Epon resin overnight, and incubated in pure Epon for 2 h and embedded by polymerizing Epon at 68 °C for 48 h. Ultra-thin sections of 70 nm were cut using a Leica Ultracut EM UC 6 Cryo-ultramicrotome. TEM images were collected with a JEOL JEM 1011 electron microscope and recorded with a 2 Mp charge coupled device camera (Gatan Orius).

Mechanism of nanoparticle endocytosis study. To investigate the mechanism of nanoparticle endocytosis, the cells were cultures in a medium containing endocytosis inhibitors chlorpromazine hydrochloride (10 μg/ml), nystatin (100 U/ml), or cytochalasin D (1 μg/ml) for 2 h, then incubated with polystyrene nanoparticles for 4 h. After incubation, the cells were processed for flow cytometry. Endo-/lysosomal trafficking was also examined by adding LysoTracker Deep Red to the culture media until a final concentration of 100 nM, with the cells incubated for endo-/lysosome staining for 1 h according to the manufacturer's protocol. The cells were then processed for immunohistochemistry analysis according to the previously described protocol.

RNA Sequencing (RNA-seq) and Data Analysis. The library was prepared using the NEBNext® Ultra™ II Directional RNA Library Prep Kit for Illumina® with NEBNext® Poly(A) mRNA Magnetic Isolation Module and NEBNext® Multiplex Oligos for Illumina® (Dual Index Primers Set 1). The kits were employed according to the manufacturer's protocol, and 200-300 ng of total RNA was used as input to prepare the library. Sequencing was performed on an Illumina NextSeq 500 using the NextSeq 500/550 High Output v2 kit (75 cycles). Single-end 75bp sequencing was carried out in multiple sequencing runs until all samples had at least 30 million passing filter reads. Fastq files were generated using bcl2fastq software without any trimming. The quality of the raw sequencing data was assessed using FastQC (https://www.bioinformatics.babraham.ac.uk/projects/fastqc/) and aligned to the hg38 reference genome using TopHat2 aligner. Raw gene counts were calculated from reads mapping to exons, summarized by genes using the Ensembl 90 reference gene annotation (Homo sapiens GRCh38.p10, GTF) by HTSeq. Differential gene expression was determined using the DESeq2 Bioconductor package. Genes were

considered differentially expressed if a Benjamini-Hochberg adjusted P-value was ≤ 0.05 and log2 fold-change (log2FC) was ≥ 1.5. Biological term classification and gene cluster enrichment analysis were performed using the clusterProfiler package. All computations were performed using BioJupies.[6] Enrichment analysis and ranking were performed using Enrichr, and the most significant annotations were downloaded from the available repository (https://maayanlab.cloud/Enrichr/) [7-9]. The Gene Ontology (GO) categories were downloaded from the AmiGO 2 repository (https://amigo.geneontology.org).

Minimum Information Reporting in Bio−Nano Experimental Literature (MIRIBEL). The studies conducted here, including material characterization, biological characterization, and experimental details, conform to the MIRIBEL reporting standard for bio−nano research.[10] A companion checklist for the MIRIBEL components is provided in the *Supporting Information* (Tables 2-4).

Image processing. Images have been processed using FIJI, Imaris (version 10.0.1) and LAS X softwares. Using Imaris, stack images were first converted to imaris files with ImarisFileConverter, and 3D reconstruction was performed with the 'volume rendering' function. Optical slices were obtained with the 'orthoslicer' tool.

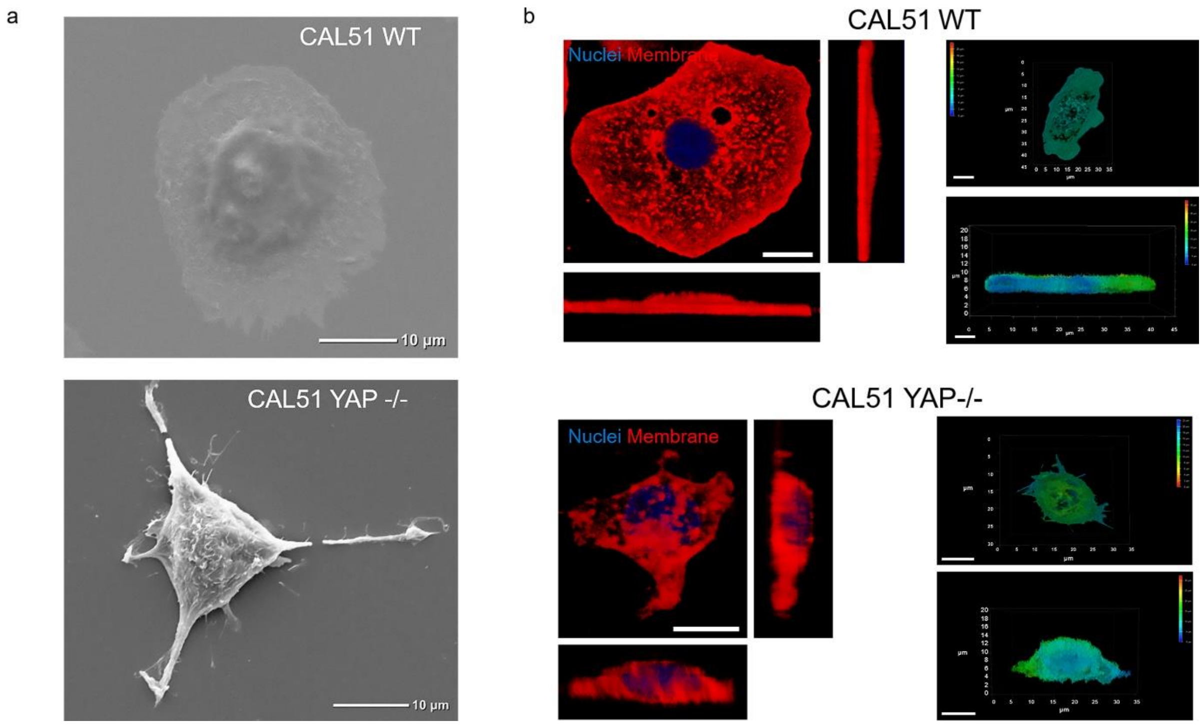

Figure S1. a) Representative SEM images of WT and YAP -/- CAL51 cells. Scale bar: 10 µm. b) 3D reconstruction of WT (top) and YAP -/- (bottom) CAL51 cells. The top and lateral views are presented. The cells on the left are stained with DAPI (blue) and WGA-647 (red). The cells on the right are presented in depth color code. Scale bar: 20 µm.

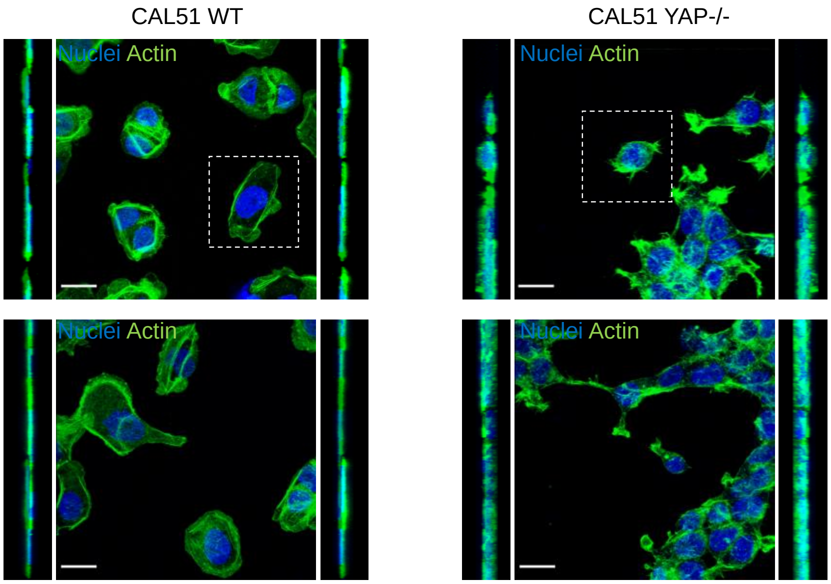

Figure S2. 3D reconstruction of WT (left) and YAP-/- (right) cell populations. Top and lateral views are presented. The cells are stained for nuclei (DAPI, blue) and Pha-488 (green). The white boxes highlight the cells chosen for the panel of Figure 1F, in the main text. Scale bar: 20 μm.

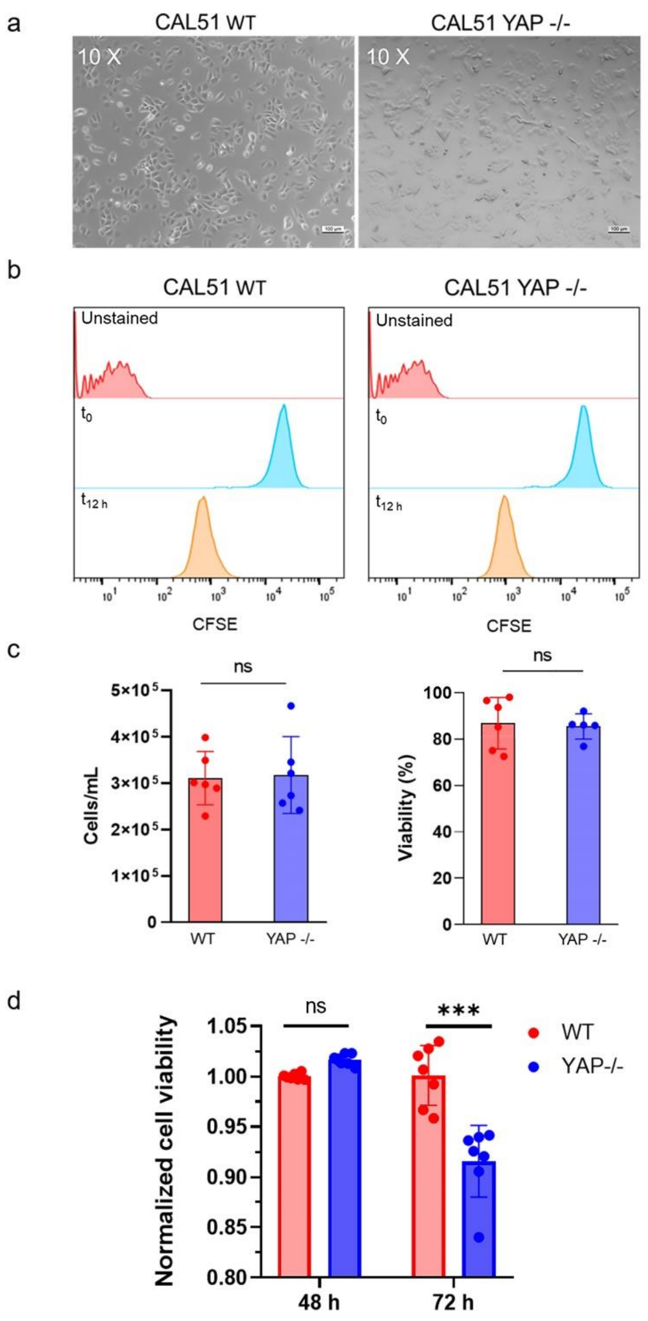

Figure S3. a) Representative brightfield images of WT and YAP -/- CAL51 cells seeded onto 24-well plates 12 hours after seeding. Scale bar: 100 μm. b) Histogram of the CFSE assay for WT (left) and YAP -/- (right) cells 12 hours after seeding. Unstained cells are presented in red, cells right after seeding are presented in blue, and cells after 12 hours of seeding are presented in orange c) Cell number and viability measured *via* the trypan blue assay 12 hours after WT (red) and YAP -/- (blue) cell seeding. n = 6. Statistical analysis was performed by unpaired t-test with Welch's correction. ns indicates non-significant. d) Presto blue viability assay performed on CAL51 WT (red) and CAL51 YAP-/- (blue) cells at 48 and 72 hours of culturing. Statistical analysis was performed using the two-way ANOVA followed by Sidak's multiple comparisons test. n = 7; *** indicates $p < 0.001$.

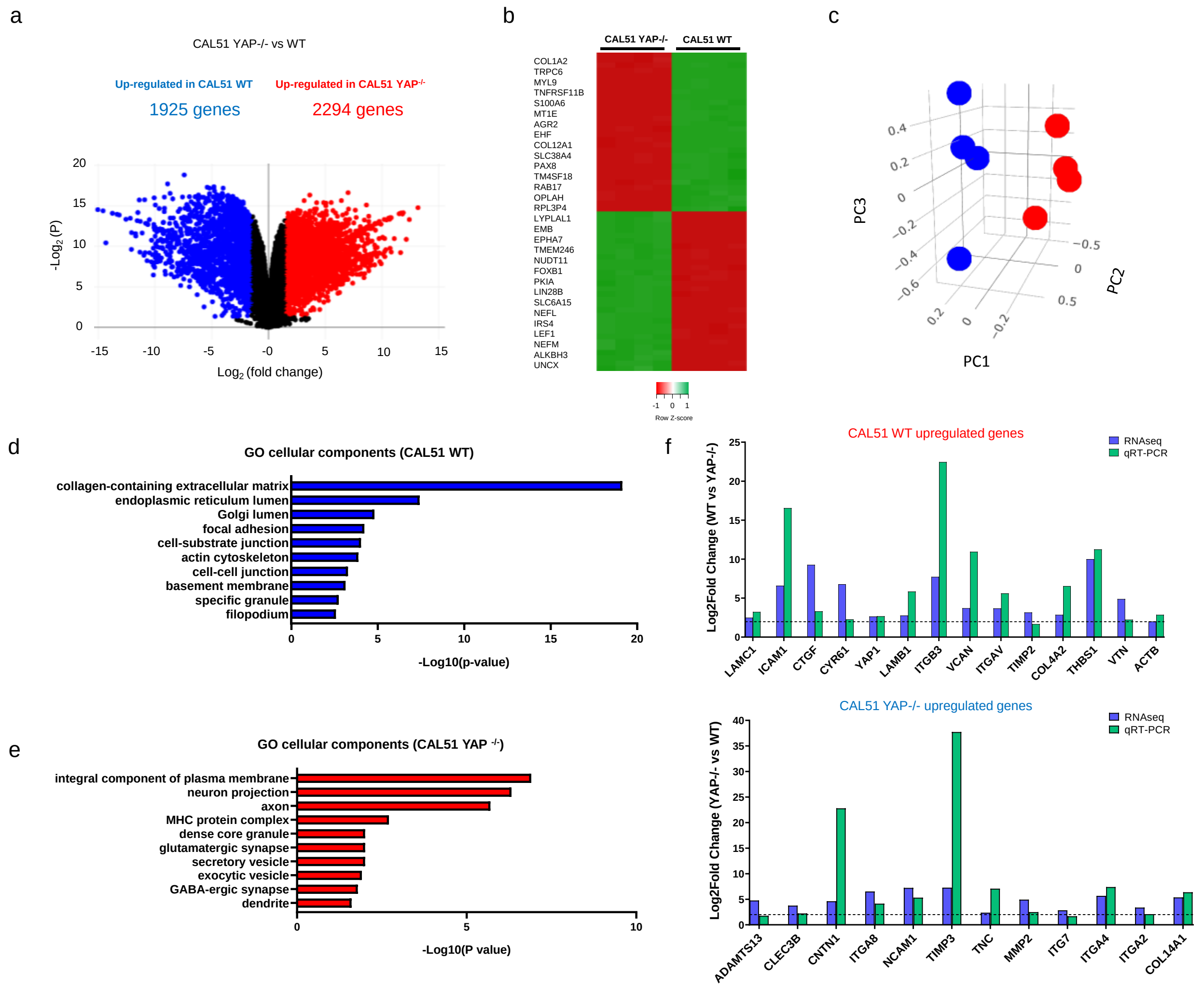

Figure S4. a) Volcano plot showing differential gene expression in WT vs. YAP -/- CAL51 cells. Red points indicate significantly upregulated genes, and blue points indicate downregulated genes. N = 4 (P adj < 0.05, log2Fc > |1|). b) Heatmap of the relative gene expression of the 30 most differentially expressed genes in CAL51 WT cells compared to CAL51 YAP -/- cells (P adj < 0.05, log2Fc > |1|). c) 3D principal component analysis (PCA) of RNA-seq in CAL51 WT and CAL51 YAP -/- cells. Red dots represent a sample of WT cells, while blue dots represent a sample of YAP -/- cells. n = 4 (P adj < 0.05, log2Fc > |2|). The analysis was performed *via* Biojupies[6] d) Bar plot representation of common enriched cellular components obtained from the ENRICHR database,[7-9] displaying the most significantly upregulated genes in CAL51 WT compared to CAL51 YAP -/- cells (P adj < 0.05, log2Fc > |2|). e) Bar plot representation of common enriched cellular components obtained from the ENRICHR database, displaying the most significantly upregulated genes in CAL51 YAP -/- compared to CAL51 WT cells (P adj < 0.05, log2Fc > |2|). f) Validation of RNA-Seq data for differential gene expression by qRT-PCR. qRT-PCR analysis was performed on selected RNAs from CA51 WT and YAP-/- cells. The plots represent the log2 fold-change in expression for selected targets obtained from RNA-Seq (blue) and from qRT-PCR (green).

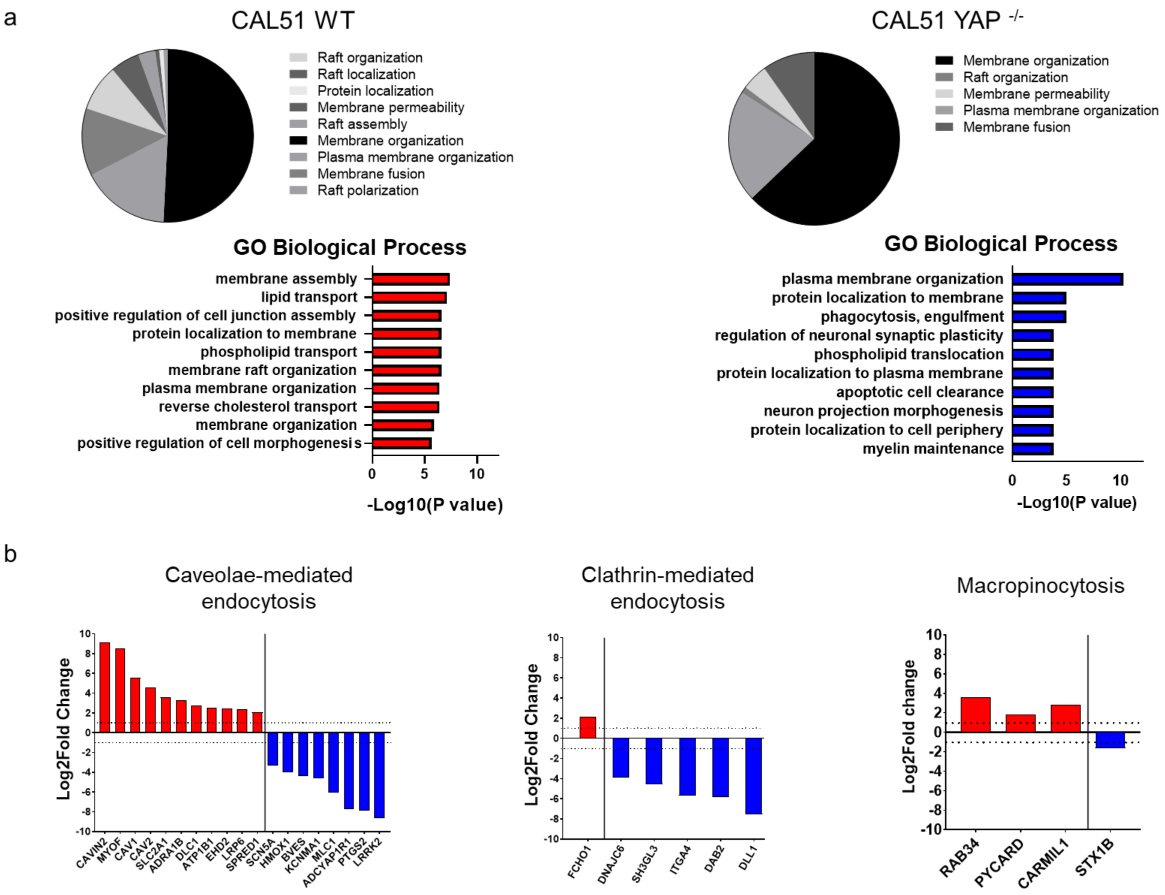

Figure S5. a) Pie chart of the annotations (top) and the bar plot representation (bottom) of common enriched biological processes and pathways obtained from the ENRICHR database [7-9], presenting the most significantly upregulated genes in WT (left) and YAP -/- (right) CAL51 cells involved in the membrane organization network (P adj < 0.05, log2Fc > |2|). b) Bar plot representations of the normalized expression of genes involved in caveolae-mediated, clathrin-mediated endocytosis and macropinocytosis found differentially regulated in YAP -/- cells as compared to WT CAL51 cells. (P adj < 0.05, log2Fc > |1|).

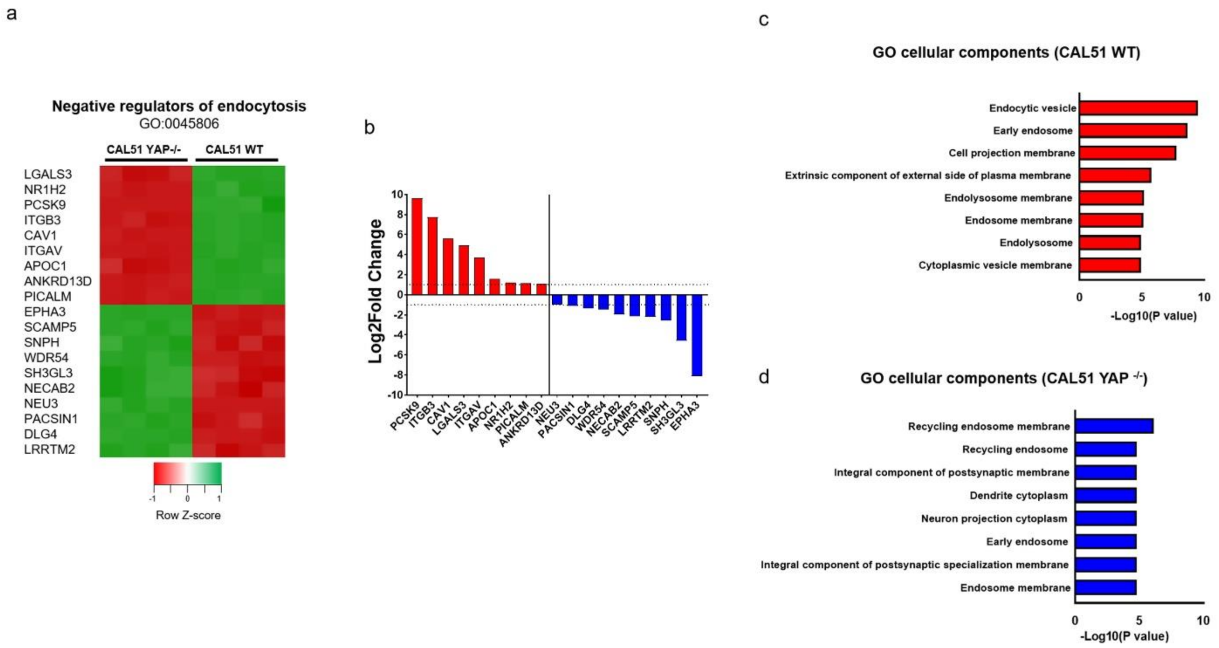

Figure S6. Heatmap (a) and relative normalized expression (b) of genes involved in the negative regulation of endocytosis (GO:0045806) (P adj < 0.05, log2Fc > |1|). c) Bar plot representation of common enriched cellular components among the negative regulators of endocytosis obtained from the ENRICHR database [7-9], presenting the most significantly upregulated genes in WT compared to YAP -/- CAL51 cells, and d) viceversa (P adj < 0.05, log2Fc > |1|).

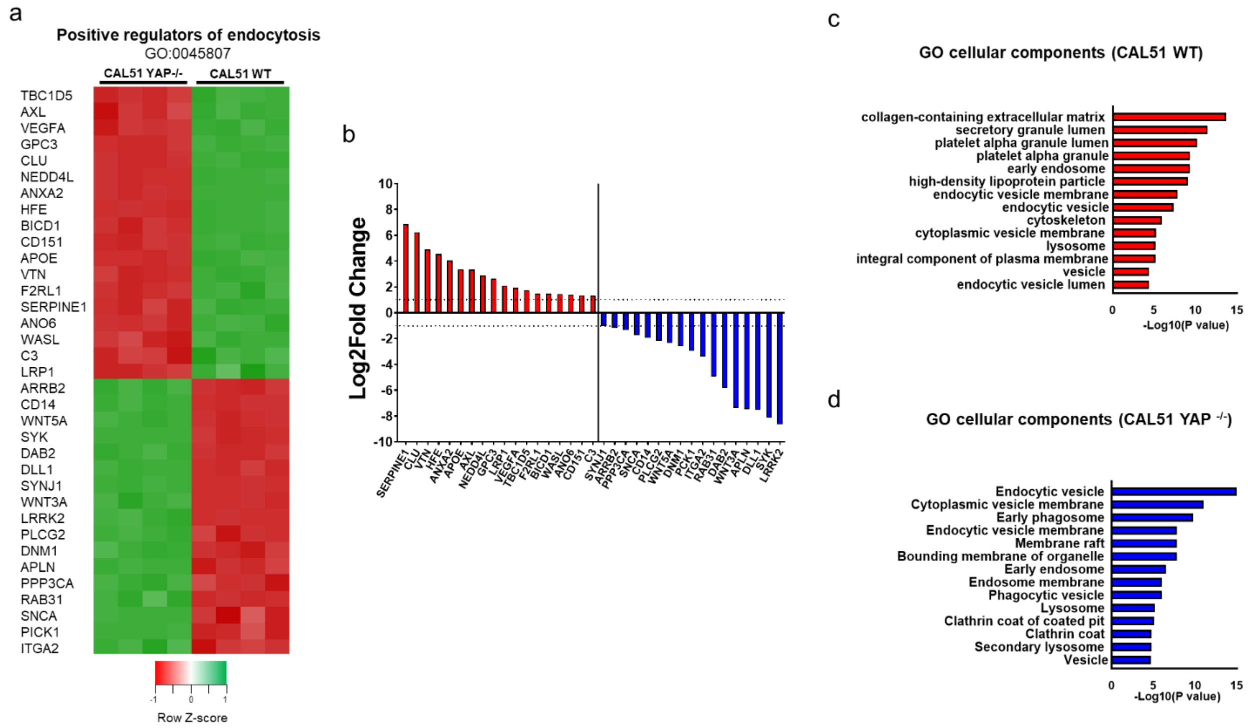

Figure S7. Heatmap (a) and relative normalized expression (b) of genes involved in the positive regulation of endocytosis (GO:0045807) (P adj < 0.05, log2Fc > |1|). c) Bar plot representation of common enriched cellular components for the positive regulators of endocytosis obtained from the ENRICHR database [7-9], considering the most significant upregulated genes in WT compared to YAP -/- CAL51 and d) viceversa (P adj < 0.05, log2Fc > |1|).

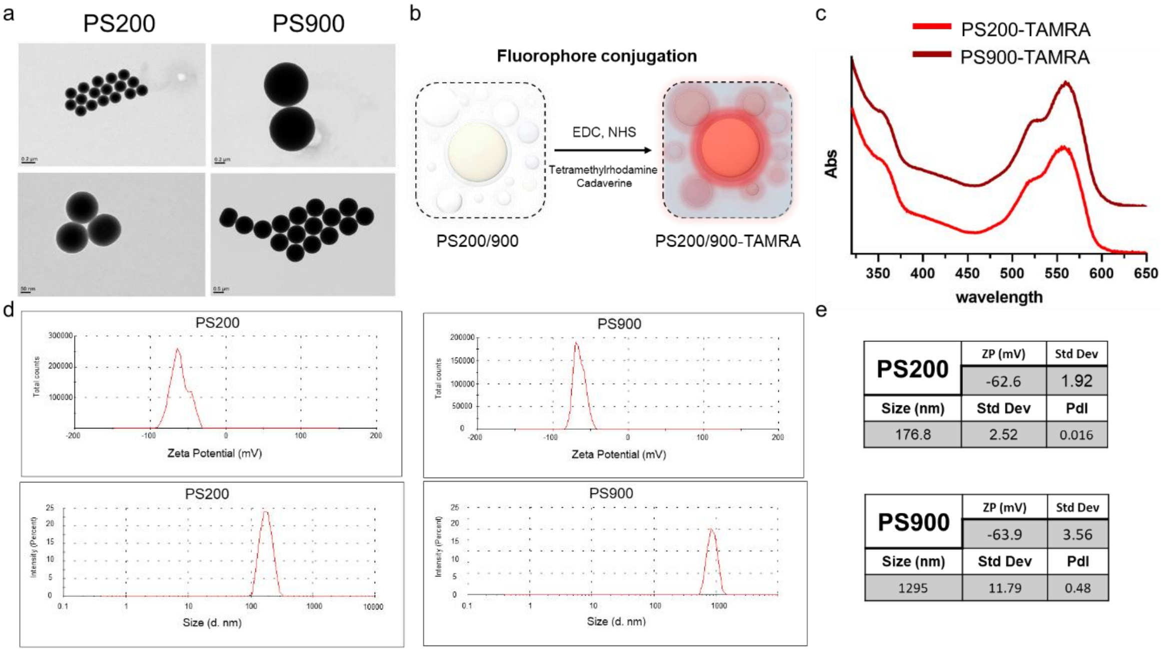

Figure S8. a) Representative TEM micrographs of 200 and 900 nm polystyrene nanoparticles. Scale bars: 50, 200, and 500 nm. b) Functionalization reaction of polystyrene nanoparticles with fluorescent molecules Tetramethylrhodamine-5-carboxamide cadaverine (TAMRA cadaverine) *via* EDC chemistry. c) Normalized UV-vis absorption spectra of TAMRA-functionalized PS200 (red line) and PS900 (dark red line) nanoparticles. d) DLS graph showing the zeta potential (top) and size weighted by intensity (bottom) of PS200 and PS900 in 10 mM NaCl. e) Tables reporting the zeta potential (ZP), hydrodynamic diameter (d_H), and polydispersity index (PdI) of PS200 (top) and PS900 (bottom) nanoparticles.

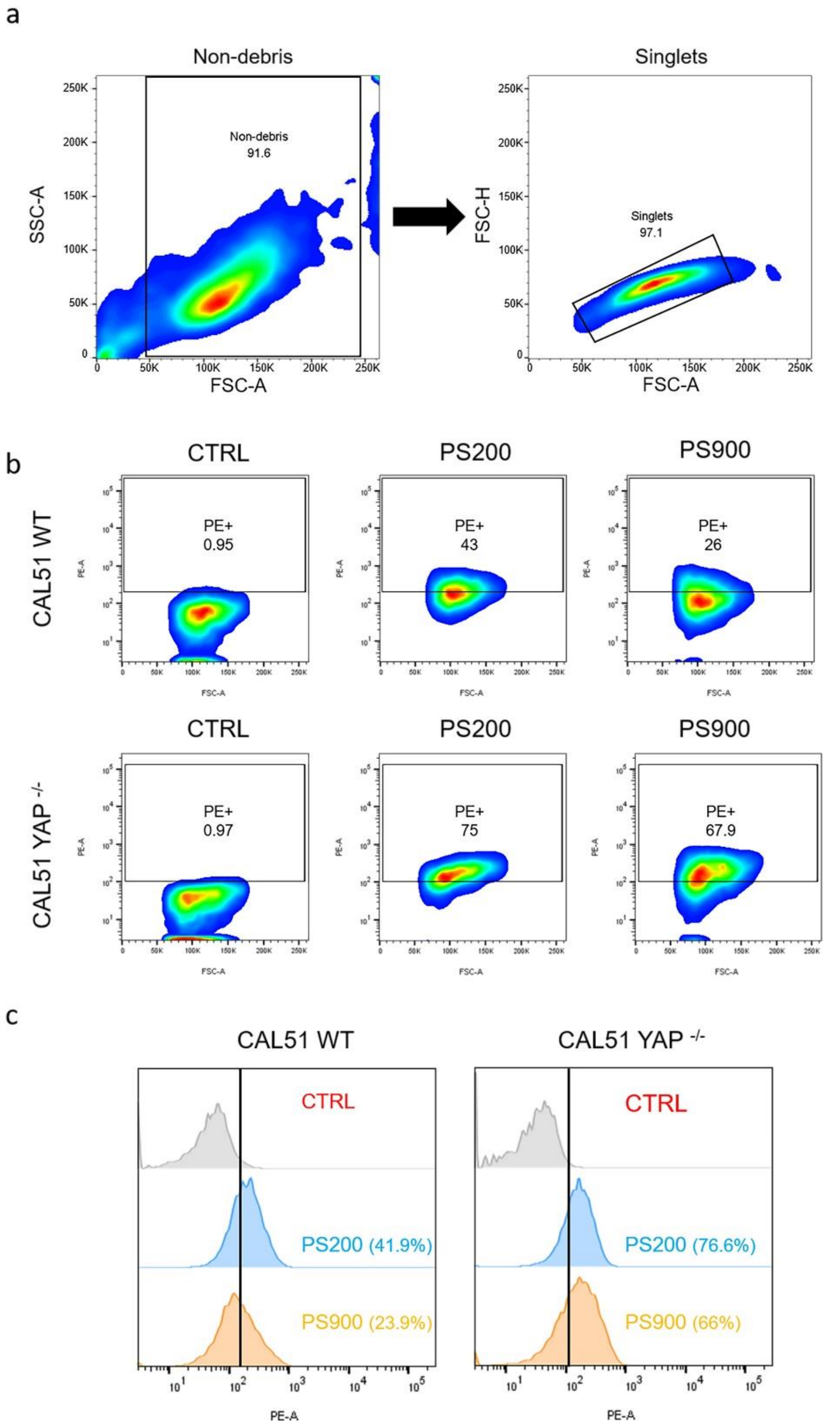

Figure S9. a) Flow cytometry gating strategy for CAL51 cells. b) Representative flow cytometry plots of WT (top) and YAP -/- CAL51 cells incubated for 4 hours with PS200 and PS900. c) Representative histograms of WT (left) and YAP -/- (right) CAL51 cells incubated for 4 hours with PS200 (blue) and PS900 (orange), displaying the gating applied for PE (nanoparticle)-positive cells.

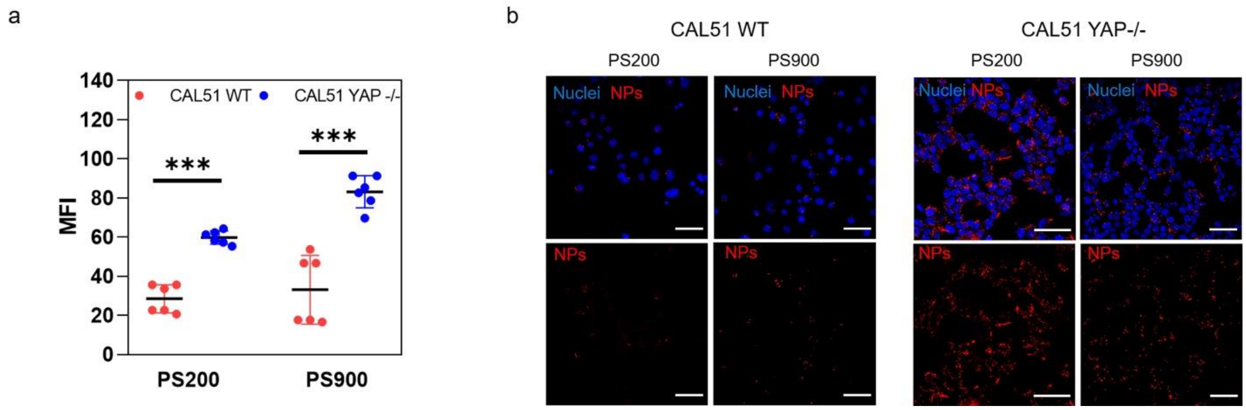

Figure S10. a) Median fluorescence intensity (MFI) of a 4-hour cellular uptake of PS200 and PS900 in CAL51 WT (red) and CAL51 YAP -/- (blue) cells. Statistical analysis was performed using the two-way ANOVA followed by Sidak's multiple comparisons test. n = 6; *** indicates $p < 0.001$. b) Confocal images of WT (left) and YAP -/- (right) CAL51 cells after a 4-hour incubation with PS200 and PS900. Cells were stained with DAPI (blue). Nanoparticles are displayed in red. Scale bar: 50 µm.

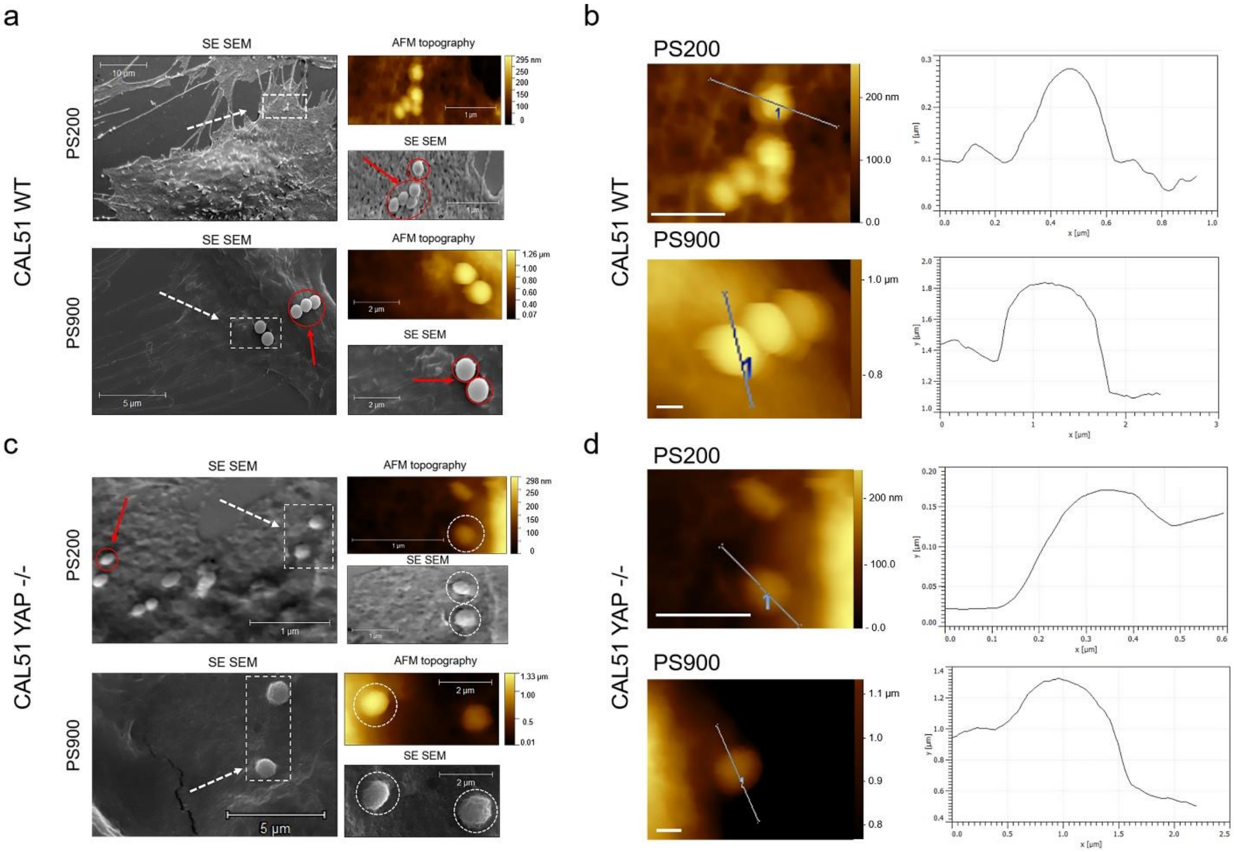

Figure S11. Topographical characterization of the cell-nanoparticle membrane interaction at the membrane. a) and c) Correlative Probe and Electron Microscopy (CPEM) imaging of WT (a) and YAP -/- (c) CAL51 cells treated for 4 hours with PS200 and PS900. AFM and SEM images are presented. White dashed line boxes present magnified AFM and SEM images on the right of each micrograph. Red arrows and circles show nanoparticles bound to the cells but not embedded in the plasma membrane; white dashed arrows and circles show nanoparticles embedded in the cell membrane undergoing endocytosis. b) and d) show details of AFM images from WT (b) and YAP -/- (d) CAL51 cells incubated for 4 hours with PS200 (top) and PS900 (bottom). For each image, a plot on the right displays the height profile for the area marked by the white dashed line. All the lines where extracted using a 3 px thick line for noise reduction. The one-dimensional texture is split into waviness, reflecting the low-frequency shape components. Scale bar: 0.5 µm.

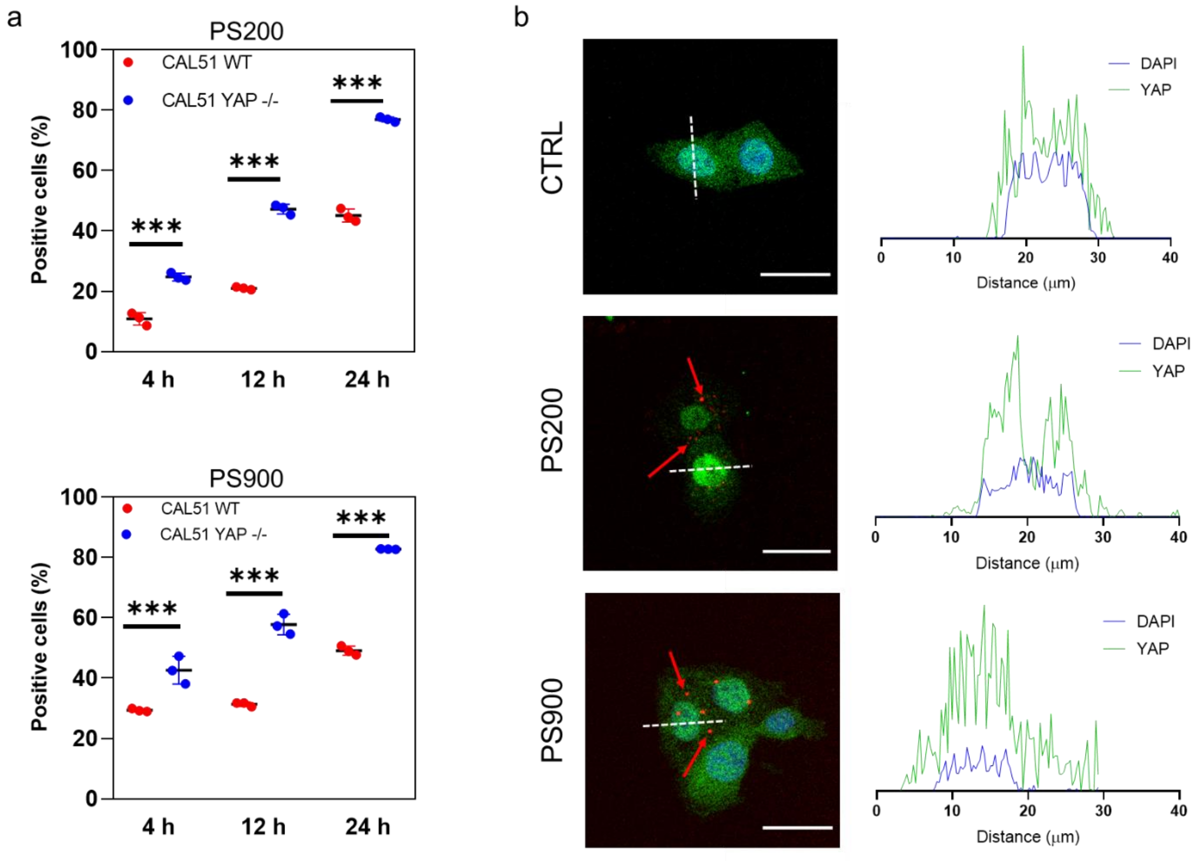

Figure S12. a) 4-, 12-, and 24-hours cellular uptake of PS200 and PS900 in WT (red) and YAP -/- (blue) CAL51 cells. Statistical analysis was performed using the two-way ANOVA followed by Tukey's multiple comparisons test. n = 3; *** indicates $p < 0.001$. b) Confocal images and the intensity profile for the area marked by the white dashed line in WT CAL51 cells after 4-hour incubation with PS200 and PS900. Cells are stained with YAP (AF488) and DAPI (blue). Nanoparticles are displayed in red and pointed at by red arrows. Scale bar: 50 μm.

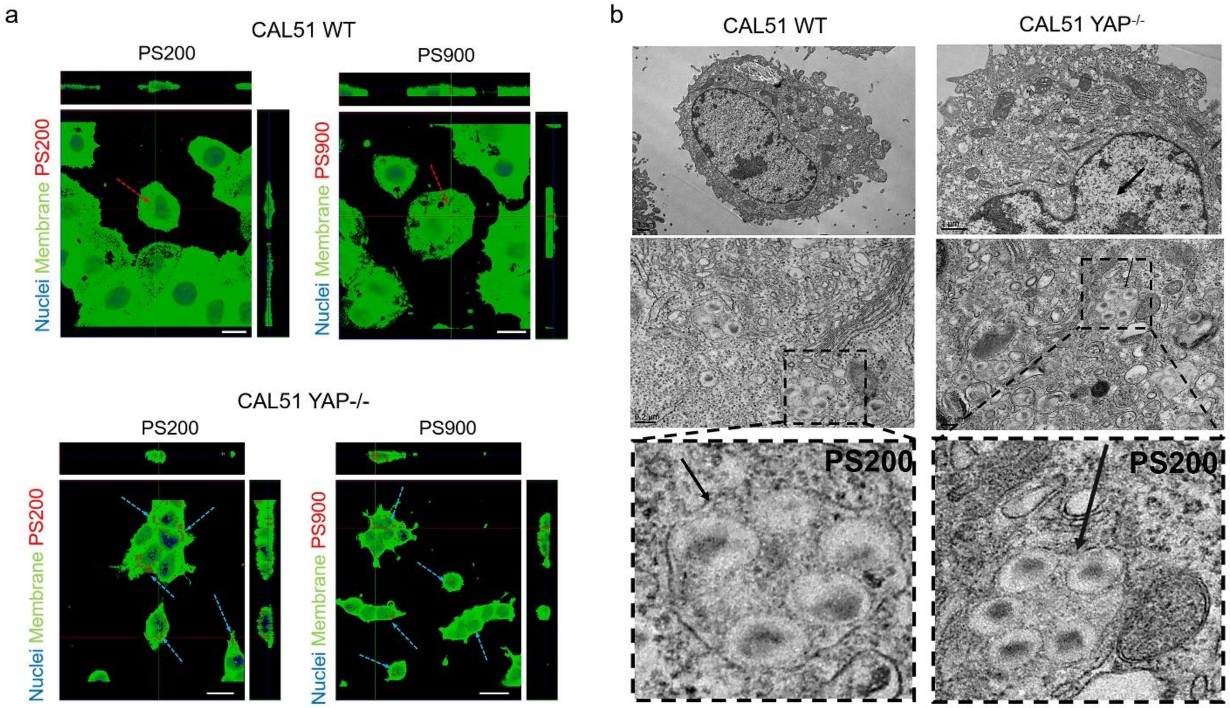

Figure S13. a) Orthogonal view of z-projections of WT (top) and YAP -/- (bottom) CAL51 after 4 hours of incubation with PS200 and PS900. Cells are stained with WGA-488 (green) and DAPI (blue). Red dashed arrows indicate nanoparticles found in WT, and blue dashed arrows indicate nanoparticles found in YAP -/- CAL51. Scale bar: 20 μm. b) Thin-section TEM images of WT (top) and YAP -/- (bottom) CAL51 cells incubated with PS200 for 4 hours. Magnified images are displayed inside black dashed line boxes for each cell type. Nanoparticles are pointed at by black arrows. Scale bars: 1 and 0.2 μm.

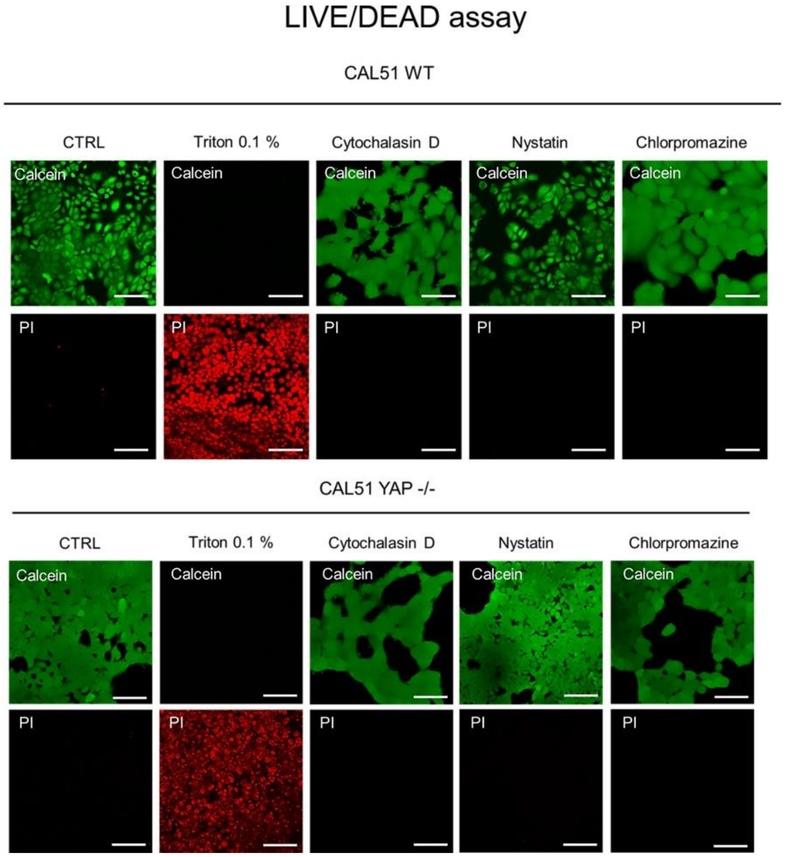

Figure S14. The live/dead assay performed on WT (top) and YAP -/- (bottom) CAL51 cells treated with endocytosis inhibitors Cytochalasin D, Nystatin, and Chlorpromazine for 6 hours. As a control for cell death, cells were heated for 15 minutes with 0.1% Triton X-100 solution. Cells were excited with 555 nm laser (propidium iodide, PI, red) and 488 nm laser (calcein, green). Scale bar: 100 µm.

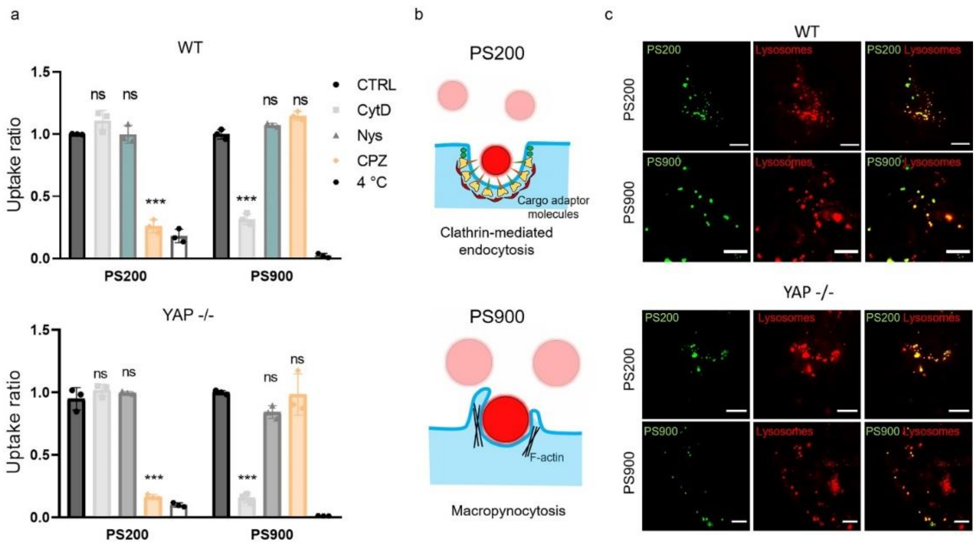

Figure S15. The landscape of endocytic pathways in WT and YAP -/- CAL51 cells. a) Uptake ratios for PS200 and PS900 in WT (top) and YAP -/- (bottom) CAL51 cells upon treatment with endocytosis pathway inhibitors cytochalasin D (CytD), Nystatin (Nys), and chlorpromazine (CPZ), or incubated with nanoparticles at 4° C. Statistical analysis was performed using the two-way ANOVA followed by Sidak's multiple comparisons test. $n = 3$; *** indicates $p < 0.001$. b) PS200 are mainly internalized by the cells *via* clathrin-mediated endocytosis (top), while PS900 undergo macropinocytosis (bottom). c) Confocal images of the intracellular localization of PS200 (top) and PS900 (bottom) in CAL51 WT (left) and CAL51 YAP -/- (right) cells 8 hours after 4-hour incubation with the nanoparticles. Cells are stained with Lysotracker. Nanoparticles are displayed in green. Scale bar: 10 μm

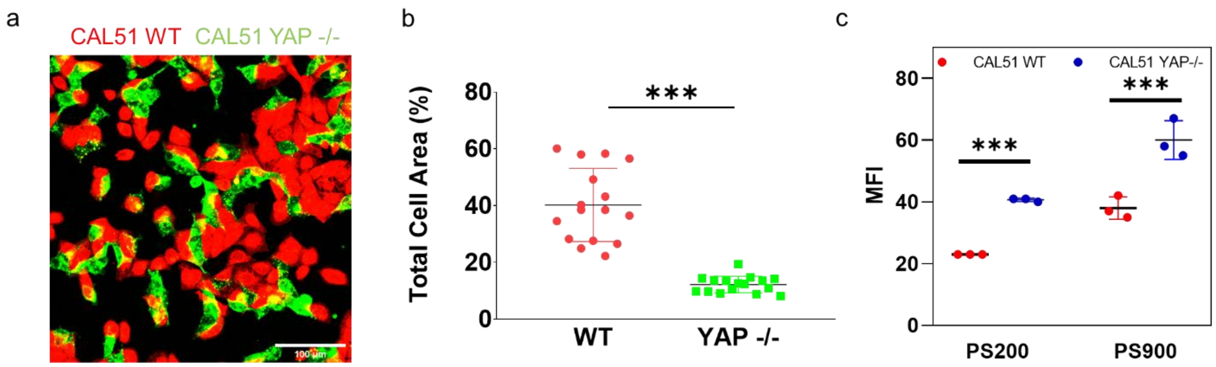

Figure S16. a) Confocal image of the WT and YAP -/- CAL51 co-culture. WT cells are stained with PKH-red, and YAP -/- cells are stained with PKH-green. Scale bar: 100 µm. b) Area of WT and YAP -/- CAL51 cells in a co-culture calculated based on the total membrane area of the cells. Statistical analysis was performed by unpaired t-test with Welch's correction. $n > 10$; *** indicates $p < 0.001$. c) Median fluorescence intensity (MFI) of a 4-hour cellular uptake of PS200 and PS900 for WT (red) and YAP -/- (blue) CAL51 cells in co-culture. Statistical analysis was performed using the two-way ANOVA followed by Sidak's multiple comparisons test. $n = 3$; *** indicates $p < 0.001$.

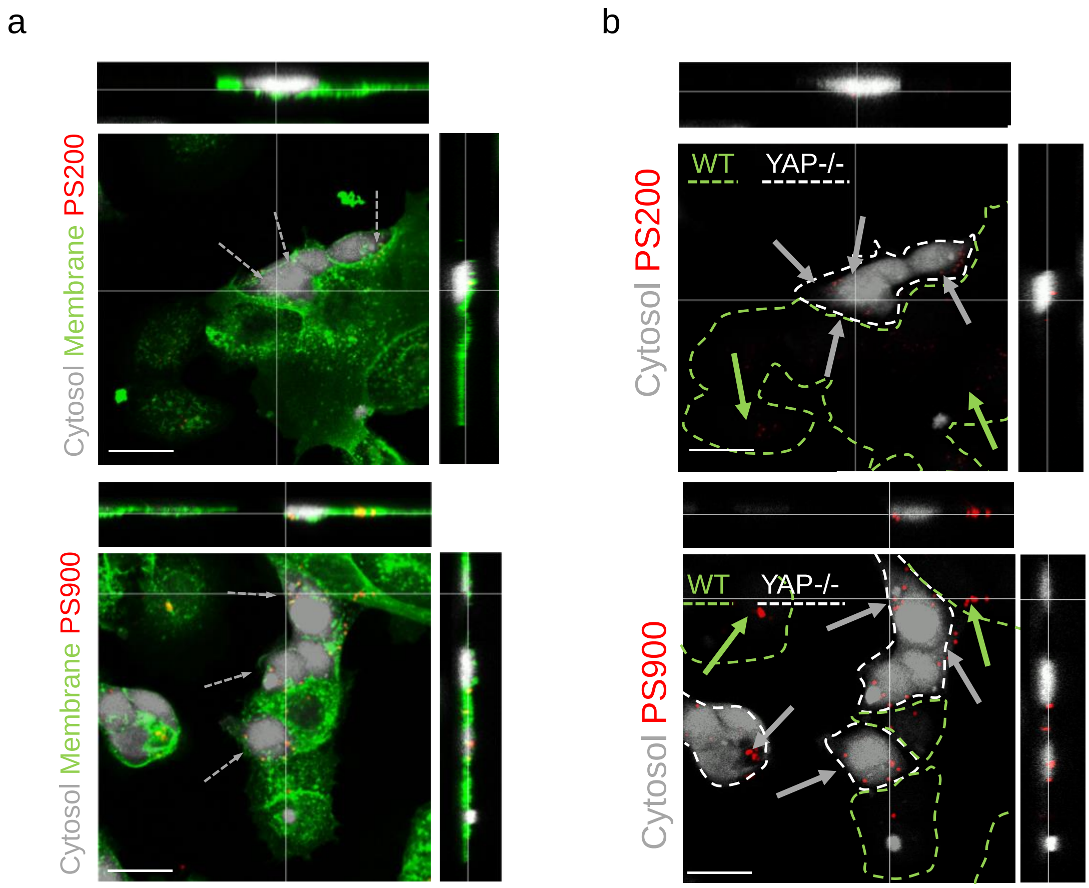

Figure S17 a) Orthogonal view of z-projections of the co-culture after 4 hours of incubation with PS200 (top) and PS900 (bottom). Grey dashed arrows indicate the particles found in YAP -/- CAL51 cells. YAP -/- cells are stained with 7-amino-4-chloromethylcoumarin (grey) and whole cell population with WGA-488 (green). Scale bar: 20 µm. b) Orthogonal view of the Z-projections of the co-culture after 4 hours of incubation with PS200 (top) and PS900 (bottom). Grey arrows point at the nanoparticles found in YAP -/- cells (encircled with white dashed lines), while green arrows point to the particles found in WT CAL51 cells (encircled with green dashed lines). YAP -/- CAL51cells were stained with 7-amino-4-chloromethylcoumarin (grey). Scale bar: 20 µm.

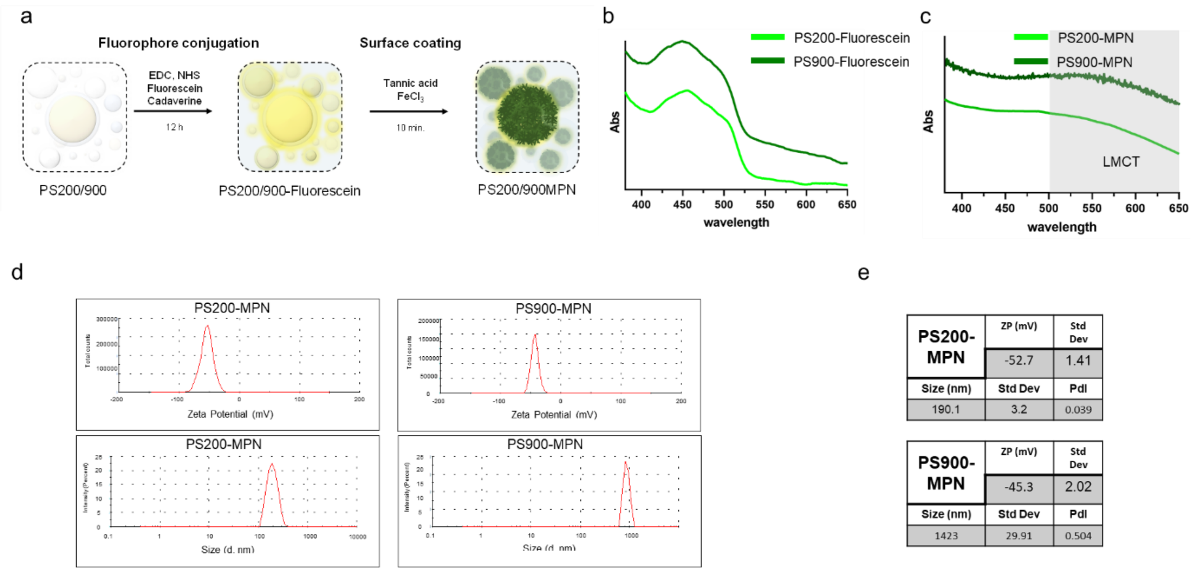

Figure S18. a) Functionalization reaction of polystyrene nanoparticles with fluorescent molecules Tetramethylrhodamine-5-carboxamide cadaverine (fluorescein cadaverine) *via* EDC chemistry and the subsequent metal phenolic network (MPN) formation using tannic acid (TA) and iron chloride ($FeCl_3$). b) Normalized UV-vis absorption spectra of fluorescein-functionalized PS200 (green line) and PS900 (dark green line) nanoparticles. c) Normalized UV-vis absorption spectra of MPN-coated fluorescein-functionalized PS200 (green line) and PS900 (dark green line) nanoparticles. The shadowed area indicates the absorbance region of the ligand-to-metal charge transfer (LMCT) of the particles coated with MPN. d) DLS graph showing the zeta potential (top) and size weighted by intensity (bottom) of PS200 and PS900 in 10 mM NaCl. e) Tables reporting the zeta potential (ZP), hydrodynamic diameter (d_H), and polydispersity index (PdI) of PS200-MPN (top) and PS900-MPN (bottom) nanoparticles.

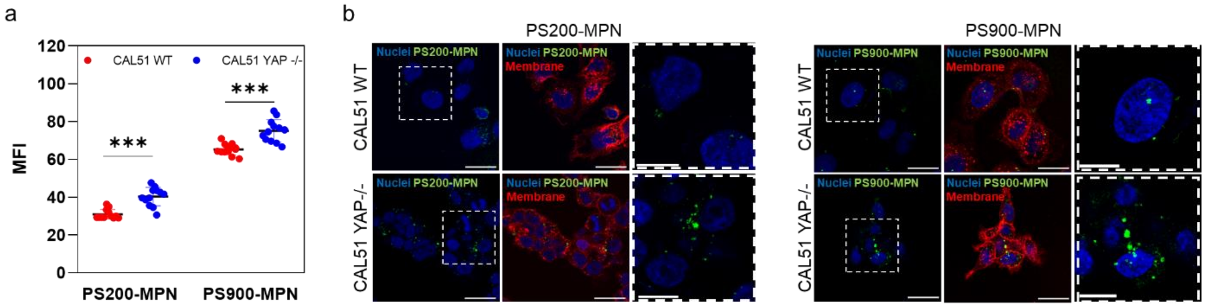

Figure S19. b) Median fluorescence intensity (MFI) of a 4-hour cellular uptake of PS200-MPN and PS900-MPN for WT (red) and YAP -/- (blue) CAL51cells. Statistical analysis was performed using the two-way ANOVA followed by Tukey's multiple comparisons test. n = 12; *** indicates $p < 0.001$. b) Confocal images of WT (left) and YAP -/- (right) CAL51 cells after 4-hour incubation with PS200-MPN and PS900-MPN. Cells are stained with WGA-647 (red) and DAPI (blue). Nanoparticles are displayed in red. Magnified images are presented inside the white dashed line boxes on the right relative to the highlighted sections on the left. Scale bars: 20 and 10 µm.

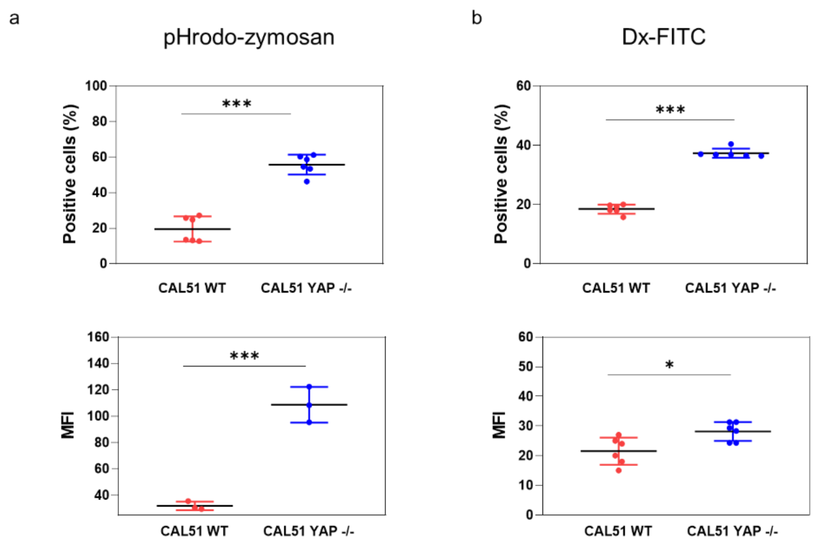

Figure S20. a) 4-hour cellular uptake of pHrodo-zymosan expressed as a percentage of positive cells (top) and median fluorescence intensity (MFI) for WT (red) and YAP +/+ (blue) CAL51 cells. Statistical analysis was performed using the unpaired t-test with Welch's correction. n = 6 and n = 3; *** indicates $p < 0.001$. b) 4-hour cellular uptake of Dx-FITC expressed as a percentage of positive cells (top) and MFI for WT (red) and YAP +/+ (blue) CAL51 cells. Statistical analysis was performed using the unpaired t-test with Welch's correction. n = 6; * indicates $p < 0.05$; *** indicates $p < 0.001$.

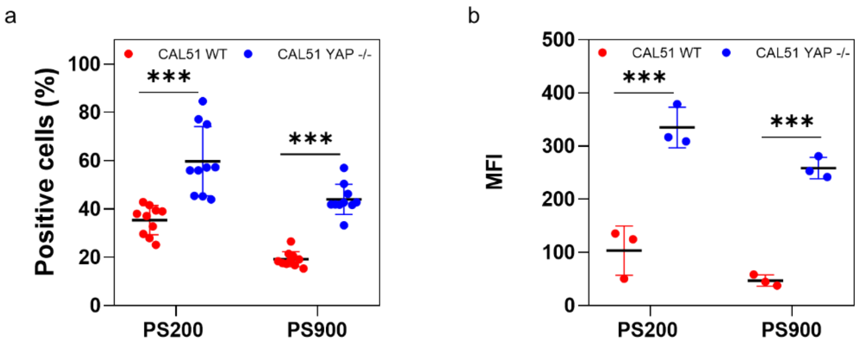

Figure S21. a) 4-hour cellular uptake of PS200 and PS900 in a serum-free medium for CAL51 WT (red) and CAL51 YAP -/- (blue) cells. Statistical analysis was performed using the two-way ANOVA followed by Sidak's multiple comparisons test. n = 9; *** indicates p < 0.001. b) Median fluorescence intensity (MFI) of 4-hour cellular uptake of PS200 and PS900 in a serum-free medium for CAL51 WT (red) and CAL51 YAP +/+ (blue) cells. Statistical analysis was performed using the two-way ANOVA followed by Tukey's multiple comparisons test. n = 3; *** indicates p < 0.001.

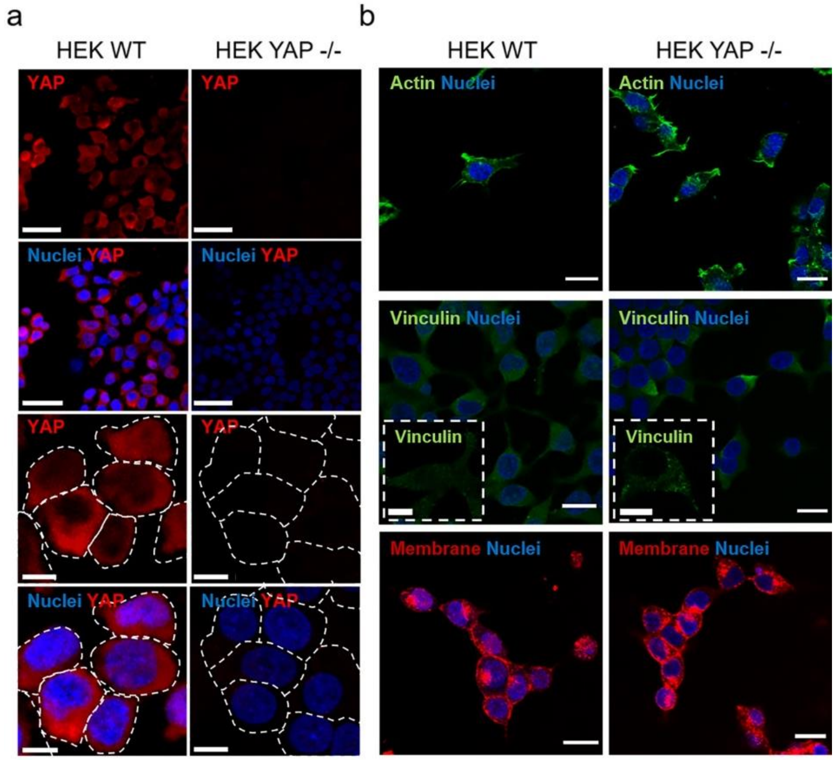

Figure S22. a) Representative confocal images depicting YAP expression in HEK WT or YAP -/- cells. Cells were stained for YAP (AF555), and nuclei were counterstained with DAPI. Scale bar: 50 µm. The green dashed line box shows higher magnification pictures. Scale bar: 10 µm. b) Representative confocal images of HEK WT or YAP -/- cells stained for DAPI and actin (Pha-488, green, top), vinculin (AF488, green, middle), and membrane (WGA-647, red, bottom), respectively. Scale bar: 20 µm. The insets display high magnification images. Scale bar: 10 µm.

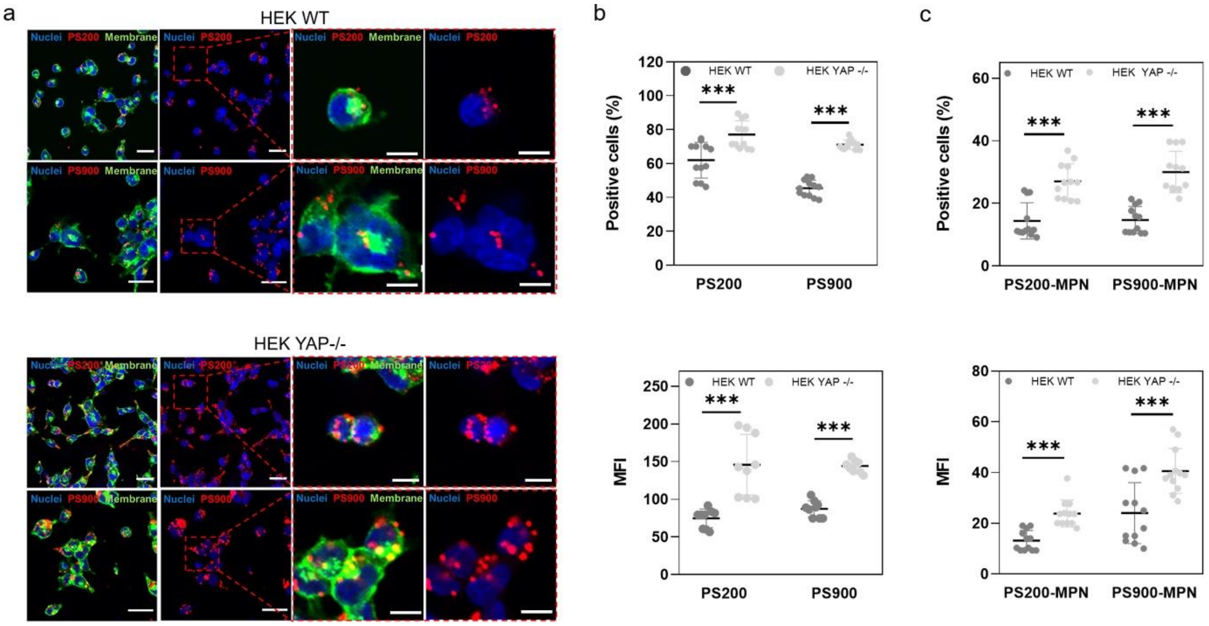

Figure S23. a) Confocal images of WT (top) and YAP -/- (bottom) HEK cells (right) after 4 hours of incubation with PS200 and PS900. Cells were stained with WGA-488 (green) and/or DAPI (blue). Magnified images are displayed inside the red dashed line boxes for each cell and particle type. Scale bar: 20 and 10 μm. b) 4-hour cellular uptake of PS200 and PS900 in WT (dark grey) and YAP -/- HEK (light grey) expressed as percentage of positive cells (top) and median fluorescence intensity (MFI, bottom). Statistical analysis was performed using the two-way ANOVA followed by Sidak's multiple comparisons test. n = 9; ***p < 0.001. c) 4-hour cellular uptake of PS200-MPN and PS900-MPN in WT (dark grey) and YAP -/- HEK (light grey) expressed as percentage of positive cells (top) and median fluorescence intensity (MFI, bottom). Statistical analysis was performed using the two-way ANOVA followed by Sidak's multiple comparisons test. n = 3; ***p < 0.001.

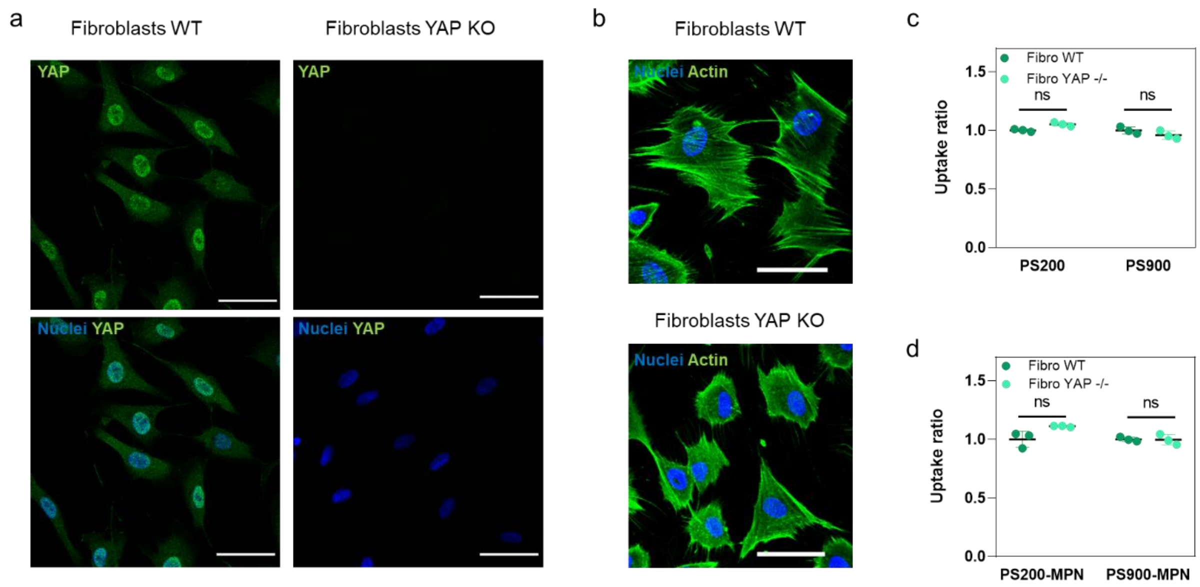

Figure S24. a) Representative confocal images depicting YAP expression in human fibroblasts WT or YAP -/- cells. Cells were stained for YAP (AF488), and nuclei were counterstained with DAPI. Scale bar: 50 µm. b) Representative confocal images of human fibroblasts WT (top) or YAP -/- (bottom) cells stained for DAPI and actin (Pha-488, green). Scale bar: 50 µm. c) 4-hour cellular uptake of PS200 and PS900 in Fibro WT (dark green) and Fibro YAP -/- (light green) cells. Statistical analysis was performed using the two-way ANOVA followed by Tukey's multiple comparisons test. n = 3; ns indicates non-significant. d) 4-hour cellular uptake of PS200-MPN and PS900-MPN in Fibro WT (dark green) and Fibro YAP -/- (light green) cells. Statistical analysis was performed using the two-way ANOVA followed by Tukey's multiple comparisons test. n = 3; ns indicates non-significant.

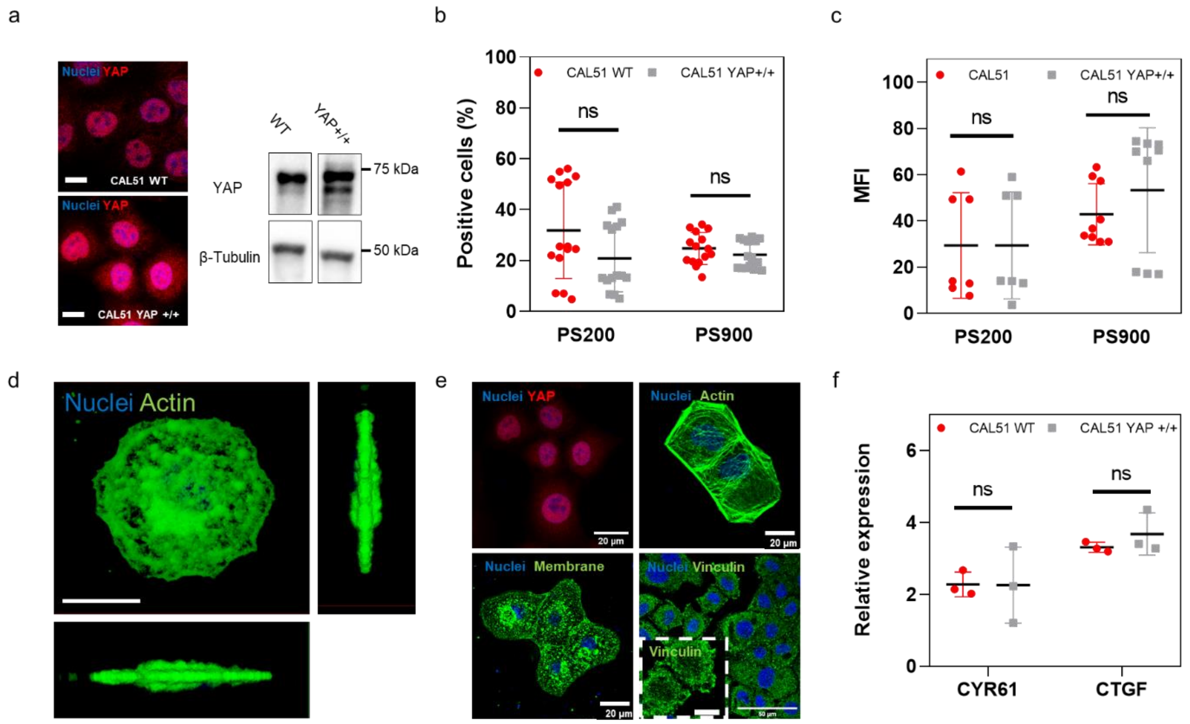

Figure S25. a) Confocal images of CAL51 WT cells (top) and CAL51 YAP +/+ cells, transfected with YAP (right). Cells are stained for YAP (AF555) and DAPI. Scale bar: 10 µm. A western blot on the right shows YAP levels in CAL51 WT and YAP +/+ cells. b) 4-hour cellular uptake of PS200 and PS900 in CAL51 WT (red) and CAL51 YAP +/+ (blue) cells. Statistical analysis was performed using the two-way ANOVA followed by Tukey's multiple comparisons test. n = 15; ns indicates non-significant. c) Median fluorescence intensity (MFI) of a 4-hour cellular uptake of PS200 and PS900 for CAL51 WT (red) and CAL51 YAP +/+ (blue) cells. Statistical analysis was performed using the two-way ANOVA followed by Tukey's multiple comparisons test. n = 6; ns indicates non-significant. d) 3D reconstruction of CAL51 YAP +/+ cells. The top and lateral views are presented. The cells are stained with DAPI (blue) and WGA-488 (green). Scale bar: 20 µm. e) Confocal images of CAL51 YAP +/+ cells stained for DAPI (blue), YAP (AF555, red, top left), actin (AF488, green, top right), membrane (WGA-647, red), and vinculin (AF488, green, bottom right). The bottom right panel shows vinculin staining in a magnified image inside the white dashed line box. Scale bars: 20 and 10 µm. f) Quantitative RT-PCR analysis of CYR61 and CTGF in CAL51 WT (red) and CAL51 YAP +/+ (grey) cells. Statistical analysis was performed using the unpaired t-test with Welch's correction. n = 3; ns indicates non-significant.

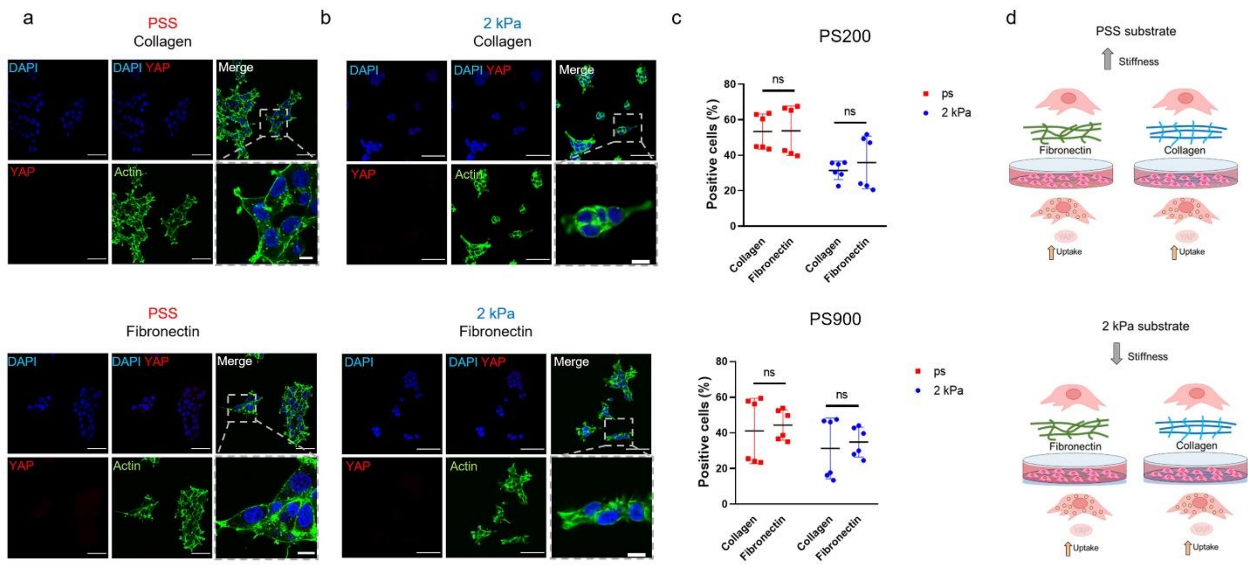

Figure S26. a) Confocal images of CAL51 YAP -/- cells grown on a standard polystyrene substrate coated with collagen (top) or fibronectin (bottom). Cells are stained with DAPI (blue) and Pha-488 (green), as well as for YAP (red). Magnified images are presented inside the gray dashed line boxes. Scale bars: 50 and 10 µm. b) Confocal images of CAL51 YAP -/- cells grown on 2 kPa soft substrate coated with collagen (top) or fibronectin (bottom). Cells are stained with DAPI (blue) and Pha-488 (green), as well as for YAP (red). Magnified images are presented inside the gray dashed line boxes. Scale bars: 50 and 10 µm. c) 4-hour cellular uptake of PS200 (top) and PS900 (bottom) for CAL51 YAP -/- cells grown on polystyrene (red) or soft 2 kPa (blue) substrate coated with collagen or fibronectin. Statistical analysis was performed using the two-way ANOVA followed by Sidak's multiple comparisons test. n = 3; ns is non-significant. d) When CAL51 YAP -/- cells are grown on polystyrene or soft substrates, nanoparticle uptake remains consistently higher compared to CAL5 WT cells, regardless of the coating material used for the substrate.

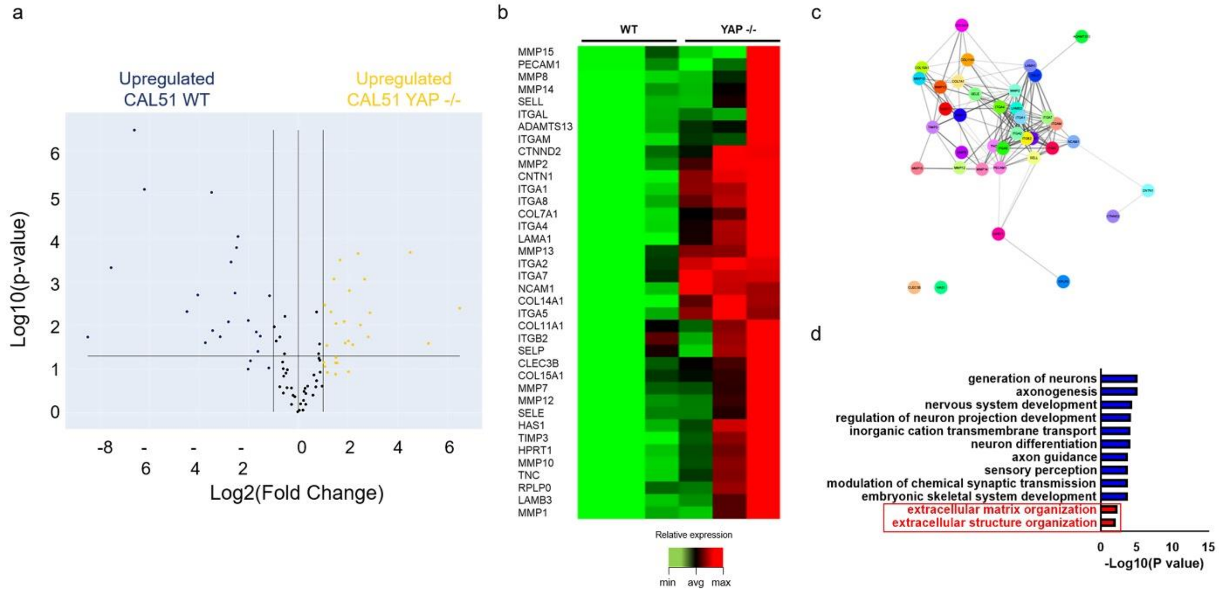

Figure S27. a) Volcano plot representation of the differentially regulated genes in CAL51 WT vs. CAL51 YAP -/- cells. Blue points indicate significantly upregulated genes in CAL51 WT, and yellow points indicate upregulated genes in CAL51 YAP -/-. n = 12 (P adj < 0.05, log2Fc > |1|). b) RT^2-profiler PCR array for the ECM and adhesion molecules overexpressed in CAL51 YAP -/- compared to CAL51 WT (P adj < 0.05, log2Fc > |1|). c) STRING PPI network of the differently expressed ECM proteins in CAL51 YAP -/- obtained from Cytoscape (P adj < 0.05, log2Fc > |2|, confidence cutoff 0.4). d) Bar plot representation of common enriched biological processes and pathways obtained from the ENRICHR database [7-9], presenting the most significantly upregulated genes in CAL51 YAP -/- compared to CAL51 WT. Annotations directly related to ECM organization are highlighted in the red box (P adj < 0.05, log2Fc > |2|).

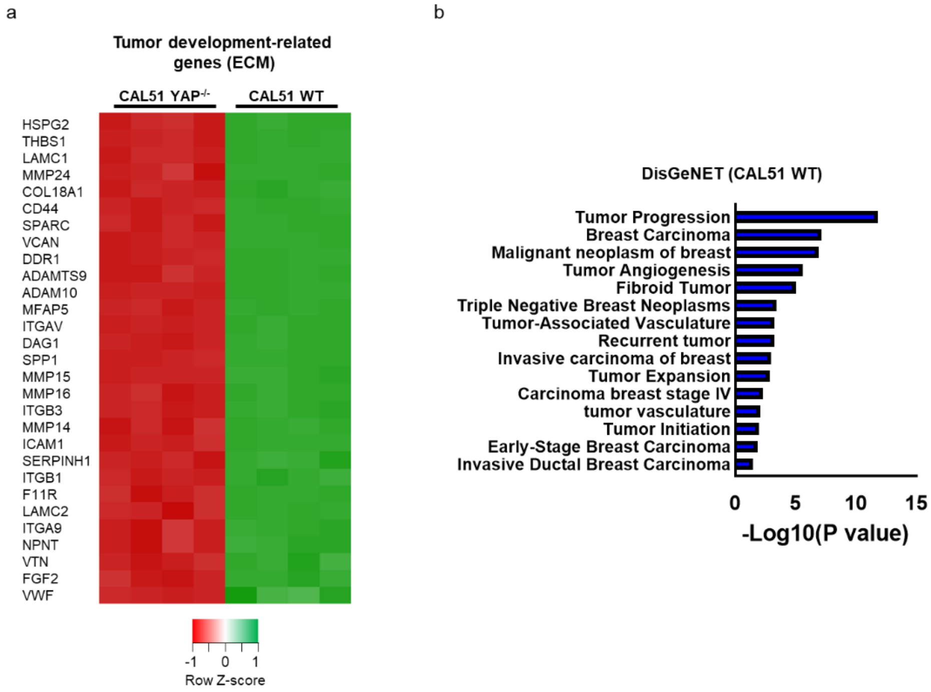

Figure S28. a) Heatmap of the relative expression of genes related to ECM components and involved in tumor development (P adj < 0.05, log2Fc > |2|). b) Bar plot representation of common enriched pathways obtained from the DisGeNET database, presenting the most significantly upregulated genes in CAL51 WT compared to CAL51 YAP -/- cells (P adj < 0.05, log2Fc > |2|).

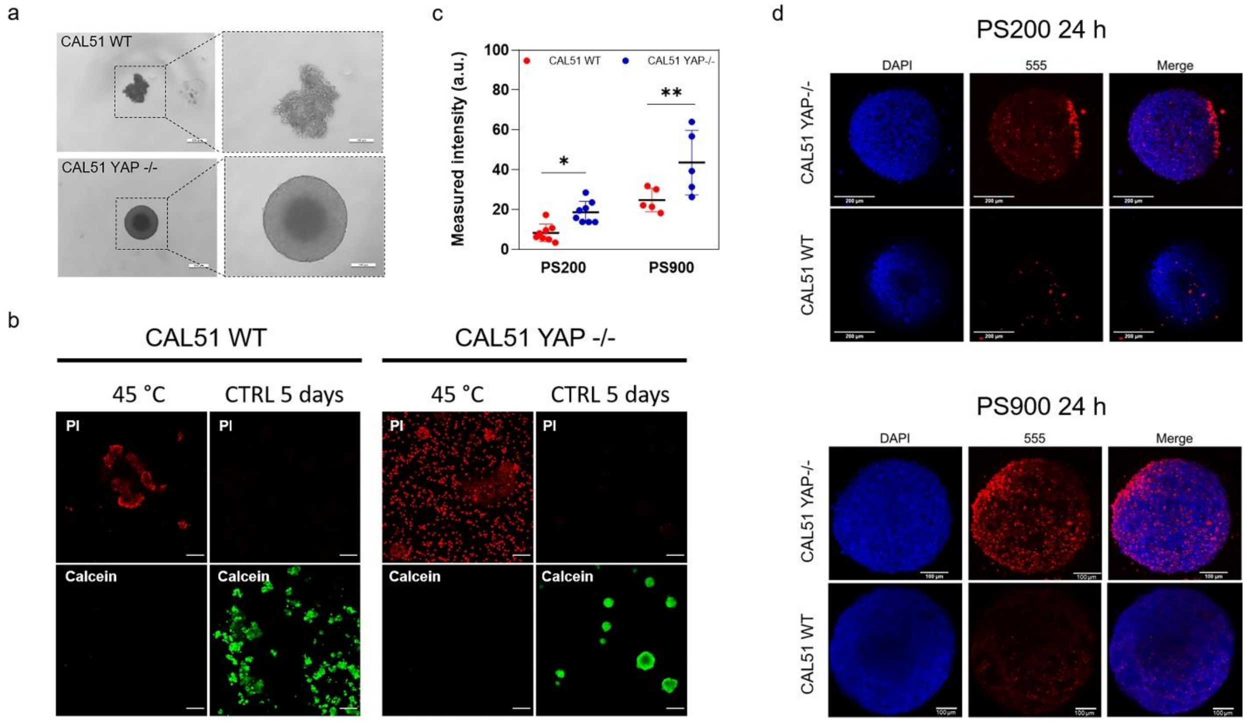

Figure S29. a) Representative brightfield images of CAL51 WT and CAL51 YAP -/- spheroids seeded onto 96-well round (U) bottom plates 5 days after seeding. Magnified images are presented inside the black dashed line boxes. Scale bars: 200 and 100 µm. b) The live/dead assay performed on CAL51 WT and CAL51 YAP -/- cells. As a control for cell death, spheroids were heated for 10 minutes at 45 °C. Cells were excited with 555 nm laser (propidium iodide, PI, red) and 488 nm laser (calcein, green). c) Nanoparticle intensity per cell after 4-hour incubation of CAL51 WT and YAP -/- cells with PS200 and PS900. Statistical analysis was performed using the two-way ANOVA followed by Sidak's multiple comparisons test. n > 5; * indicates $p < 0.05$, ** indicates $p < 0.01$.

d) Confocal images of CAL51 YAP -/- and CAL51 WT spheroids incubated with PS200 and PS900 for 24 hours. Cells are stained with DAPI (blue). Nanoparticles are displayed in red. Scale bars: 100 and 200 µm.

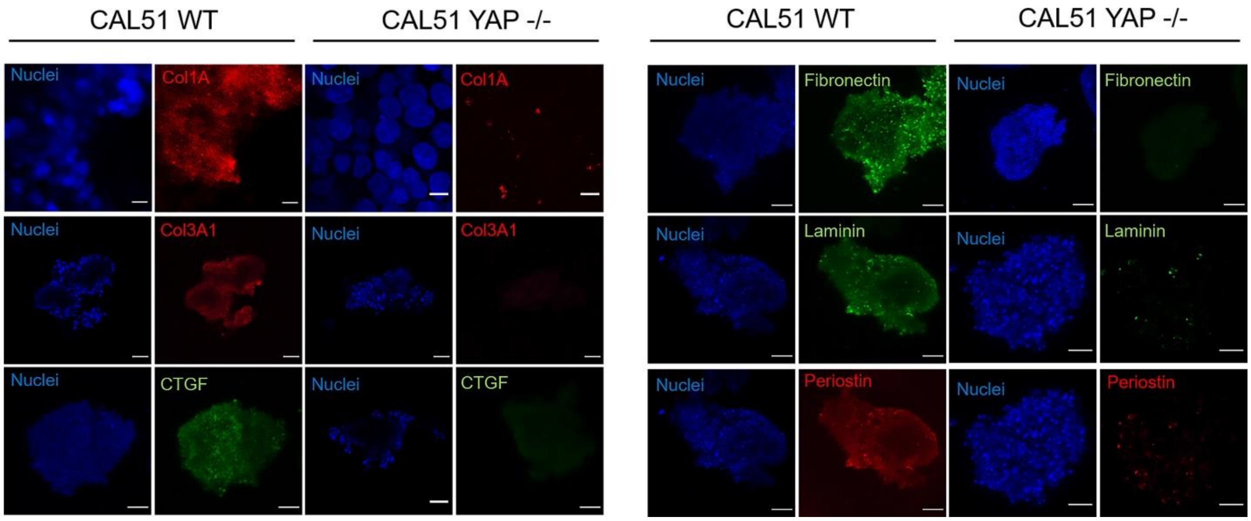

Figure S30. Confocal images of ECM components for the CAL51 WT (left) and YAP -/- (right) spheroids. Collagen type 1 alpha (Col1A), collagen type III alpha 1 (Col3A1), connective tissue growth factor (CTGF), and periostin are stained with II-antibody labeled with AF-555 (red); fibronectin and laminin are stained with II-antibody labeled with AF-488 (green). Scale bar: 100 µm.

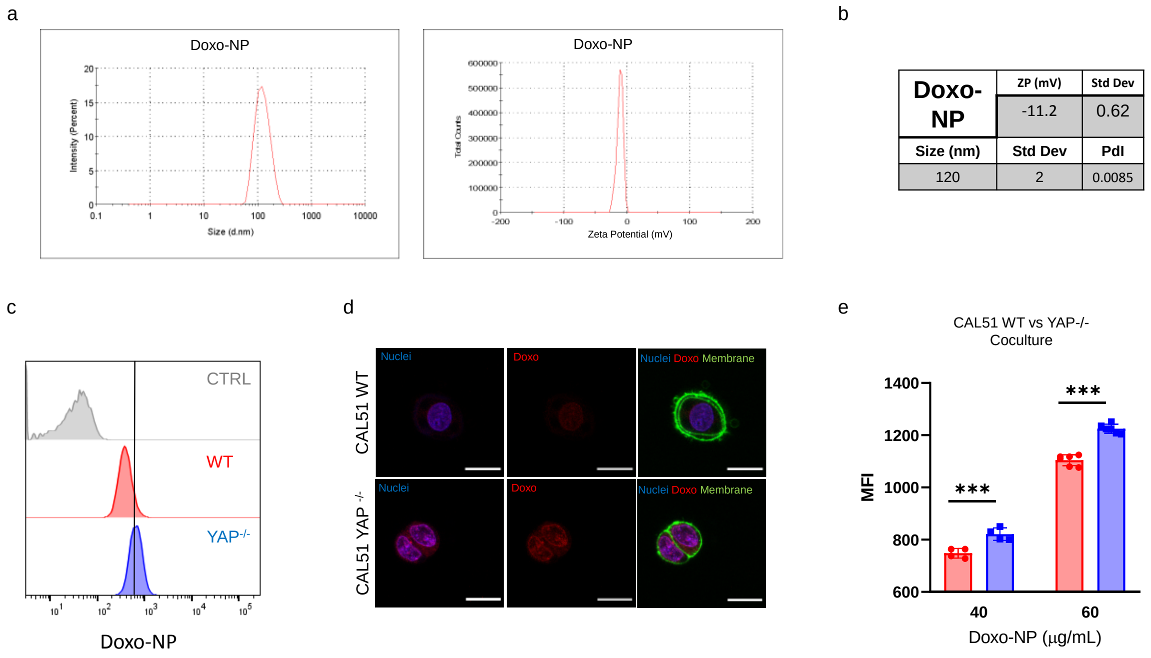

Figure S31. a) DLS graph showing size weighted by intensity (left) and the zeta potential (right) of Doxo-NP in 10 mM PBS. b) Table reporting the zeta potential (ZP), hydrodynamic diameter (dH), and polydispersity index (PdI) of Doxo-NP. c) Representative histograms of CAL51 WT (red) and YAP -/- (blue) cells incubated for 4 hours with Doxo-NP. d) Confocal images of CAL51 WT (top) and CAL51 YAP -/- (bottom) cells after 8-hour incubation with Doxo-NP. Cells are stained with WGA-488 (green) and DAPI (blue). Nanoparticles are displayed in red. Scale bar: 20 µm. e) Median fluorescence intensity (MFI) of 4-hour cellular uptake of Doxo-NP at different concentrations (40 and 60 µg/mL) for CAL51 WT (red) and CAL51 YAP -/- (blue) cells in co-culture. Statistical analysis was performed using the two-way ANOVA followed by Sidak's multiple comparisons test. n = 6; *** indicates $p < 0.001$.

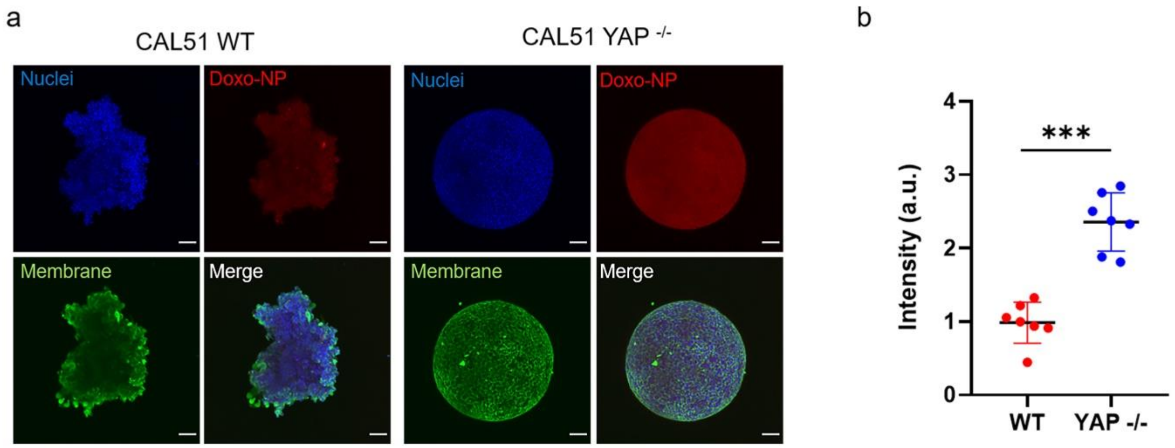

Figure S32. a) Confocal images of spheroids from CAL51 WT (left) and YAP -/- (right) cells incubated for 4 hours with Doxo-NP. Cells are stained with WGA-488 (green) and DAPI (blue). Nanoparticles are displayed in red. Scale bar: 100 µm. b) Nanoparticle intensity per spheroid after 4-hour incubation of CAL51 WT (red) and YAP -/- (blue) spheroids with Doxo-NP. Statistical analysis was performed using the unpaired t-test with Welch's correction. $n = 7$; *** indicates $p < 0.001$.

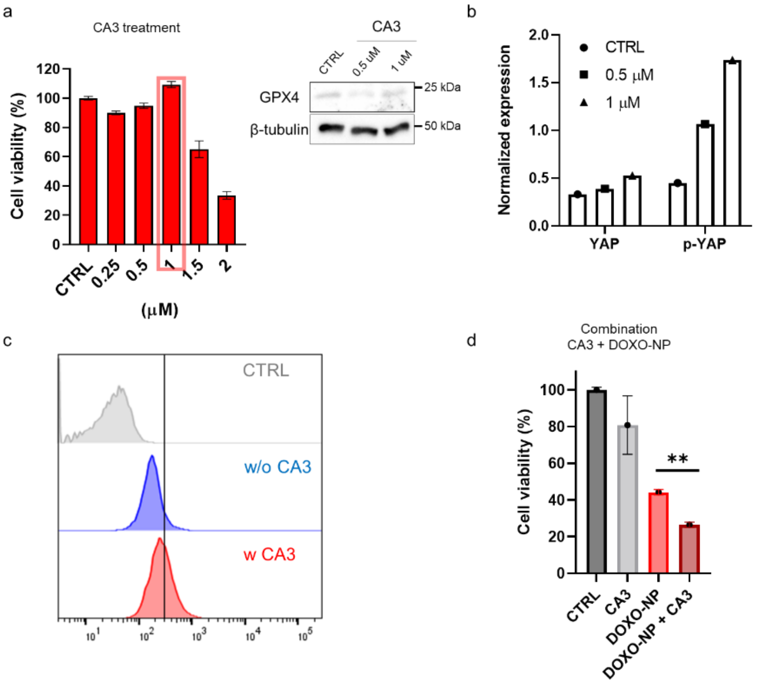

Figure S33. a) Presto Blue viability assay on CAL51 WT cells treated with different concentrations of CA3 (0.25/0.5/1/1.5/2 µM) for 12 hours. The red box indicates the concentration chosen for the follow up experiments. n = 8. On the right, western blot showing the levels of ferroptosis marker GPX4 in CAL51 WT untreated (CTRL) or treated with 0.5 and 1 µM CA3 inhibitor for 12 hours. β-tubulin was used for protein loading normalization. b) Normalized expression of YAP and p-YAP levels obtained from western blot analysis on CAL51 WT untreated or treated with 0.5 or 1 µM CA3. β-Tubulin was used for protein normalization. c) Representative histograms of CAL51 WT cells untreated (blue) or treated (red) with 1 µM CA3 for 12 hours and incubated for 4 hours with Doxo-NP. d) Presto Blue viability assay on CAL51 WT cells untreated (red) or treated for 12 hours with 1 µM CA3 and subsequently incubated for 4 hours with Doxo-NP. Cell viability was assessed 48 hours post-treatment and normalized to untreated cells (control). Statistical analysis was performed using one-way ANOVA with followed by Tukey's multiple comparisons test. n = 8; ** indicates $p < 0.01$.

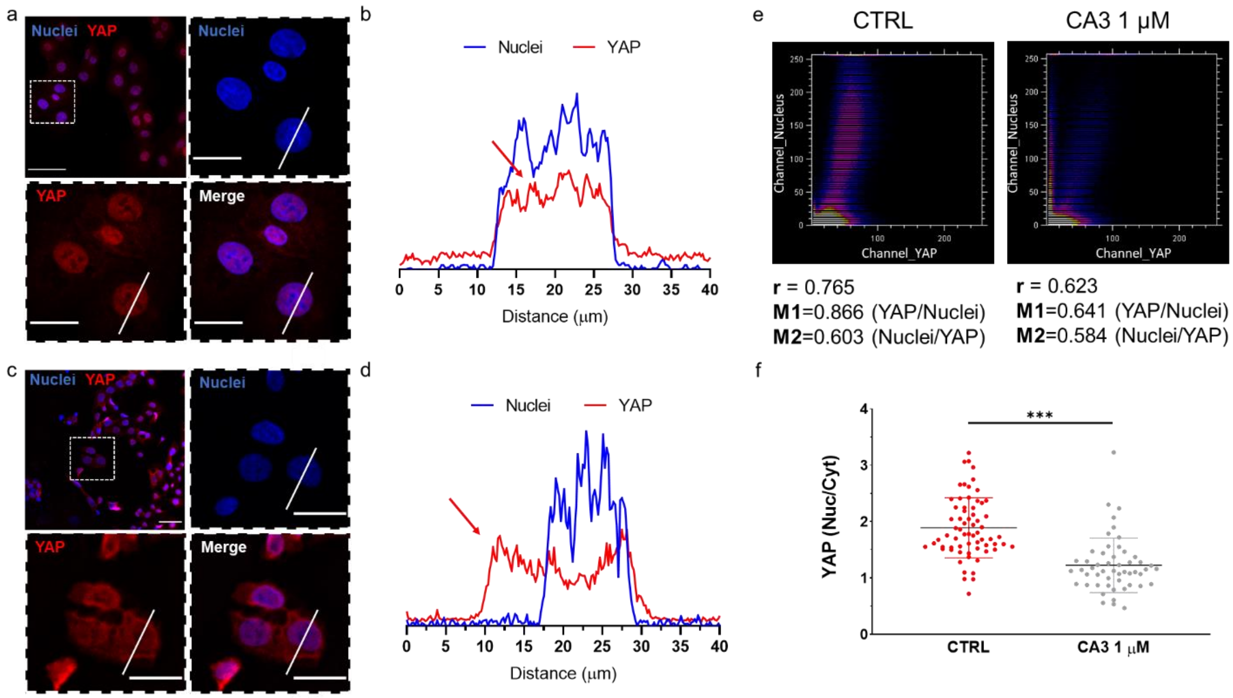

Figure S34. Representative confocal images of CAL51 WT cells (a) untreated or (c) treated with 1 μM CA3 for 12 hours. Scale bars: 50 (for the top-left corner picture) and 20 μm. b, d) Corresponding plot profiles of YAP signal intensity (AF555, red) co-localized with the nucleus (blue, DAPI), extracted from the area on the images a and c highlighted with white dashed lines, in the high magnification. e) Scatter plots of YAP and nuclei colocalization events for CAL51 WT untreated (left) and treated (right) with 1 μM CA3. Pearson's coefficients (r) and Mander's coefficients (M) are shown below each graph. The analysis was performed using Fiji's plugins Jacop and Colocalization finder. f) Dot plot representation of the YAP nuclear/cytoplasmic ratio in CAL51 WT cells untreated (red) or treated with 1 μM CA3 (grey). Statistical analysis was performed by unpaired t-test with Welch's correction. n > 50; ***indicates p < 0.001.

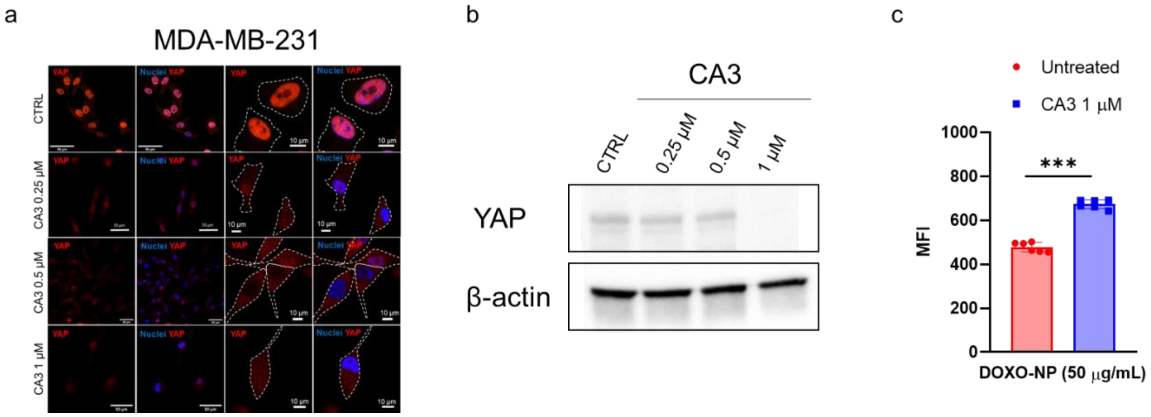

Figure S35. a) Confocal images of MDA-MB-231 treated with different concentration of YAP inhibitor CA3 (0.25/0.5/1 μM) for 12 hours. The cells are stained for YAP1 (AF555, red) and DAPI (blue). Close-up pictures are shown on the right, were white dashed lines define cell's perimeter. Scale bars are 50 and 10 µm. b) Western blot showing the levels of YAP in MDA-MB-231 cells untreated (CTRL) or treated with 0.25, 0.5 and 1 μM CA3 inhibitor for 12 hours. β-tubulin was used for protein loading normalization. c) Median fluorescence intensity (MFI) of 4-hours cellular uptake of Doxo-NP for MDA-MB-231 untreated (red) or treated (blue) for 12 hours (before cell-nanoparticle interactions assessment) with CA3 1 μM. Statistical analysis was performed by unpaired t-test with Welch's correction. n = 6; *** indicates $p < 0.001$.

Supplementary Table S1. List of used immunofluorescence antibodies and their relative dilution.

Antibody	Dilution	Buffer	Reference	Supplier
YAP1	1:200	(A)	(63.7) sc-101199	Santa Cruz
Vinculin	1:200	(A)	V9131	Merck
Collagen 1A1	1:100	(B)	91144S	Cell Signaling
Collagen 3A1	1:100	(B)	PA5-27828	Thermo Fisher
CTGF	1:100	(B)	Ab5097	Abcam
Fibronectin	1:100	(B)	F3648	Merck
Laminin	1:25	(B)	SAB4200719	Merck
Periostin	1:100	(B)	Sc-49480	Santa Cruz

(A) 2.5 % BSA, PBS, 2h (RT) – O.N. (4 °C)

(B) 2.5 % BSA, PBS, O.N. (4 °C)

Minimum Information Reporting in Bio−Nano Experimental Literature [10].

Supplementary Table S2. Material characterization

Question	Yes	No
1.1 Are "**best reporting practices**" **available** for the nanomaterial used?	X	
1.2 If they are available, **are they used**? If not available, ignore this question and proceed to the next one.		
1.3 Are extensive and clear instructions reported detailing all steps of **synthesis** and the resulting **composition** of the nanomaterial?	X	
1.4 Is the **size** (or **dimensions**, if non-spherical) and **shape of** the nanomaterial reported?	X	
1.5 Is the **size dispersity** or **aggregation** of the nanomaterial reported?	X	
1.6 Is the **zeta potential** of the nanomaterial reported?	X	
1.7 Is the **density (mass/volume)** of the nanomaterial reported?		X
1.8 Is the amount of any **drug loaded** reported? 'Drug' here broadly refers to functional cargos (e.g., proteins, small molecules, nucleic acids).		X
1.9 Is the **targeting performance** of the nanomaterial reported, including **amount** of ligand bound to the nanomaterial if the material has been functionalised through addition of targeting ligands?		X
1.10 Is the **label signal** per nanomaterial/particle reported? For example, fluorescence signal per Particle for fluorescently labelled nanomaterials.	X	
1.11 If a material property not listed here is varied, has it been **quantified**?		NA
1.12 Were characterizations performed in a **fluid mimicking biological conditions**?	X	
1.13 Are details of how these parameters were **measured/estimated** provided?	X	
Explanation for **No** (if needed):		

Supplementary Table S3. Biological characterization

Question	Yes	No
2.1 Are **cell seeding details**, including **number of cells plated, confluency at start of Experiment,** and **time between seeding and experiment** reported?	X	
2.2 If a standardised cell line is used, are the **designation and source** provided?	X	
2.3 Is the **passage number** (total number of times a cell culture has been subcultured) known and reported?	X	

2.4 Is the last instance of **verification of cell line** reported? If no verification has been performed, is the time passed and passage number since acquisition from trusted source (e.g., ATCC or ECACC) reported?		X
2.5 Are the results from **mycoplasma testing** of cell cultures reported?		X
2.6 Is the **background signal of cells/tissue** reported? (E.g., the fluorescence signal of cells without particles in the case of a flow cytometry experiment.)	X	
2.7 Are **toxicity studies** provided to demonstrate that the material has the expected toxicity, and that the experimental protocol followed does not?	X	
2.8 Are details of media preparation (**type of media, serum,** any **added antibiotics**) provided?	X	
2.9 Is a **justification of the biological model** used provided?	X	
2.10 Is characterization of the **biological fluid** (*ex vivo*/*in vitro*) reported? For example, when investigating protein adsorption onto nanoparticles dispersed in blood serum, pertinent aspects of the blood serum should be characterised (e.g., protein concentrations and differences between donors used in study).	X	
2.11 For **animal experiments**, are the ARRIVE guidelines followed?		NA
Explanation for **No** (if needed):		

Supplementary Table S4. Experimental details

**Question**	**Yes**	**No**
3.1 For cell culture experiments: are **cell culture dimensions** including **type of well**, **volume of added media**, reported? Are cell types (i.e.; adherent vs suspension) and **orientation** (if non- standard) reported?	X	
3.2 Is the **dose of material administered** reported? This is typically provided in nanomaterial mass, volume, number, or surface area added. Is sufficient information reported so that regardless of which one is provided, the other dosage metrics can be calculated (i.e. using the dimensions and density of the nanomaterial)?	X	
3.3 For each type of imaging performed, are details of how **imaging** was performed provided, including details of **shielding, non-uniform image processing**, and any **contrast agents** added?	X	

3.4 Are details of how the dose was administered provided, including **method of administration, injection location, rate of administration,** and details of **multiple injections**?	X	
3.5 Is the methodology used to **equalise dosage** provided?	X	
3.6 Is the **delivered dose** to tissues and/or organs (in vivo) reported, as % injected dose per gram of tissue (%ID g^{-1})?		NA
3.7 Is **mass of each organ/tissue measured** and **mass of material** reported?		NA
3.8 Are the **signals of cells/tissues with nanomaterials** reported? For instance, for fluorescently labelled nanoparticles, the total number of particles per cell or the fluorescence intensity of particles + cells, at each assessed timepoint.	X	
3.9 Are **data analysis details**, including **code used** for analysis provided?	X	
3.10 Is the **raw data** or **distribution of values** underlying the reported results provided?	X	
Explanation for **No** (if needed):		

Supplementary Materials References

[1] G. Nardone, J. Oliver-De La Cruz, J. Vrbsky, C. Martini, J. Pribyl, P. Skládal, M. Pešl, G. Caluori, S. Pagliari, F. Martino, Z. Maceckova, M. Hajduch, A. Sanz-Garcia, N.M. Pugno, G.B. Stokin, G. Forte, YAP regulates cell mechanics by controlling focal adhesion assembly, Nature Communications, 8 (2017) 15321

[2] H. Qin, K. Blaschke, G. Wei, Y. Ohi, L. Blouin, Z. Qi, J. Yu, R.F. Yeh, M. Hebrok, M. Ramalho-Santos, Transcriptional analysis of pluripotency reveals the Hippo pathway as a barrier to reprogramming, Human molecular genetics, 21 (2012) 2054-2067.

[3] B.V. Derjaguin, V.M. Muller, Y.P. Toporov, Effect of contact deformations on the adhesion of particles, Journal of Colloid and Interface Science, 53 (1975) 314-326.

[4] P. Hermanowicz, M. Sarna, K. Burda, H. Gabryś, AtomicJ: An open source software for analysis of force curves, 85 (2014) 063703.

[5] X. Yang, J.S. Boehm, X. Yang, K. Salehi-Ashtiani, T. Hao, Y. Shen, R. Lubonja, S.R. Thomas, O. Alkan, T. Bhimdi, T.M. Green, C.M. Johannessen, S.J. Silver, C. Nguyen, R.R. Murray, H. Hieronymus, D. Balcha, C. Fan, C. Lin, L. Ghamsari, M. Vidal, W.C. Hahn, D.E. Hill, D.E. Root, A public genome-scale lentiviral expression library of human ORFs, Nature methods, 8 (2011) 659-661.

[6] D. Torre, A. Lachmann, A. Ma'ayan, BioJupies: Automated Generation of Interactive Notebooks for RNA-Seq Data Analysis in the Cloud, Cell systems, 7 (2018) 556-561.e553.

[7] E.Y. Chen, C.M. Tan, Y. Kou, Q. Duan, Z. Wang, G.V. Meirelles, N.R. Clark, A. Ma'ayan, Enrichr: interactive and collaborative HTML5 gene list enrichment analysis tool, BMC Bioinformatics, 14 (2013) 128.

[8] M.V. Kuleshov, M.R. Jones, A.D. Rouillard, N.F. Fernandez, Q. Duan, Z. Wang, S. Koplev, S.L. Jenkins, K.M. Jagodnik, A. Lachmann, M.G. McDermott, C.D. Monteiro, G.W. Gundersen, A. Ma'ayan, Enrichr: a comprehensive gene set enrichment analysis web server 2016 update, Nucleic acids research, 44 (2016) W90-97.

[9] Z. Xie, A. Bailey, M.V. Kuleshov, D.J.B. Clarke, J.E. Evangelista, S.L. Jenkins, A. Lachmann, M.L. Wojciechowicz, E. Kropiwnicki, K.M. Jagodnik, M. Jeon, A. Ma'ayan, Gene Set Knowledge Discovery with Enrichr, Current protocols, 1 (2021) e90.

[10] M. Faria, M. Björnmalm, K.J. Thurecht, S.J. Kent, R.G. Parton, M. Kavallaris, A.P.R. Johnston, J.J. Gooding, S.R. Corrie, B.J. Boyd, P. Thordarson, A.K. Whittaker, M.M. Stevens, C.A. Prestidge, C.J.H. Porter, W.J. Parak, T.P. Davis, E.J. Crampin, F. Caruso, Minimum information reporting in bio-nano experimental literature, Nature nanotechnology, 13 (2018) 777-785.
